# The Acute Effects of Short-Bout High-Intensity Interval Training on Physiological and Perceptual Outcomes: A Systematic Review and Meta-analysis

**DOI:** 10.1007/s40279-026-02456-x

**Published:** 2026-05-22

**Authors:** Fraser Thurlow, Nicholas Cowley, Katie Slattery, Jonathon Weakley

**Affiliations:** 1 School of Sport, Exercise and Rehabilitation, University of Technology Sydney, UTS-Rugby Australia Building, Moore Park Rd, Sydney, NSW 2021 Australia; 2 School of Behavioural and Health Sciences, Australian Catholic University, Brisbane, QLD Australia; 3 Sports Performance, Recovery, Injury and New Technologies (SPRINT) Research Centre, Australian Catholic University, Brisbane, QLD Australia; 4 Carnegie Applied Rugby Research (CARR) Centre, Carnegie School of Sport, Leeds, UK

## Abstract

**Background:**

Understanding the acute demands of short-bout high-intensity interval training can enhance training outcomes.

**Objective:**

We aimed to examine the acute physiological and perceptual demands of short-bout high-intensity interval training in athletes and identify how they are moderated by programming variables, fitness level and competitive level.

**Methods:**

We searched the databases PubMed, SPORTDiscus and CINAHL on 2 December, 2025 for original research articles investigating running-based, short-bout high-intensity interval training in healthy athletes, aged 16–40 years, of any sex, who were recreationally active or above. Outcomes were analysed using a multi-level mixed-effects meta-analysis. The analysed outcomes were: average heart rate (HR_avg_), peak heart rate (HR_peak_), peak and average oxygen consumption (*V*O_2_), time > 90% of maximal oxygen consumption (*T* > 90% *V*O_2max_), time > 95% of *V*O_2max_ (*T* > 95% *V*O_2max_), *T* > 90% *V*O_2max_/exercise time ratio (*T*_90_/ET), *T* > 95% *V*O_2max_/exercise time ratio (*T*_95_/ET), blood lactate concentration (B[La]) and session ratings of perceived exertion (sRPE). To examine the influence of programming variables, fitness level, and competitive level, a meta-regression was performed on moderators with ten or more samples. Effects were evaluated based on coverage of their confidence limits against elected thresholds of practical importance.

**Results:**

From 139 data samples within 46 studies, the pooled demands (± 90% confidence limit) of short-bout high-intensity interval training were: HR_avg_, 169 ± 4 b·min^−1^ and 88 ± 2% of maximum heart rate; HR_peak_, 184 ± 4 b·min^−1^ and 94 ± 2% of maximum heart rate; average *V*O_2_, 46 ± 3 mL·kg^−1^·min^−1^ and 78 ± 4% of velocity at *V*O_2max_; peak *V*O_2_, 55 ± 5 mL·kg^−1^·min^−1^ and 93 ± 7% of *V*O_2max_; *T* > 90% velocity at *V*O_2max_, 259 ± 57 s; time > 95% *V*O_2max_, 125 ± 48 s; *T*_90_/ET, 0.34 ± 0.9; *T*_95_/ET, 0.15 ± 0.07; B[La], 8.3 ± 0.5 mmol·L^−1^; and sRPE, 7.1 ± 0.5 au. When compared to an athlete *V*O_2max_ of 50–55 mL·kg^−1^·min^−1^, a *V*O_2max_ of 55–60 mL·kg^−1^·min^−1^ and > 60 mL·kg^−1^·min^−1^ were associated with a substantial decrease in sRPE (− 1.7 ± 1.3 au and –1.6 ± 1.1 au, respectively). Compared to a reference protocol of 1 set of 12 straight-line repetitions, performed at 120% of maximal aerobic speed for a 15-s work duration with 15 s of passive rest: shuttle runs were associated with a substantial increase in B[La] (1.9 ± 1.0 mmol·L^−1^) and sRPE (1.7 ± 0.4 au); active rest was associated with a substantial increase in *T*_90_/ET (0.21 ± 0.11); performing a 15-s longer repetition was associated with a substantial increase in *T* > 90% velocity at *V*O_2max_ (160 ± 113 s); completing one more set was associated with a substantial increase in B[La] (1.2 ± 0.1 mmol·L^−1^); and a 5% increase in maximal aerobic speed was associated with a substantial increase in B[La] (1.0 ± 0.3 mmol·L^−1^), HR_avg_ (3.2 ± 1.4 b·min^−1^), and *T*_90_/ET (0.05 ± 0.04), but a substantial decrease in *T* > 95% *V*O_2max_ (− 44 ± 28 s). All other moderators had compatibility with trivial effects or were inconclusive.

**Conclusions:**

Short-bout high-intensity interval training elicits a substantial physiological stimulus, which is influenced by programming variables, athlete fitness level and competitive level. Implementing an active rest period, longer repetition duration and a higher running intensity are effective strategies to maximise the aerobic stimulus, while shuttle runs increase the anaerobic demand.

**Supplementary Information:**

The online version contains supplementary material available at https://doi.org/10.1007/s40279-026-02456-x.

## Key Points

**Table:** 

The pooled-effect estimates for average heart rate (88% of maximum heart rate), average oxygen consumption (78% of maximal oxygen consumption [$\dot{V}$O_2max_]), time spent above 90% of $\dot{V}$O_2max_ (259 s), and blood lactate (8.3 mmol·L^−1^) highlight the substantial physiological demands of short-bout high-intensity interval training in athletes
The reference estimate of session ratings of perceived exertion (7.1 au; ‘very hard’) demonstrates the perceptual demand of short-bout high-intensity interval training, though this demand was considerably lower in fitter athletes with a $\dot{V}$O_2max_ of > 55 mL·kg^−1^·min^−1^.
Compared with 1 set of 12 straight-line repetitions, performed at 120% of maximal aerobic speed with a 15-s work duration and 15 s of passive rest, incorporating an active rest period, extending repetition duration to 30 s with 30 s of rest, or increasing repetition intensity by 5% of maximal aerobic speed are effective strategies to maximise time spent above 90% of $\dot{V}$O_2max_ and improve training efficiency (time spent above 90% $\dot{V}$O_2max_/exercise time ratio).
Shuttle runs are the most effective prescription to maximise the anaerobic demand of short-bout high-intensity interval training, but they also increased session ratings of perceived exertion by 1.7 ± 0.4 au.
Prescribing 6–12 repetitions per set provides a substantial physiological stimulus, but adding two more repetitions per set has a trivial impact.
Our findings provide insights into the expected demands of short-bout high-intensity interval training across various athlete characteristics, offering guidance to optimise training prescription through strategic manipulation of programming variables.

## Introduction

High-intensity interval training (HIIT) is an effective method for improving physical fitness [1, 2]. There are several HIIT formats, including long-bout HIIT, short-bout HIIT (short-HIIT), repeated-sprint training and sprint interval training, each of which can be performed using various exercise modalities (e.g. running, cycling) [3, 4]. Short-HIIT is commonly implemented as a running-based intervention for both intermittent sport and endurance athletes [5–9]. This format involves repeated efforts lasting less than 60 s with similar work-to-rest ratios (e.g. 1:1), that are typically performed at or above an athlete’s maximal aerobic speed (MAS) [10]. Sessions are generally completed in under 30 min and elicit substantial volumes of high-speed running that is performed near-maximal oxygen consumption ($\dot{V}$O_2max_) [11–13]. This stimulus drives key physiological and neuromuscular adaptations, including improvements in $\dot{V}$O_2max_, time to exhaustion, sprint times, repeated-sprint ability, countermovement jump height and intermittent running performance [5, 6, 14–16]. Given their sports' intermittent nature and need for energy system development in a limited training time, team and racket sport athletes may particularly benefit from short-HIIT.

High-intensity interval training programs can be designed to target various outcomes through manipulation of programming variables, which form the foundation of training design [17–19]. Short-HIIT can be performed with either linear or shuttle runs and passive or active rest periods, with repetitions completed across a range of intensities (e.g. 100–140% MAS) and structured in shorter (e.g. 4 sets of 6 repetitions) or longer series (e.g. 2 sets of 12 repetitions). Although a variation in training design allows for flexible programming options, it also leads to diverse internal loads. For example, in a study by Bok et al. [20], when athletes completed a 20 × 15-s interval at 110% of the final velocity achieved during the multi-stage 20-m shuttle run test (20mSRT) average $\dot{V}$O_2_, blood lactate concentration (B[La]) and session ratings of perceived exertion (sRPE) were 84 ± 6% of $\dot{V}$O_2max_, 12 ± 2 mmol·L^−1^ and 9.5 ± 0.5 CR10^®^ units, respectively. When the same session was performed at 100% of the 20mSRT end velocity, the physiological stimulus was reduced, as demonstrated by an average $\dot{V}$O_2_ of 78 ± 6% of $\dot{V}$O_2max_, B[La] of 5 ± 3 mmol·L^−1^ and sRPE of 6.6 ± 1.0 au [20]. Variation in the acute physiological and perceptual demands of short-HIIT has also been observed when comparing the running modality, the number of sets and repetitions, repetition duration and rest modality [7, 13, 15, 21–24]. As chronic adaptations are driven by the physiological responses to training, it is crucial to understand how programming differences influence the acute demands of short-HIIT.

To maximise cardiovascular and peripheral adaptations from HIIT, it has been recommended that athletes accumulate several minutes per session above 90% of $\dot{V}$O_2max_ [1, 25, 26]. Improvements in endurance performance following interval training are positively associated with both the fraction achieved and the time spent above 90% of $\dot{V}$O_2max_ (*T* > 90% $\dot{V}$O_2max_) [27]. Additionally, higher exercise intensities promote greater recruitment of type II fibres and near-maximal cardiac output, which subsequently enhances oxidative muscle fibre adaptation and myocardial enlargement [3, 28, 29]. Several studies have measured the $\dot{V}$O_2_ demands of short-HIIT [8, 9, 13, 15, 20, 30–46], yet the results vary widely, with *T* > 90% $\dot{V}$O_2max_ and an average $\dot{V}$O_2_ range from 0–12 min and 60–90% of $\dot{V}$O_2max_, respectively. These discrepancies are largely due to differences in training protocols, such as repetition duration and rest modality, which impact session effectiveness and efficiency. Understanding how programming variables influence time spent near $\dot{V}$O_2max_ will help practitioners optimise the physiological stimulus of short-HIIT. In addition, psychophysiological variables such as anaerobic glycolytic energy contribution and sRPE should also be considered to fully characterise the internal load.

The design of training programmes depends on various contextual factors, such as athlete fitness level, competitive level, training status, season stage and training goals [47]. Athletes with different physiological profiles may respond differently to HIIT [32, 48, 49]. For instance, Cipryan et al. [32] showed that athletes with a higher $\dot{V}$O_2max_ rely more on aerobic metabolic pathways during short-HIIT, while less fit athletes experience greater anaerobic glycolytic stress. Furthermore, national-level basketball players exhibited a lower peak heart rate (HR_peak_) and B[La] during repeated sprints than players at a trained level [50]. Without adjusting for differences in fitness and competitive level, athletes may experience inconsistent training loads, potentially compromising the intended training effects. To optimise outcomes, further research is needed on how fitness level and performance calibre influence the acute demands of short-HIIT. Combined with an understanding of how programming variables affect the internal load, coaches can strategically adjust training to elicit the desired psychophysiological responses. Therefore, our systematic review and meta-analysis aimed to examine the acute physiological and perceptual demands of short-HIIT and identify how these demands are moderated by programming variables, athlete fitness level and competitive level.

## Methods

### Search Strategy

This study was conducted in accordance with the Preferred Reporting Items for Systematic Reviews and Meta-Analyses (PRISMA) guidelines [51] and registered on the Open Science Framework (https://doi.org/10.17605/OSF.IO/N7BXC). A systematic search was performed to identify original research articles examining the acute effects of short-HIIT. The search was conducted on 2 December, 2025, using the electronic databases PubMed, SPORTDiscus and CINAHL, with no restrictions on language or publication date. Relevant keywords for each search term were identified through pilot searches of titles, abstracts and full texts of previously known articles. The key search terms were grouped and applied within article titles, abstracts and keywords, as outlined in Tables S1–S3 of the Electronic Supplementary Material.

**Fig. 1:**
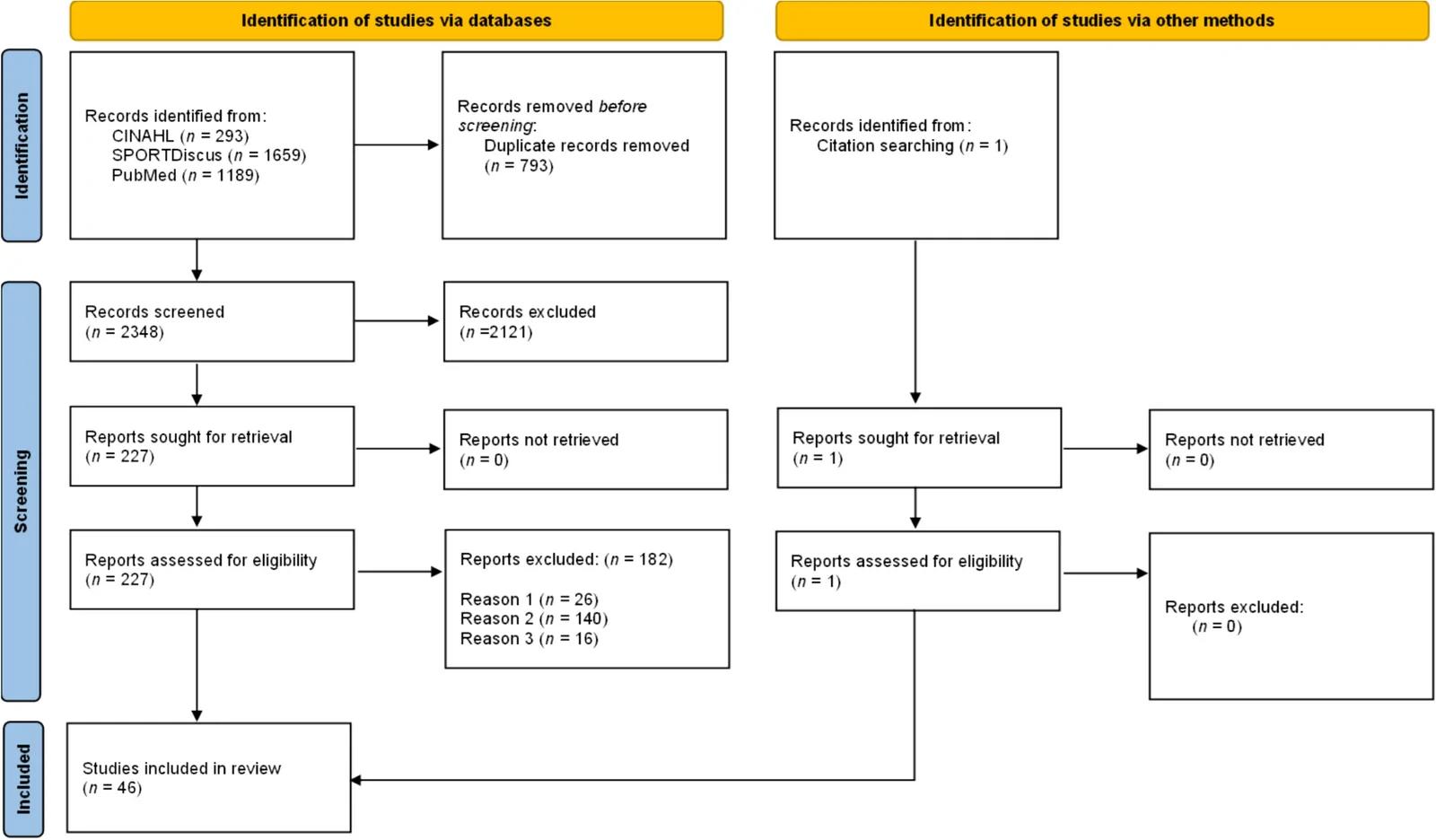
Strategy for the study selection process. Reason 1 = did not meet the population criteria; reason 2 = did not meet the intervention criteria; reason 3 = did not meet the outcome criteria

After the initial literature search, results were exported to EndNote (EndNote 21.2; Clarivate, Philadelphia, PA, USA) and duplicates were removed. The titles and abstracts were then independently screened by two authors (FT, NC), who were not blinded to journal names or manuscript authors. Full-text versions of the remaining articles were accessed to assess their final inclusion or exclusion status. Any disagreements between the two authors were resolved through discussion or consultation with a third author (JW). Additionally, Google Scholar and the reference lists of all eligible articles and relevant reviews [3, 4] were searched to identify further studies. Figure 1 illustrates the study selection process used in this review.

### Inclusion and Exclusion Criteria

We followed the PICOS (Population, Intervention, Comparator, Outcomes, Study design) framework to define and establish the eligibility criteria, which are outlined in Table 1.

**Table 1:** Study inclusion and exclusion criteria

	Inclusion criteria	Exclusion criteria
Population	Healthy, able-bodied, non-injured athletes, aged 16–40 years, of any sex, with a minimum competitive level corresponding to Participant Classification Framework tier 1 (recreationally active) [52]	Special populations (e.g. clinical, patients), people with a physical or mental disability, or people considered to be injured or returning from injury. Athletes under the age of 16 years or over the age of 40 years. Sedentary participants, according to the Participant Classification Framework [52]
Intervention	HIIT was performed as over-ground running on a flat surface or on a treadmill, without any sport skills elements. The HIIT protocol involved repeated (≥ 2) short duration running (10 to < 60 s) with a work to rest ratio of 1:1 HIIT intensity was prescribed via MAS or ASR, as determined by laboratory or field-based incremental exercise tests HIIT must have been performed under normal conditions (e.g. usual nutritional intake, normoxia, absence of ergogenic aids). Placebos permitted	HIIT involved exercise modalities other than running or was performed on a hill. HIIT involved decision making or sport skill elements (e.g. passing, kicking, shooting). The protocol involved maximal sprints, a single effort an alternative work time (< 10 s or ≥ 60 s) or an alternative work-to-rest ratio (e.g. 1:3) HIIT intensity was prescribed via alternative methods (e.g. rating of perceived exertion, heart rate) HIIT was performed under altered or abnormal conditions (e.g. hypoxia, heat stress, ergogenic aids, alternative diet). HIIT was performed in a possibly fatigued or potentiated state (e.g. sports training, maximal fitness assessment, pre-conditioning strategies) occurring within or 24 h before HIIT
Comparator	NA	NA
Outcomes	Studies must have reported an acute outcome measure (outcome measures are presented in Sect. 2.5), which was measured within or immediately following HIIT (0–1 h)	No relevant outcome measures were reported
Study design	Crossover parallel groups (randomised and non-randomised), single group pre-test post-test	Case studies, reviews, opinion pieces
Other	English language, any geographical region	Unpublished studies

*ASR* anaerobic speed reserve, *h* hours, *HIIT* high-intensity interval training, *MAS* maximal aerobic speed, *NA* not applicable, *s* seconds

### Assessment of Reporting Quality and Risk of Bias

To evaluate the reporting quality and risk of bias in the studies included in this review, two authors (FT and NC) independently evaluated the literature using a modified version of the Downs and Black index. This method is valid for assessing the methodological reporting quality of both randomised and non-randomised interventions [53] and has been widely used in systematic reviews and meta-analyses in sport science [18, 54, 55]. The scale includes 14 items, each scored as 0 or 1, with higher total scores (out of 14) reflecting higher quality studies. If clear information was missing to assess an item, it was scored as 0. Any disagreements between the authors were resolved through discussion or by consulting a third author (JW).

### Overall Certainty of Evidence

The overall certainty of evidence for each outcome was evaluated by two authors (FT and NC) using the Grading of Recommendations Assessment, Development, and Evaluation (GRADE) tool [56]. The GRADE domains included inconsistency, heterogeneity, risk of bias, imprecision, indirectness and publication bias, and were rated as ‘not serious’, ‘serious’ and ‘very serious’, following the Cochrane recommendations. Based on these ratings, the overall certainty of evidence for each outcome was classified as ‘very low’, ‘low’, ‘moderate’ or ‘high’, reflecting the level of confidence that the true effect closely aligns with the estimated effect. Any disagreements between the authors were resolved through discussion or consultation with a third author (JW).

### Selection of Outcome Measures and Programming Variables

Outcome measures were selected following a preliminary scoping review of the literature, which identified the most common indicators of physiological and perceptual responses to exercise that also had sufficient data for quantitative synthesis. The selected outcomes were: HR_peak_ and HR_avg_ (b·min^−1^ and percentage of maximal heart rate [%HR_max_]), peak and average $\dot{V}$O_2_ (mL·min^−1^·kg^−1^ and %$\dot{V}$O_2max_), *T* > 90% $\dot{V}$O_2max_, time above 95% of $\dot{V}$O_2max_ (*T* > 95% $\dot{V}$O_2max_), *T* > 90% $\dot{V}$O_2max_/exercise time ratio, *T* > 95% $\dot{V}$O_2max_/exercise time ratio, B[La] and sRPE.

The primary programming variables recorded for a meta-analysis were repetition intensity (i.e. MAS), running modality (i.e. straight-line, 180° shuttle), number of repetitions per set, number of sets per session, repetition duration, and inter-repetition rest modality. Secondary programming variables recorded but not included in our meta-analysis were inter-set rest duration, inter-set rest modality, and the number of 180° changes of direction prescribed during shuttle intervals.

### Extraction of Study Information

The Participant Classification Framework [52] was used to define the training and competitive level of the athletes included in this investigation. Athlete fitness level was determined using graded exercise tests with gas analysis from 29 studies (105 samples) and categorised into the following ranges: 45–50 mL·kg^−1^·min^−1^, 50–55 mL·kg^−1^·min^−1^ and > 60 mL·kg^−1^·min^−1^. To provide insight into the study design (Table S6 of the ESM), studies were classified into three categories: (1) single-group pre-test post-test, an experimental treatment applied to a single group of participants, with dependent variable(s) measured before and after treatment; (2) crossover, two or more experimental conditions were applied to the same participants, with or without a control condition; and (3) parallel groups, two or more experimental conditions were applied to separate participant groups, with or without a control condition. Mean and standard deviation (SD) data were extracted from tables and within the text of the included studies. For data presented in figures, graph digitising software (WebPlotDigitizer, Version 4.3; USA) was used to obtain numerical values. In cases where exercise protocol details relevant to the meta-analysis were missing or clarification was needed, authors were contacted. If no response was received, the corresponding samples were excluded from the meta-analysis.

Heart rate and $\dot{V}$O_2_ data included both work and inter-repetition rest periods but excluded inter-set rest periods. In one study [40], $\dot{V}$O_2_ was reported in L·min^−1^ and was converted to mL·min^−1^·kg^−1^ using the mean body mass of the participants. Sixteen samples from eight studies collected sRPE using Borg’s 6–20 scale, while one study used Borg’s 0–100 scale. To allow for a consistent comparison across studies, these values were converted to CR10^®^ units (deciMax) using the appropriate conversion table [57], with SDs adjusted proportionally to the mean value of each estimate. B[La] values were meta-analysed together regardless of the specific timepoint of measurement (e.g. 0–5 min post-set). When multiple B[La] timepoints were reported, the highest value was selected for our review. For rest modality, ‘passive’ referred to protocols where participants were required to walk to a start line in preparation for the next interval. Protocols prescribing either a fixed number of repetitions or repetitions performed to exhaustion were included in the meta-analysis. To provide additional insight, a sensitivity analysis was conducted including only protocols with a fixed number of repetitions; the results are presented in Table S10 of the ESM.

Only studies that prescribed exercise intensity using MAS or anaerobic speed reserve were included in our review. A maximal, continuous, graded exercise test with gas analysis is considered the gold standard for assessing MAS [58, 59], and was administered in 18 studies. The remaining 26 studies estimated MAS using various incremental field tests, including 20mSRT, 30–15 Intermittent Fitness Test, Carminatti’s Test, University of Montreal Track Test, VAM-EVAL, and Yo-Yo Intermittent Recovery Test Level 1. These field-based tests may either overestimate or underestimate MAS because they represent the final velocity reached during the test rather than lowest velocity that elicits $\dot{V}$O_2max_ (i.e. MAS, also termed v$\dot{V}$O_2max_). Therefore, adjustments to standardise the exercise intensities across studies were required for meaningful comparison. Specifically, in intermittent sport players, the 30–15 Intermittent Fitness Test overestimates MAS by ~ 25% [45, 60, 61] the MSFT underestimates MAS by ~ 15% [62] and the VAM-EVAL underestimates MAS by ~ 11% [63]. These corrections were applied as appropriate (e.g. intensity prescribed at 100% of the final velocity of a 30–15 Intermittent Fitness Test was adjusted to 125% MAS for the meta-analysis). MAS estimated from the Yo-Yo Intermittent Recovery Test, Carminatti’s Test and the University of Montreal Track Test aligns with laboratory-derived MAS (within 0.5 km·h^−1^) [61, 64–69]. While informed by validated studies, we acknowledge that these conversions introduce a layer of estimation that may affect the precision of the intensity moderator. Protocols from studies prescribing intensity using ASR [11, 46, 70] were excluded from the moderator analysis of repetition intensity but included in all other analyses. The methods used by each study to determine MAS and their prescribed intensities are detailed in Table S7 of the ESM.

### Data Analysis

All analyses were conducted using *R* statistical software (Version 4.0.0; R Core Team, 2020). As some of the included studies reported outcomes across multiple subgroups (resulting from repeated measures on the same sample), a multi-level mixed-effects meta-analysis was employed via the *metafor* package [71, 72]. Baseline models were created for each outcome with ten or more estimates, fitting the models using restricted maximum likelihood. These models incorporated only random effects, structured as studies (outer factor) and groups within studies (inner factor; [71]). Individual estimates from groups within studies were treated as the units of analysis, represented by the mean outcome value following HIIT. Standard deviations and sample sizes were used to compute the variance for each estimate. In cases where a study involved repeated measures (i.e. multiple data points for the same group of athletes), dependency was accounted for by replacing variance with the entire ‘V’ matrix; that is, the variance–covariance matrix of the estimates [71]. Block-diagonal covariance matrices were estimated with an assumed correlation of *r* = 0.5 using the *clubSandwich* package [18].

To express uncertainty in the meta-analysed estimates, 90% confidence limits were calculated based on a t-distribution with the denominator degrees of freedom determined by the unique number of ‘group’ levels (i.e. the inner level of the random effects structure). Pooled estimates were also presented with 90% prediction intervals to convey the expected range of true demands of short-HIIT in similar future studies [73]. Between-study and between-group heterogeneity in each meta-analysed estimate was quantified using the SD (sigma [*σ*]; [74]), with 90% CI calculated using the Q-profile method [75].

**Fig. 2:**
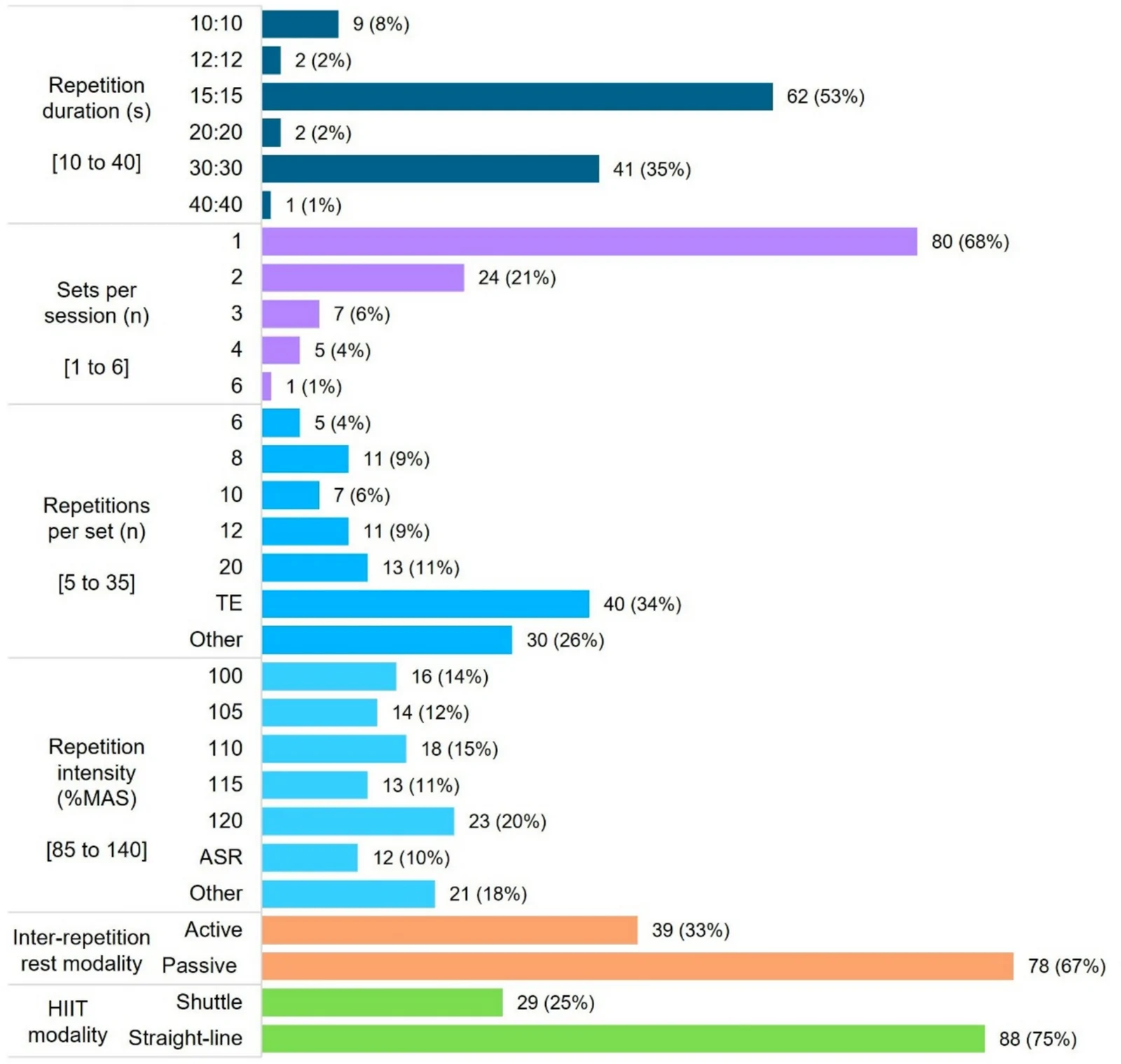
Distribution of short-high-intensity interval training (HIIT) prescriptions across all 117 protocols. Data are given as the total number of protocols represented (percentage), [range]. *ASR* anaerobic speed reserve, *MAS* maximal aerobic speed, *n* number, *s* seconds, *TE* repetitions performed to exhaustion. Note that when protocols that prescribed repetitions to exhaustion were included, 12 repetitions was the most commonly completed number

Meta-regression was used to evaluate the effects of contextual factors on the outcomes, by adding candidate factors as fixed effects to the baseline models, for outcomes with sufficient data (≥ 10 estimates per moderator [56]). The contextual factors included were: competitive level (categorical: recreational, trained or national), fitness level (categorical: $\dot{V}$O_2max_ of 50–55, 55–60 or > 60 mL·kg^−1^·min^−1^), interval intensity (MAS; continuous, linear), running modality (categorical: straight-line or 180° shuttle), number of repetitions per set (continuous, linear), number of sets per session (continuous, linear), duration of each repetition (continuous, linear) and inter-repetition rest modality (categorical: active or passive). To analyse the moderating effects of competitive level and fitness level, factors were re-scaled to set the reference (intercept) effect as the response to the recreational competitive level and $\dot{V}$O_2max_ category of 50–55 mL·kg^−1^·min^−1^, respectively. To analyse the moderating effects of programming variables, factors were re-scaled to the reference (intercept) effect as the response to 1 set of 12 straight-line repetitions, performed at 120% MAS with a 15-s work duration and 15 s of passive rest, representing the most common prescription of each programming variable (Fig. 2). The effects of each moderator were estimated (along with 90% CI and 90% prediction intervals where appropriate), holding all other factors constant. Categorical moderators were compared as the difference between levels (e.g. shuttle vs straight-line sprints, active vs passive rest). Continuous moderators were assessed based on practically relevant training prescriptions, including a 5% increase in MAS, two additional repetitions, one more set and a 15-s longer interval (i.e. 30 s of running with 30 s of rest). The total amount of variance explained by the combination of moderators was given as a pseudo-*R*^2^ value, calculated by subtracting the total (pooled) variance from final models (${\sigma}_{mods}^{2}$) as a fraction of baseline models (${\sigma}_{base}^{2}$) from 1 (1 − [${\sigma}_{mods}^{2}$/${\sigma}_{base}^{2}]$).

For interpreting the moderators, the entire range of the CI was subjectively considered, with a preference for the point estimate [76]. To provide context for practical significance, effects were visually compared against regions of practical significance—empirically or theoretically anchored reference values for each outcome that have real-world relevance in training load monitoring. These thresholds were: 2 b·min^−1^ (~ 1%) in heart rate [77] and 1 au in CR10-scaled sRPE [78]. For outcomes without established practical reference values, including changes in B[La] above the anaerobic threshold- and $\dot{V}$O_2_-related outcomes, the value of a small standardised effect was used. The between-athlete SDs from included estimates were meta-analysed on the log scale, then multiplied by 0.2. When a CI fell predominantly within the region of practical significance, an effect was deemed trivial. When a CI fell predominantly outside the region of practical significance, an effect was deemed substantial. If there was similar coverage of the CI across the trivial region and the substantial region in only one direction (i.e. positive or negative), the effect was deemed compatible with trivial to substantial effects. Finally, when the CI spanned across substantial regions in both directions, including the trivial region, an effect was considered inconclusive.

## Results

### Study Selection

Following the screening process (Fig. 1), data from 139 samples nested within 46 publications were included in our investigation. In total, there were 681 athlete inclusions and 117 short-HIIT protocols.

### Study Characteristics

The mean (± SD) athlete age across all studies was 22.9 ± 4.9 years (range: 16–39 years). Twenty-five studies provided the $\dot{V}$O_2max_ of their athletes, which collectively was 58.0 ± 6.0 mL·min^−1^·kg^−1^ (range: 44.2–71.1 mL·min^−1^·kg^−1^). The most common study design was single-group crossover (*n* = 35 studies, 76%). Endurance running and soccer were the most investigated sports, represented in 12 (26%) and 10 (22%) studies, respectively. One (2%) study involved international/elite-level athletes, 13 (28%) studies involved national-level athletes, 23 (50%) studies involved trained-level athletes, and 9 (20%) studies involved recreational-level athletes. There were 63 (9%) female athletes within six studies. A summary of the athlete and study characteristics is provided in Table S6 of the ESM.

### Outcomes for the Assessment of Reporting Quality and Risk of Bias

Table S4 of the ESM summarises the outcomes of the modified Downs and Black scale for the assessment of reporting quality and risk of bias. Results ranged from 7 to 13, with a mean score of 10.4 ± 1.0. No studies satisfied item 12 (participants prepared to participate representative of the entire population) and only one study satisfied item 15 (blinding of outcome measures). Additionally, an absence of clear information to assess whether participants were recruited over the same period resulted in all studies being scored 0 for item 22.

### Outcomes for the Overall Certainty of Evidence

The GRADE tool for assessing the overall certainty of evidence is presented in Table S5 of the ESM. The risk of bias for sRPE was rated as serious as studies did not report whether participant responses were concealed, leading to a one-level downgrade in evidence certainty. The certainty of evidence was also downgraded to moderate for *T* > 90% $\dot{V}$O_2max_, *T* > 95% $\dot{V}$O_2max_ and $\dot{V}$O_2peak_, primarily owing to smaller sample sizes and greater variation within and between studies, resulting in wide confidence intervals (CIs) and prediction intervals. For all other outcomes, the risk of bias was rated as not serious, as most studies employed a crossover design, which effectively addressed order effects.

### Study Outcomes

A summary of the training protocols and study outcomes from the included publications is provided in Table S7 of the ESM. Across all protocols, the most common programming characteristics were straight-line running efforts (*n* = 88, 75%), 15:15 s work-to-rest durations (*n* = 62, 53%), passive inter-repetition recovery (*n* = 78, 67%), single-set designs (*n* = 80, 68%), repetitions performed to exhaustion (*n* = 40, 34%), and an intensity prescribed at 120% MAS (*n* = 23, 20%). Among protocols that incorporated active inter-repetition recovery, the most frequent prescription was running at 50% MAS (19 of 39 protocols). For multi-set protocols, the most common inter-set rest duration was 5 min (9 of 37 protocols), and passive rest was the most frequently used inter-set recovery modality (27 of 37 protocols). One study (7 protocols) did not report inter-set rest details. For shuttle-based intervals, the number of prescribed 180° changes of on ranged from 1 to 3. The distribution of short-HIIT prescription across all protocols is presented in Fig. 2.

### Meta-analysed Acute Demands of Short-Bout High-Intensity Interval Training (Short-HIIT)

The acute physiological and perceptual demands of short-HIIT are presented in Table 2. The 90% CI and prediction interval are also presented for each estimate, as well as the between-sample and between-study variability (*σ*).

**Table 2:** Meta-analysed acute physiological and perceptual demands of short-bout high-intensity interval training

Outcome measure		Number of …		Pooled effect			Variation (*σ*; 90% CI) between …	
		Studies	Samples	Estimate	90% CI	90% PI	Studies (*σ*_1_)	Samples (*σ*_2_)
B[La]	mmol·L^−1^	26	64	8.3	7.3–9.3	2.8–13.8	2.5 (1.7–3.5)	1.9 (1.6–2.4)
HR_avg_	b·min^−1^	13	24	169	165–174	152–187	9 (7–14)	2 (1–5)
	% HR_max_	13	23	88	87–90	82–95	3 (2–5)	2 (1–3)
HR_peak_	b·min^−1^	17	33	184	180–188	168–200	7 (4–11)	6 (4–8)
	%HR_max_	7	20	94	92–96	88 to 100	1 (0–4)	3 (2–4)
$\dot{V}$O_2avg_	mL·kg^−1^·min^−1^	8	17	46	43–49	36–55	4 (2–7)	3 (2–4)
	% $\dot{V}$O_2max_	6	9	78	69–86	55–100	10 (6–19)	3 (1–8)
$\dot{V}$O_2peak_	mL·kg^−1^·min^−1^	11	19	55	51–60	39–72	9 (6–13)	0 (0–2)
	%$\dot{V}$O_2max_	6	8	93	87–100	76–111	7 (0–15)	3 (0–11)
*T* > 90% $\dot{V}$O_2max_	s	16	30	259	202–315	17–500	103 (47–163)	85 (52–133)
*T* > 95% $\dot{V}$O_2max_	s	10	18	125	78–173	− 28 to 278	63 (0–125)	48 (29–84)
*T*_90_/ET		15	29	0.34	0.25–0.42	0.01–0.67	0.16 (0.10–0.22)	0.08 (0.04–0.14)
*T*_95_/ET		10	18	0.15	0.08–0.22	− 0.07 to 0.37	0.09 (0.00–0.17)	0.07 (0.04–0.13)
sRPE	(CR10^®^ units)	22	73	7.1	6.6–7.5	4.6–9.6	1.1 (0.7–1.5)	1.0 (0.8–1.1)

*B[La]* end-set blood lactate concentration, *CI* confidence interval, *PI* prediction interval, *HR*_*avg*_ average heart rate, *%HR*_*max*_ percentage of maximal heart rate, *HR*_*peak*_ peak heart rate, $\dot{V}$*O*_*2avg*_ average oxygen consumption, $\dot{V}$*O*_*2peak*_ peak oxygen consumption, *%*$\dot{V}$*O*_*2max*_ percentage of maximal oxygen consumption, * T* > *90% *$\dot{V}$*O*_*2max*_ time above 90% of maximal oxygen consumption, * T* > *95% *$\dot{V}$*O*_*2max*_ time above 95% of maximal oxygen consumption, * T*_90_*/ET* time above 90% of maximal oxygen consumption/exercise time ratio, * T*_95_*/ET* time above 95% of maximal oxygen consumption/exercise time ratio, *s* seconds, *sRPE* session ratings of perceived exertion

### Moderating Effects of Programming Variables on the Acute Demands of Short-HIIT

**Fig. 3:**
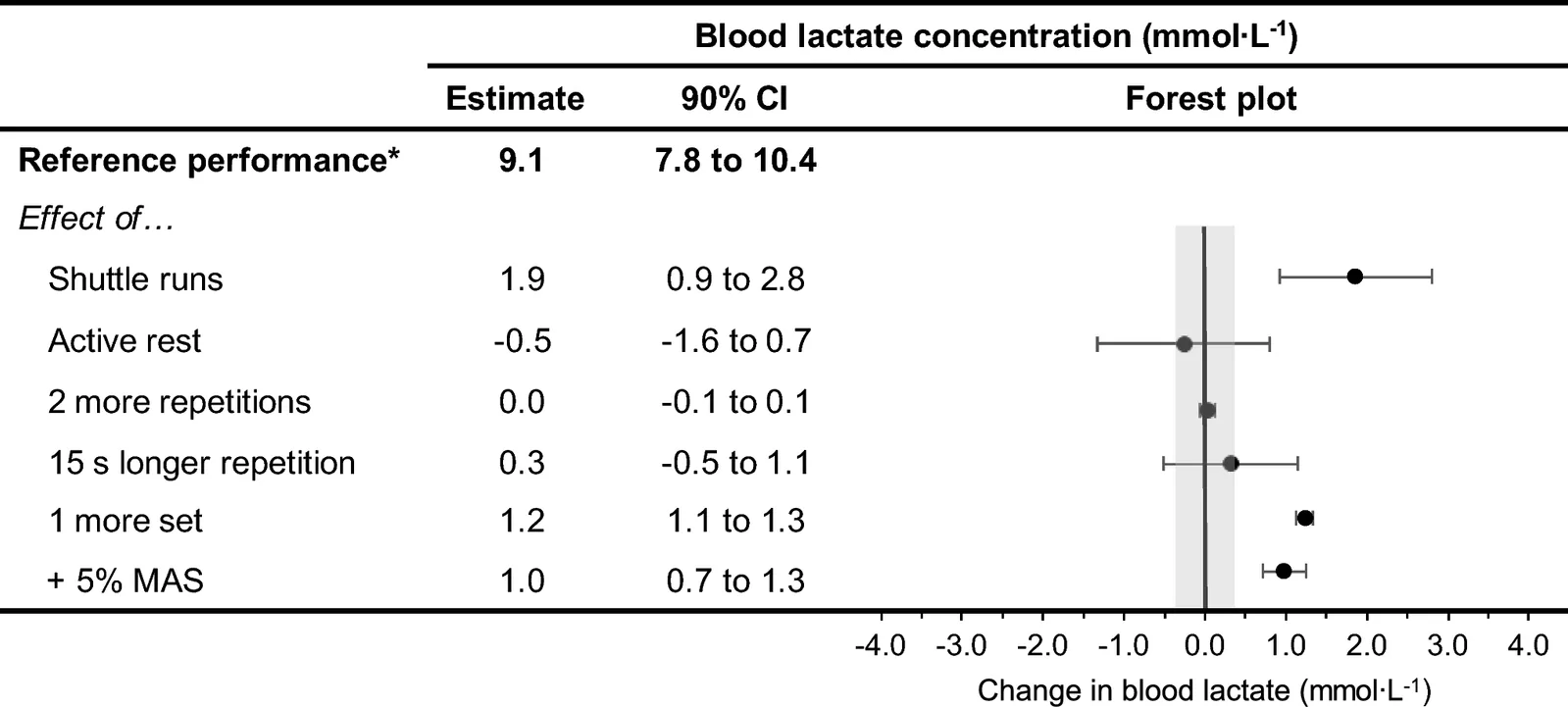
Moderating effects of programming variables on change in end-set blood lactate concentration during short-bout high-intensity interval training. *CI* confidence interval, *MAS* maximal aerobic speed, * 1 set of 12 straight-line repetitions, performed at 120% MAS for a 15-s work duration with 15 s of passive rest. Note: the *area outside the shaded zone* represents the region of practical significance

**Fig. 4:**
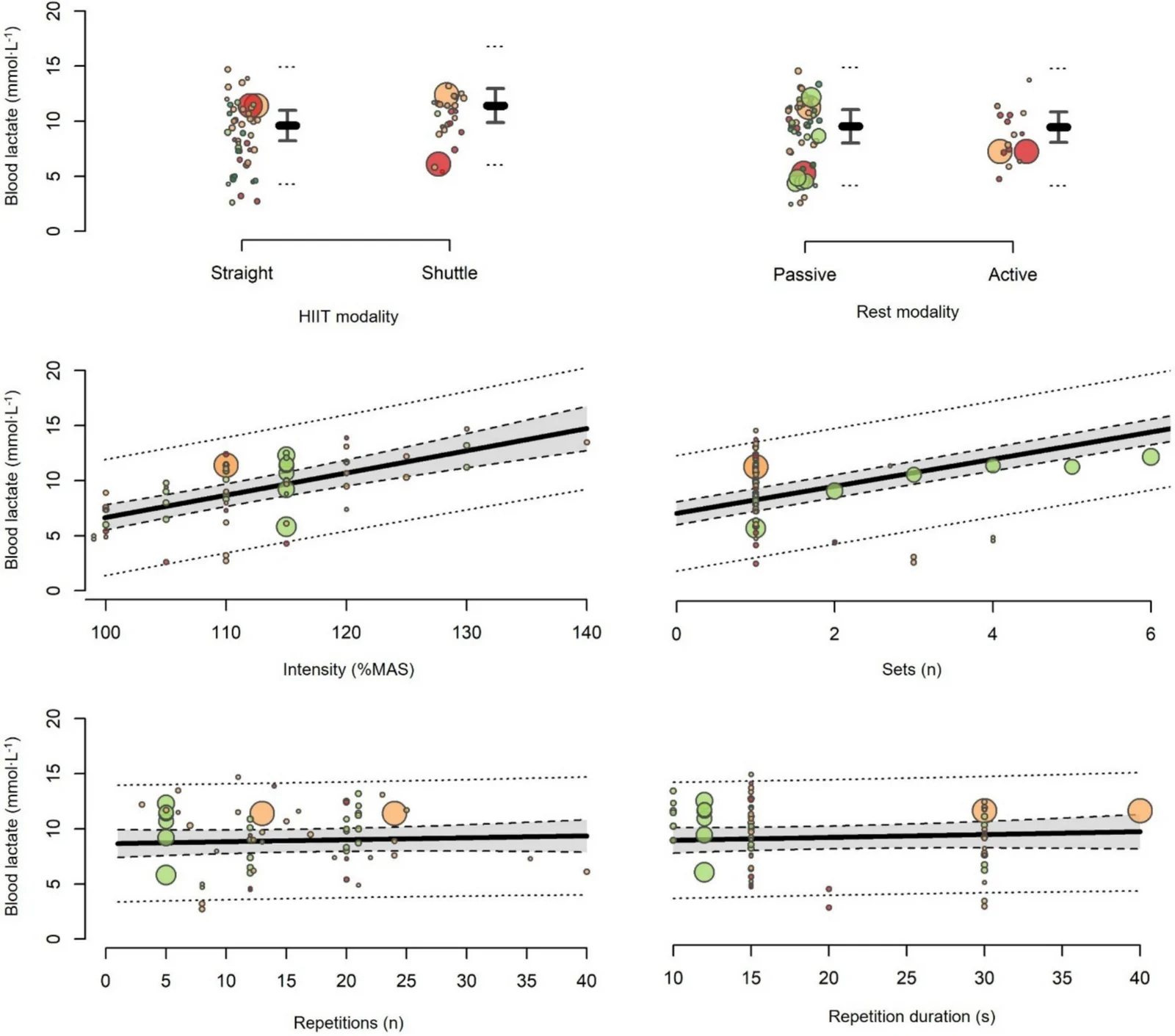
Moderating effects of programming variables on end-set blood lactate concentration during short-bout high-intensity interval training (HIIT). *Red* = recreational athletes; *orange* = trained athletes; *light green* = national athletes; *larger circle* = larger study sample; *grey zone* = 90% confidence interval. *MAS* maximal aerobic speed, *s* seconds

**Fig. 5:**
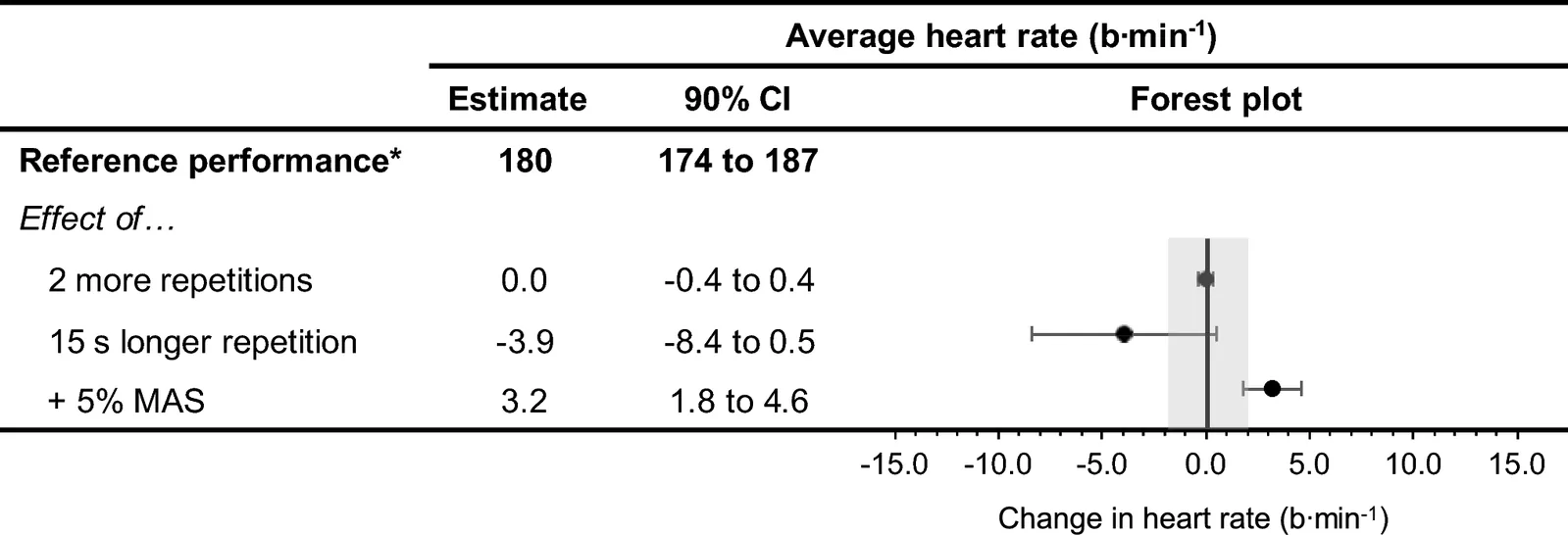
Moderating effects of programming variables on the change in average heart rate during short-bout high-intensity interval training. *CI* confidence interval, *MAS* maximal aerobic speed, *s* seconds, * 1 set of 12 straight-line repetitions, performed at 120% MAS for a 15-s work duration with 15 s of passive rest. Note: the *area outside the shaded zone* represents the region of practical significance

**Fig. 6:**
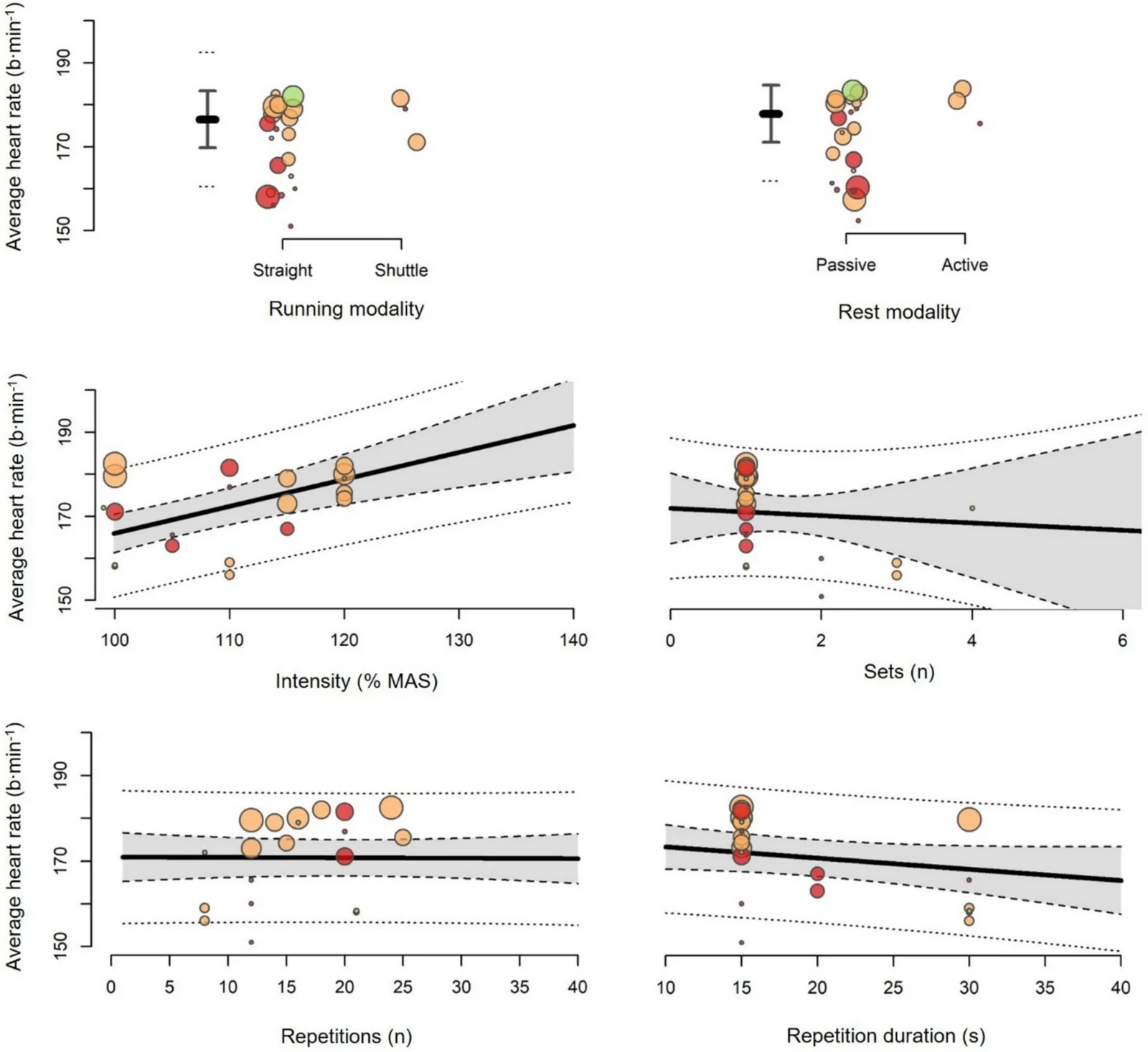
Moderating effects of programming variables on average heart rate during short-bout high-intensity interval training. *Red* = recreational athletes; *orange* = trained athletes; *light green* = national athletes; *larger circle* = larger study sample; *grey zone* = 90% confidence interval. *MAS* maximal aerobic speed, *s* seconds

**Fig. 7:**
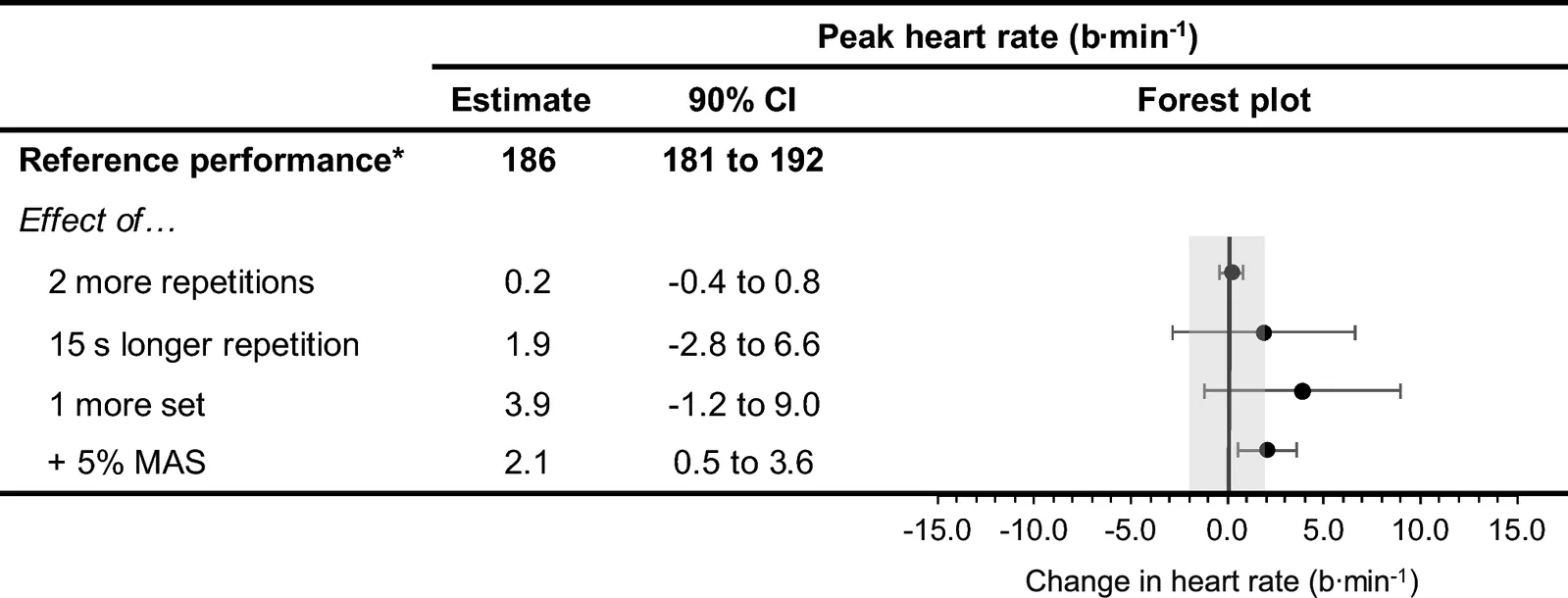
Moderating effects of programming variables on the change in peak heart rate during short-bout high-intensity interval training. *CI* confidence interval, *MAS* maximal aerobic speed, *s* seconds, * 1 set of 12 straight-line repetitions, performed at 120% MAS for a 15-s work duration with 15 s of passive rest. Note: the *area outside the shaded zone* represents the region of practical significance

**Fig. 8:**
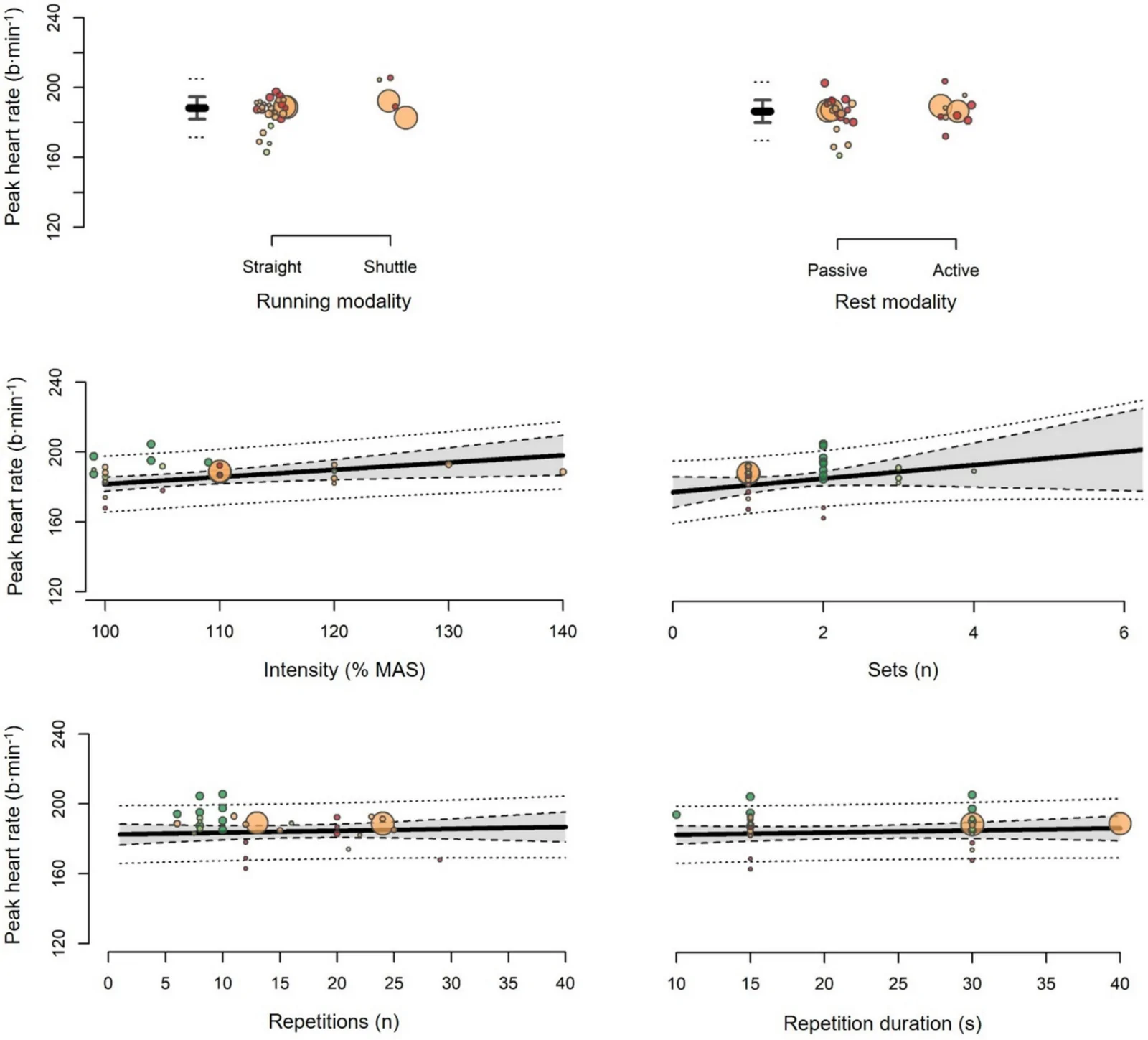
Moderating effects of programming variables on the peak heart rate during short-bout high-intensity interval training. *MAS* maximal aerobic speed, *s* seconds. *Red* = recreational athletes; *orange* = trained athletes; *light green* = national athletes; *dark green* = international athletes; *larger circle* = larger study sample; *grey zone* = 90% confidence interval

**Fig. 9:**
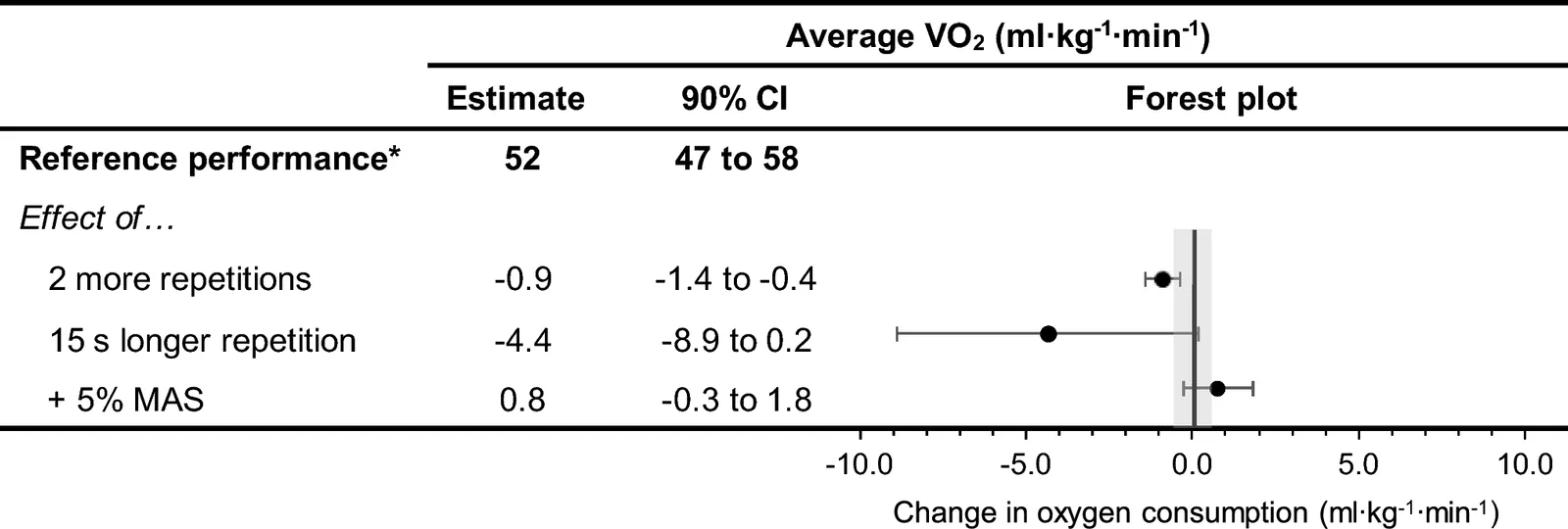
Moderating effects of programming variables on the change in average oxygen consumption ($\dot{V}$O_2_) during short-bout high-intensity interval training. *CI* confidence interval, *MAS* maximal aerobic speed, *s* seconds, * 1 set of 12 straight-line repetitions, performed at 120% MAS for a 15-s work duration with 15 s of passive rest;. Note: the *area outside the shaded zone* represents the region of practical significance

**Fig. 10:**
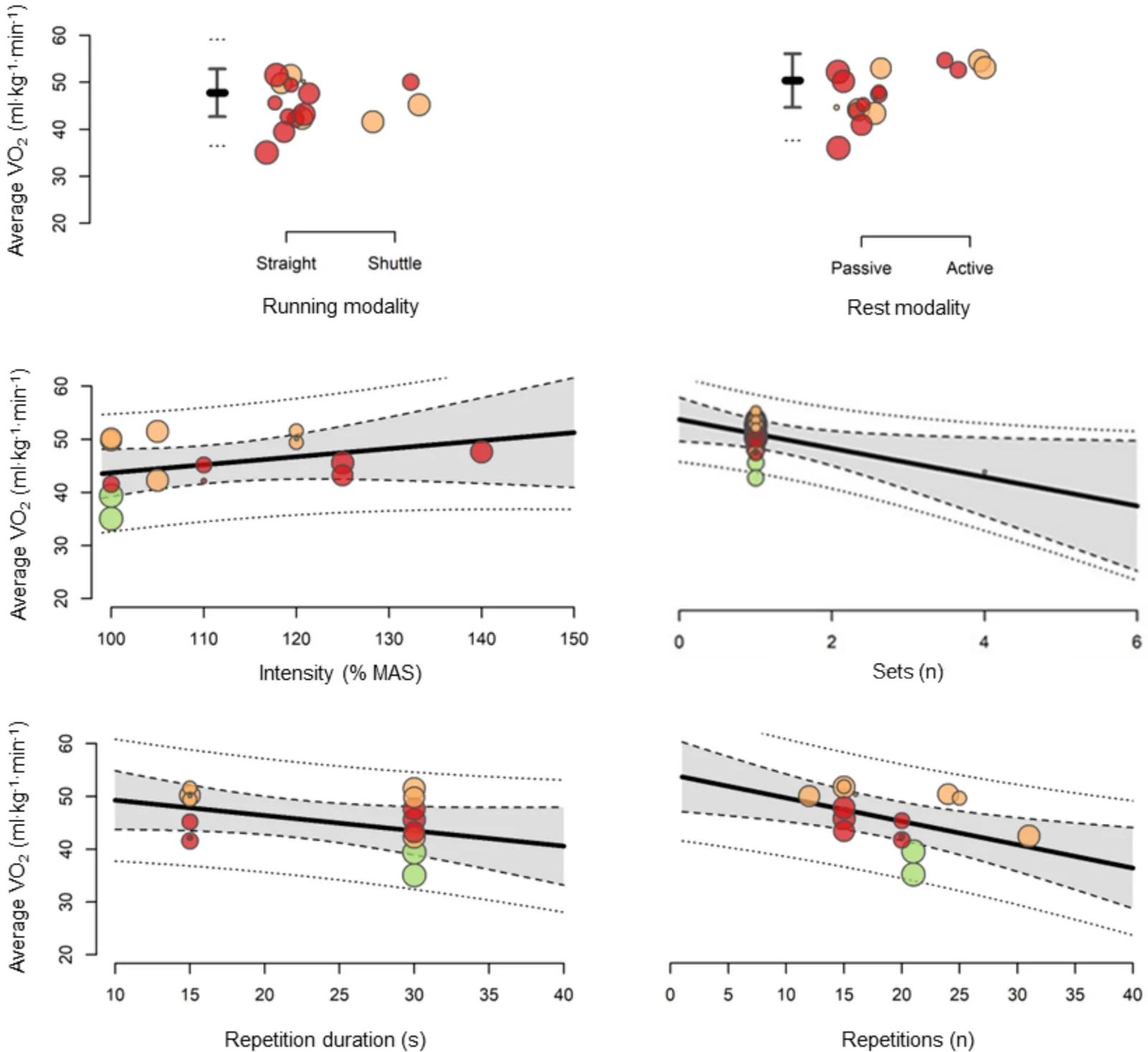
Moderating effects of programming variables on average oxygen consumption ($\dot{V}$O_2_) during short-bout high-intensity interval training. *Red* = recreational athletes; *orange* = trained athletes; *light green* = national athletes; *larger circle* = larger study sample; *grey zone* = 90% confidence interval (CI). *MAS* maximal aerobic speed, *s* seconds

**Fig. 11:**
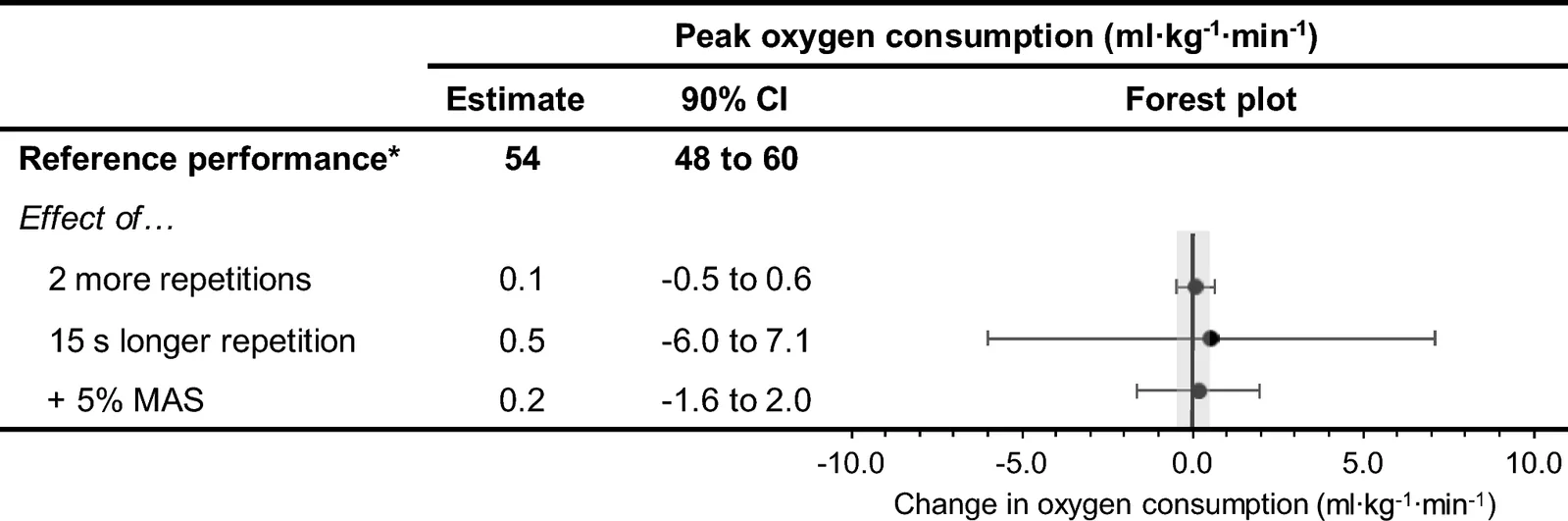
Moderating effects of programming variables on the change in peak oxygen consumption during short-bout high-intensity interval training. *CI* confidence interval, *MAS* maximal aerobic speed, *s* seconds, * 1 set of 12 straight-line repetitions, performed at 120% MAS for a 15 s work duration with 15 s of passive rest. Note: the *area outside the shaded zone* represents the region of practical significance

**Fig. 12:**
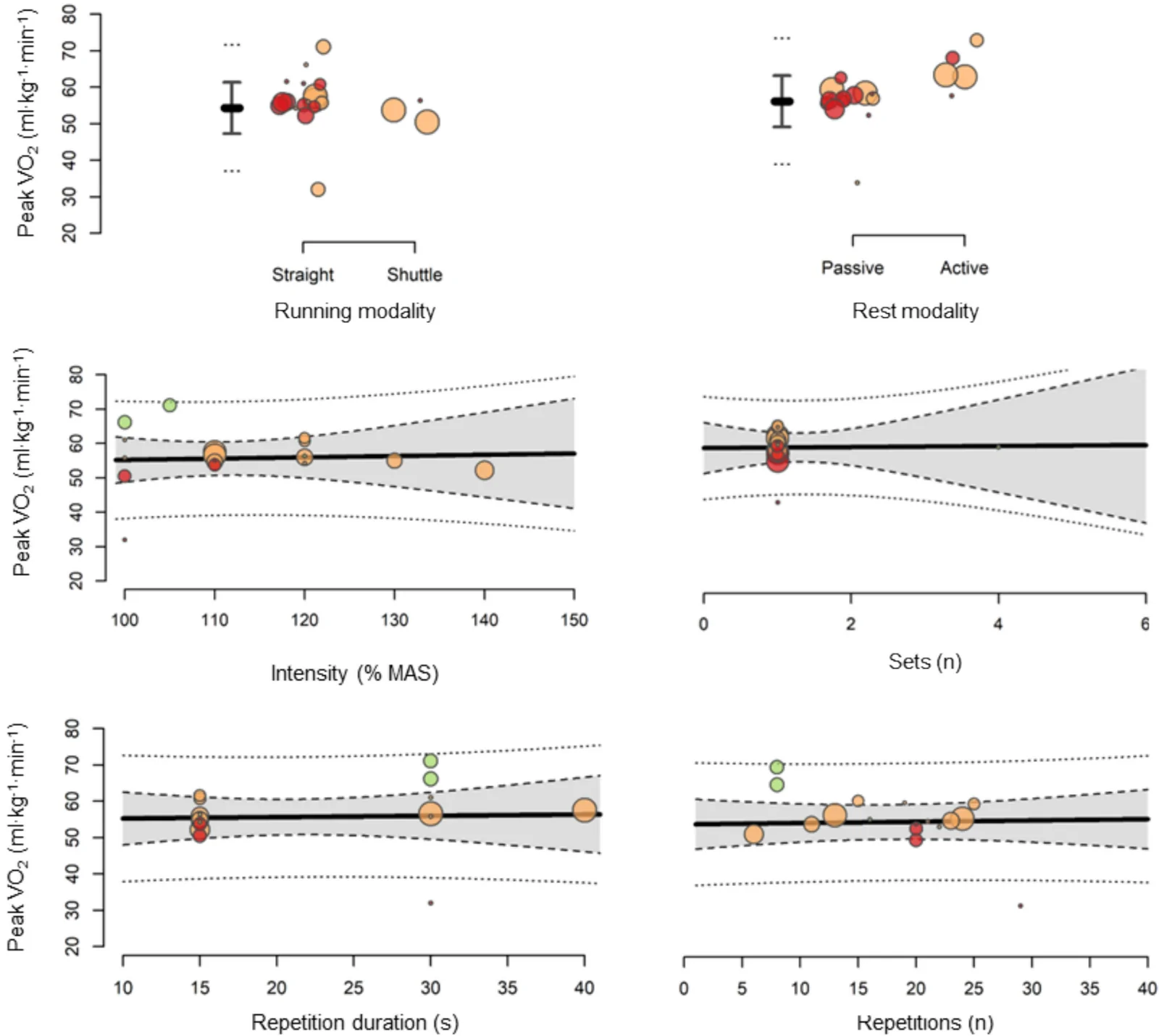
Moderating effects of programming variables on peak oxygen consumption ($\dot{V}$O_2_) during short-bout high-intensity interval training. *Red* = recreational athletes; *orange* = trained athletes; *light green* = national athletes; *larger circle* = larger study sample; *grey zone* = 90% confidence interval. *MAS* maximal aerobic speed, *s* seconds

**Fig. 13:**
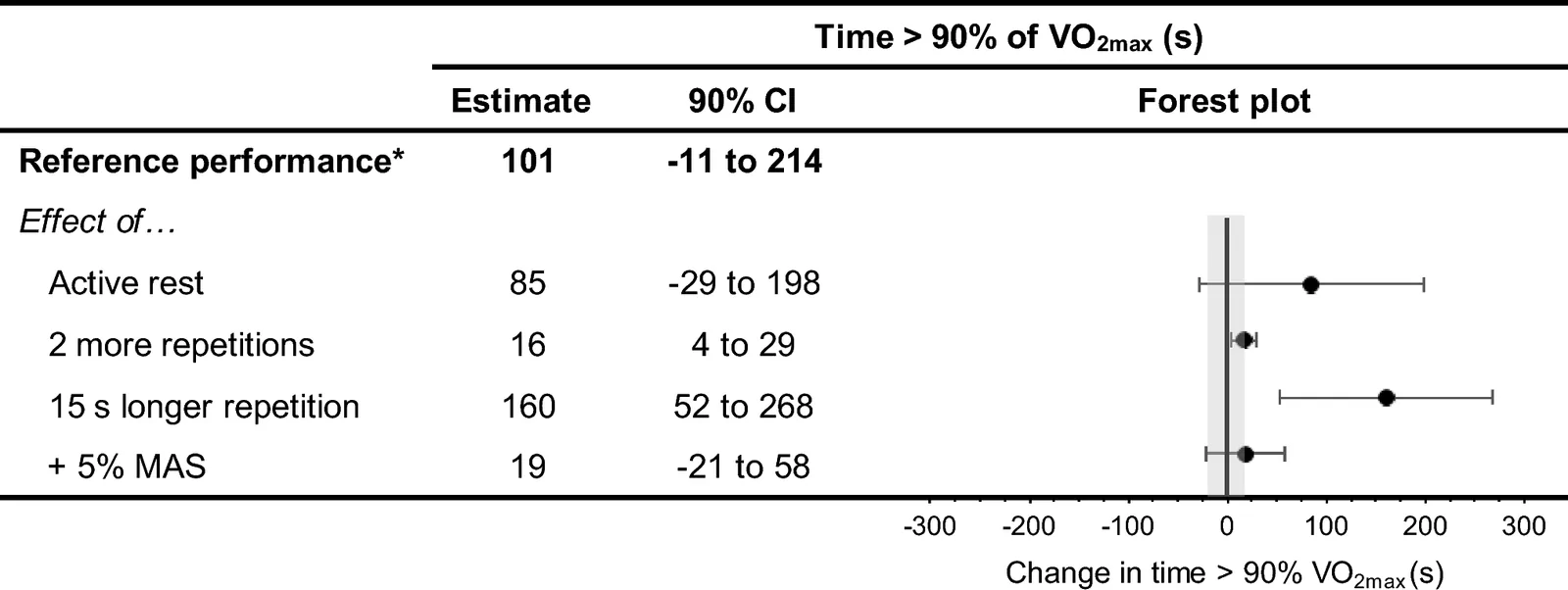
Moderating effects of programming variables on the change in time above 90% of maximal oxygen consumption ($\dot{V}$O_2max_) during short-bout high-intensity interval training. *CI* confidence interval, *MAS* maximal aerobic speed, *s* seconds, * 1 set of 12 straight-line repetitions, performed at 120% MAS for a 15-s work duration with 15 s of passive rest. Note: the *area outside the shaded zone* represents the region of practical significance

**Fig. 14:**
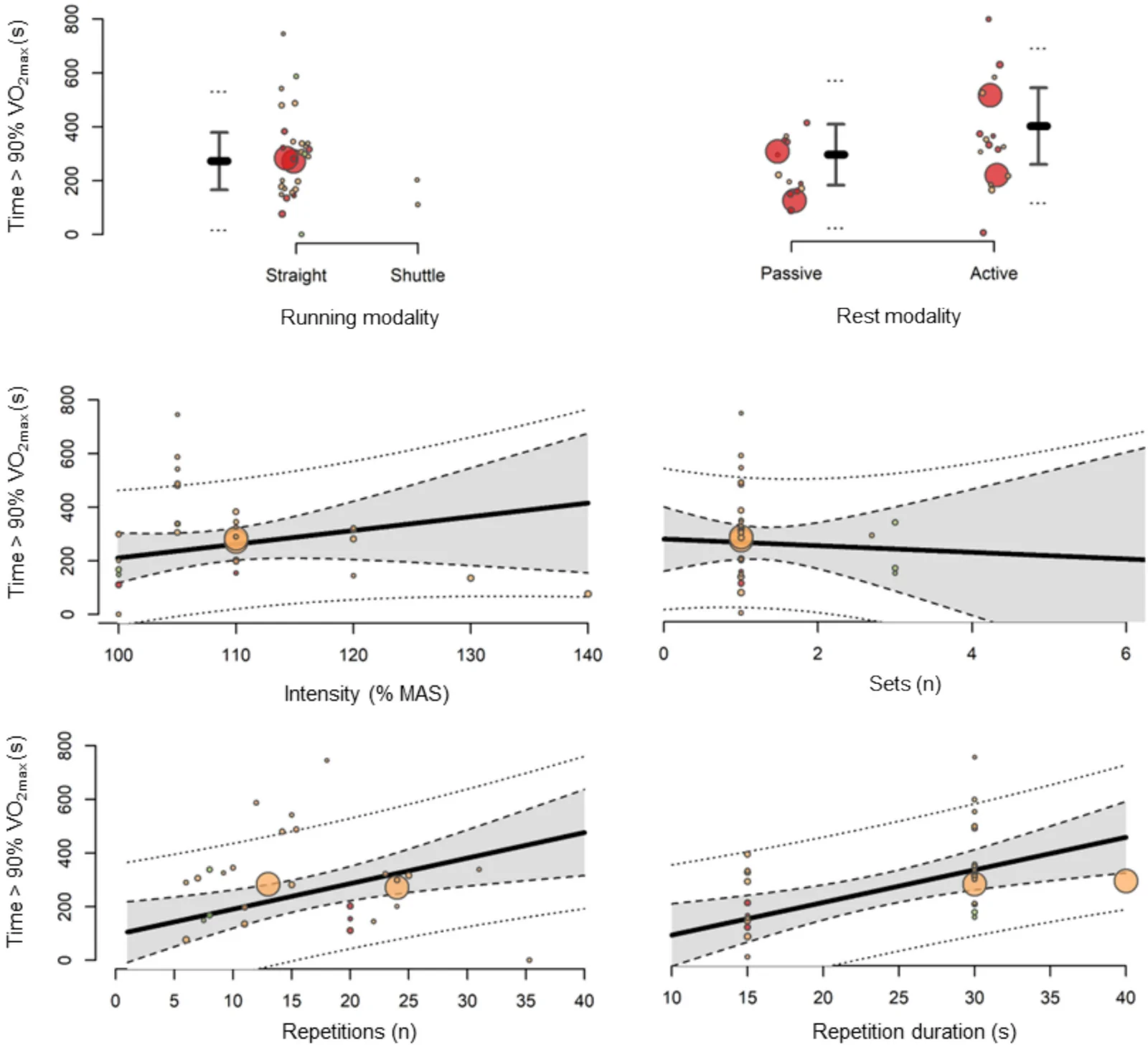
Moderating effects of programming variables on time above 90% of maximal oxygen consumption ($\dot{V}$O_2max_) during short-bout high-intensity interval training. *Red* = recreational athletes; *orange* = trained athletes; *light green* = national athletes; *larger circle* = larger study sample; *grey zone* = 90% confidence interval. *MAS* maximal aerobic speed, *s* seconds

**Fig. 15:**
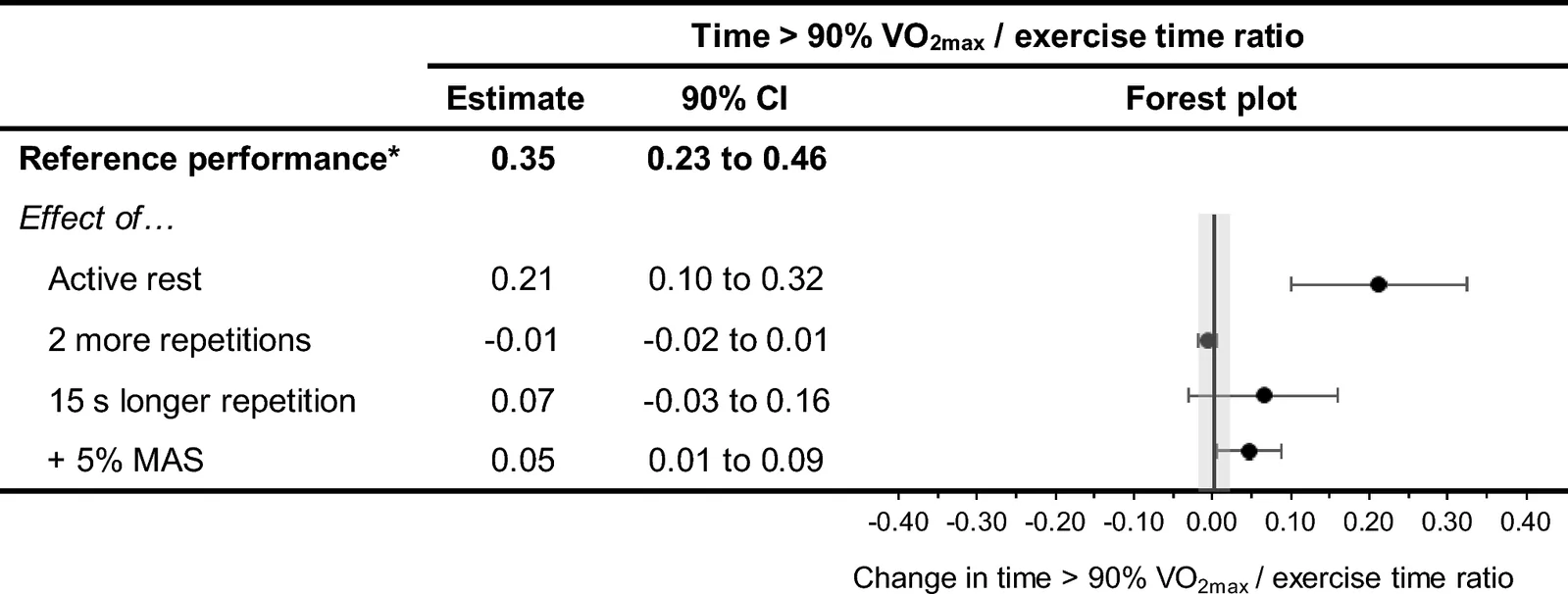
Moderating effects of programming variables on the change in time above 90% of maximal oxygen consumption ($\dot{V}$O_2max_)/exercise time ratio during short-bout high-intensity interval training. *CI* confidence interval, *MAS* maximal aerobic speed, *s* seconds, * 1 set of 12 straight-line repetitions, performed at 120% MAS for a 15-s work duration with 15 s of passive rest. Note: the *area outside the shaded zone* represents the region of practical significance

**Fig. 16:**
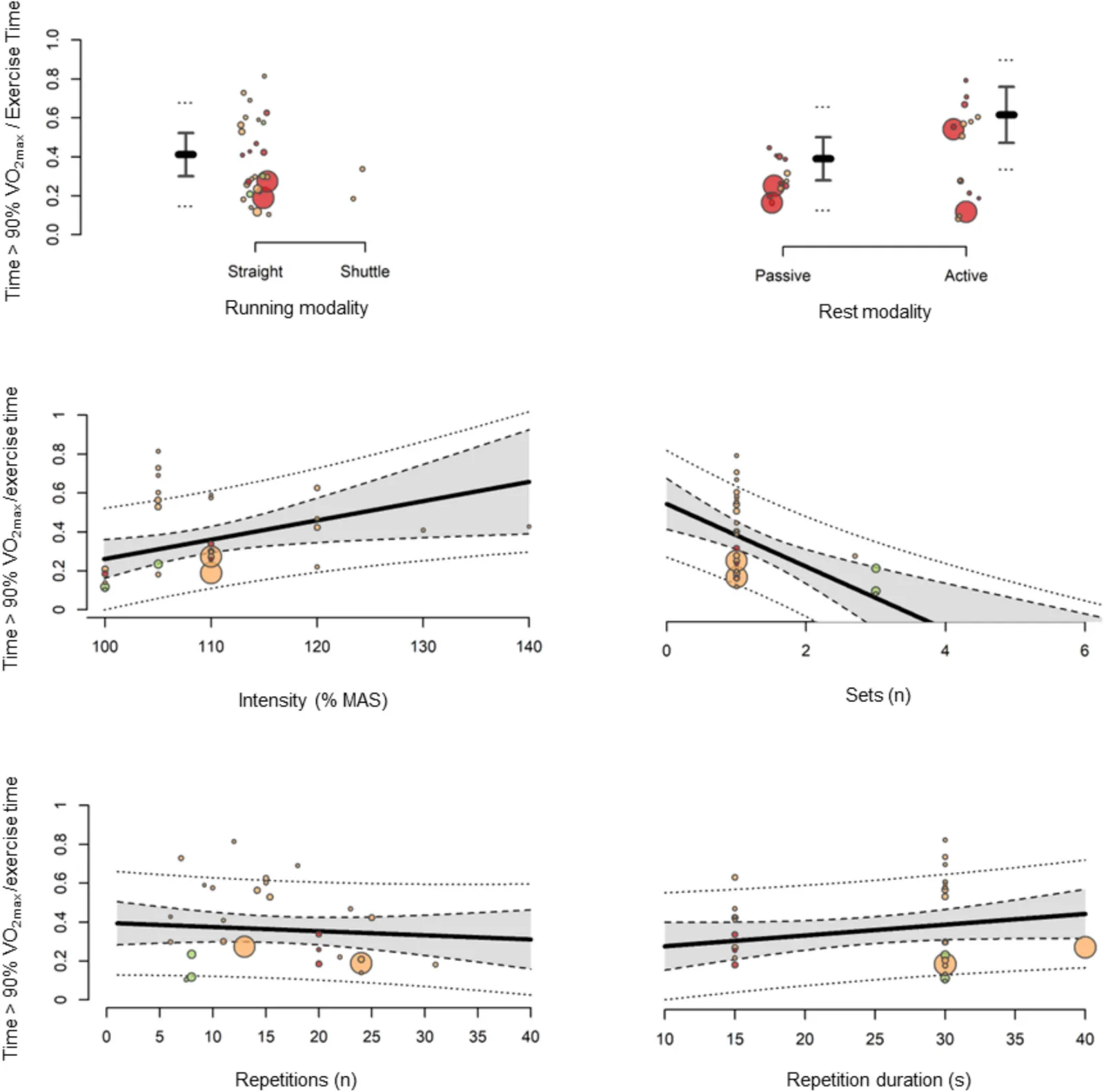
Moderating effects of programming variables on time above 90% of the maximal oxygen consumption ($\dot{V}$O_2max_)/exercise time ratio during short-bout high-intensity interval training. *Red* = recreational athletes; *orange* = trained athletes; *light green* = national athletes; *larger circle* = larger study sample; *grey zone* = 90% confidence interval. *MAS* maximal aerobic speed, *s* seconds

**Fig. 17:**
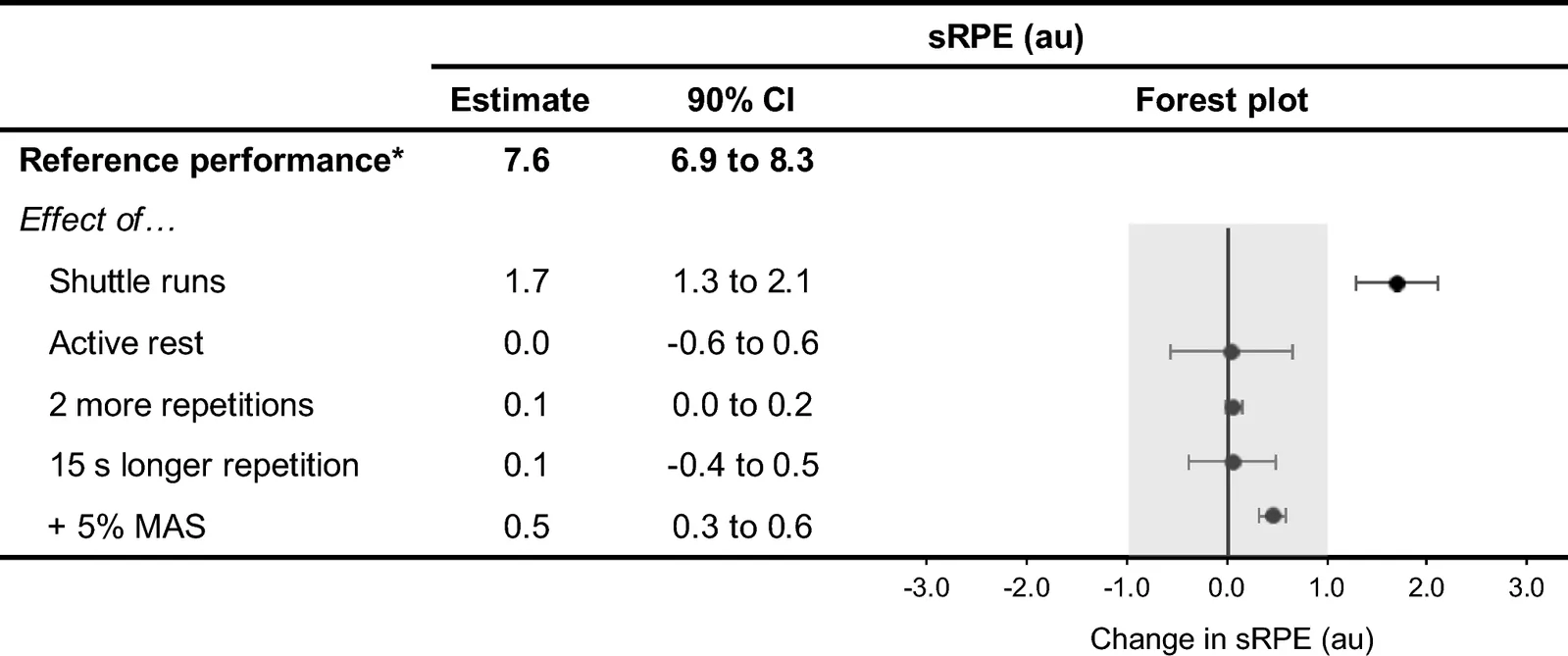
Moderating effects of programming variables on the change in session ratings of perceived exertion (CR10^®^) during short-bout high-intensity interval training. *au* arbitrary units, *CI* confidence interval, *MAS* maximal aerobic speed, *s* seconds, *sRPE* session ratings of perceived exertion, * 1 set of 12 straight-line repetitions, performed at 120% MAS for a 15-s work duration with 15 s of passive rest. Note: *the area outside the shaded zone* represents the region of practical significance

**Fig. 18:**
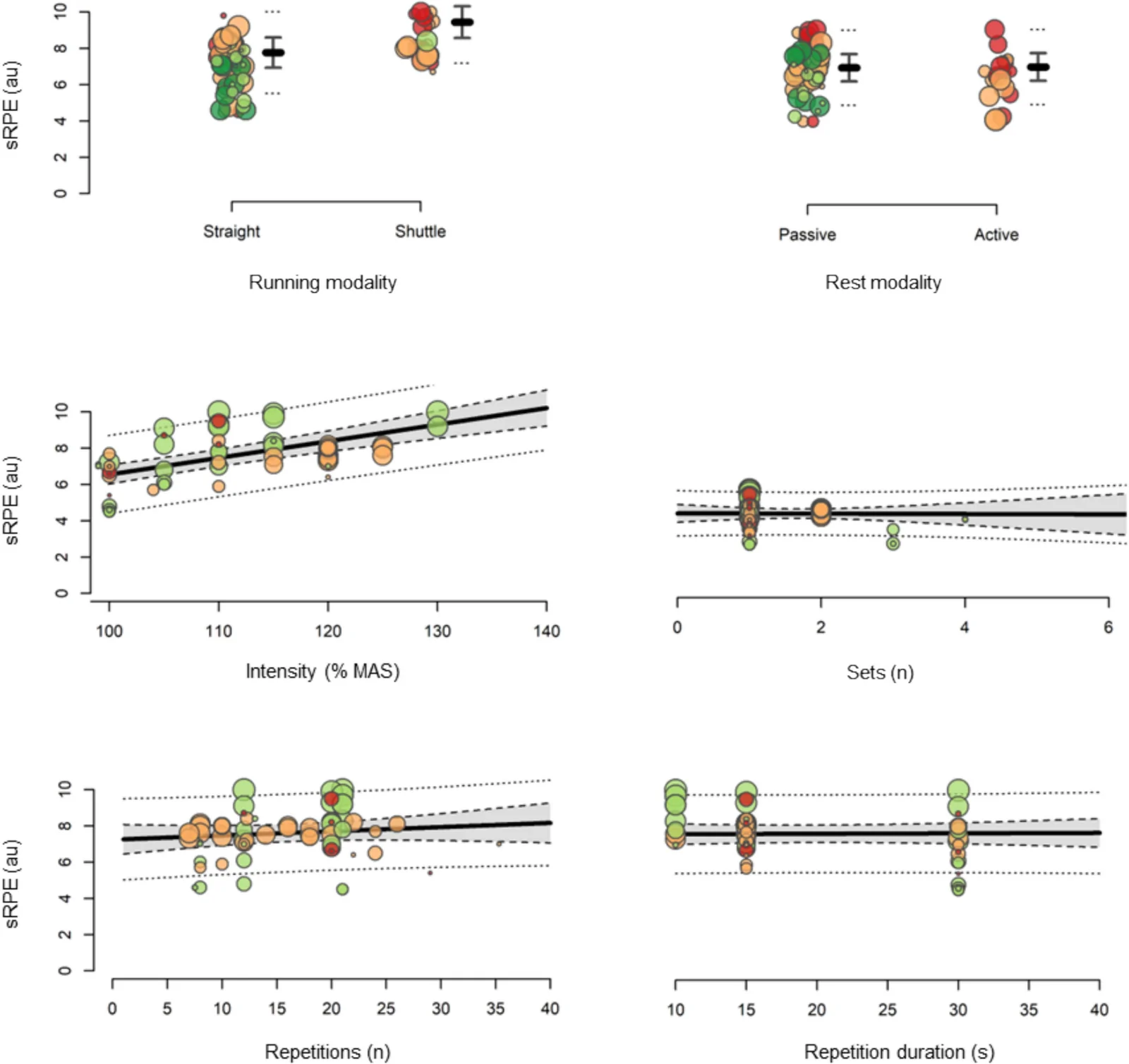
Moderating effects of programming variables on session ratings of perceived exertion during short-bout high-intensity interval training. *Red* = recreational athletes; *orange* = trained athletes; *light green* = national athletes; *dark green* = international athletes; *larger circle* = larger study sample; *grey zone* = 90% confidence interval. *au* arbitrary units, *MAS* maximal aerobic speed, *sRPE* session ratings of perceived exertion

The moderating effects of programming variables on B[La], heart rate, $\dot{V}$O_2_, *T* > 90% $\dot{V}$O_2max_, *T* > 90% $\dot{V}$O_2max_/exercise time ratio, and sRPE are presented in Figs. 3, 4, 5, 6, 7, 8, 9, 10, 11, 12, 13, 14, 15, 16, 17 and 18. The moderating effects of programming variables on *T* > 95% $\dot{V}$O_2max_ and the *T* > 95% $\dot{V}$O_2max_/exercise time ratio are presented in Figs. S1–S4 of the ESM. All effects were evaluated as the change in each outcome measure when compared to a reference protocol of 1 set of 12 straight-line repetitions, performed at 120% MAS with a 15-s work duration and 15 s of passive rest. It is important to note that this protocol represents the most studied programming variables, which enabled meta-analysis; it does not represent an “ideal” protocol. Outcomes with fewer than ten samples per moderator were not evaluated, but data are shown for transparency and to support interpretation in Figs. 4, 6, 8, 10, 12, 14, 16, and 18.

#### Shuttle Runs

Shuttle runs were associated with a substantial increase in B[La] (Figs. 3 and 4) and sRPE (Figs. 17 and 18). All other outcomes were not evaluated because of an insufficient number of samples for shuttle run protocols.

#### Active Rest

Active inter-repetition rest was associated with a substantial increase in the *T* > 90% $\dot{V}$O_2max_/exercise time ratio (Figs. 15 and 16), whereas the effect on sRPE was trivial (Figs. 17 and 18). The effects on B[La] (Figs. 3 and 4) and *T* > 90% $\dot{V}$O_2max_ (Figs. 13 and 14) were inconclusive (i.e. a CI spanning across substantial regions in both positive and negative directions, including the trivial region). The effects on HR_avg_, HR_peak_, peak $\dot{V}$O_2_, average $\dot{V}$O_2_, *T* > 95% $\dot{V}$O_2max_ and the *T* > 95% $\dot{V}$O_2max_/exercise time ratio were not evaluated because of an insufficient number of samples for active rest protocols.

#### Performing Two More Repetitions Per Set

Performing two more repetitions per set was associated with a trivial to substantial decrease in average $\dot{V}$O_2_ (Figs. 9 and 10), whereas the effects on B[La] (Figs. 3 and 4), HR_avg_ (Figs. 5 and 6), HR_peak_ (Figs. 7 and 8), *T* > 90% $\dot{V}$O_2max_ (Figs. 13 and 14), *T* > 95% $\dot{V}$O_2max_ (Figs. S1 and S2 of the ESM), *T* > 90% $\dot{V}$O_2max_/exercise time ratio (Figs. 15 and 16)_,_ *T* > 95% $\dot{V}$O_2max_/exercise time ratio (Figs. S3 and S4 of the ESM) and sRPE (Figs. 17 and 18) were trivial (Figs. 3, 4, 5, 6, 7, 8, 9, 10, 11, 12, 13, 14, 15, 16, 17 and 18). The effect on peak $\dot{V}$O_2_ was inconclusive (Figs. 11 and 12).

#### Performing a 15-Second Longer Repetition

Extending the repetition duration by 15 s (i.e. 30 s work with 30 s recovery) was associated with a substantial increase in *T* > 90% $\dot{V}$O_2max_ (Figs. 13 and 14) but was also compatible with a trivial to substantial decrease in HR_avg_ (Figs. 5 and 6). The effect on sRPE was trivial (Figs. 17 and 18), while the effects on all other outcomes were inconclusive.

#### Performing One More Set Per Session

Performing one more set per session was associated with a substantial increase in B[La] (Figs. 3 and 4), while the effect on HR_peak_ was compatible with a trivial to substantial increase (Figs. 7 and 8). The effects on all other outcomes were not evaluated because of an insufficient number of samples for protocols involving more than one set.

#### A 5% Increase in Maximal Aerobic Speed (MAS)

A 5% increase in MAS was associated with a substantial increase in B[La] (Figs. 3 and 4), HR_avg_ (Figs. 5 and 6) and the *T* > 90% $\dot{V}$O_2max_/exercise time ratio (Figs. 15 and 16), but a substantial decrease in *T* > 95% $\dot{V}$O_2max_ (Figs. S1 and S2 of the ESM). Additionally, a 5% increase in MAS was compatible with both a trivial to substantial increase in HR_peak_ (Figs. 7 and 8) and average $\dot{V}$O_2_ (Figs. 9 and 10), while the effects on sRPE (Figs. 17 and 18) were trivial. The effects on peak $\dot{V}$O_2_ (Figs. 11 and 12), *T* > 90% $\dot{V}$O_2max_ (Figs. 13 and 14), and the *T* > 95% $\dot{V}$O_2max_/exercise time ratio (Figs. S3 and S4 of the ESM) were inconclusive.

### Moderating Effects of the Athlete Competitive Level on the Acute Demands of Short-HIIT

The moderating effects of the athlete competitive level on the acute physiological and perceptual demands of short-HIIT are presented in Table S8 of the ESM. All effects were evaluated as the change in each outcome measure when compared to the recreational competitive level. In trained athletes, there was a substantial increase in HR_peak_ (estimate: 8; 90% CI 0–16 b·min^−1^), peak $\dot{V}$O_2_ (10.2; 0.5–19.9 mL·kg^−1^·min^−1^) and the *T* > 95% $\dot{V}$O_2max_/exercise time ratio (0.17; 0.00–0.35). Additionally, in trained athletes, there was compatibility with a trivial to substantial decrease in sRPE (− 0.6; − 1.9 to 0.6 au). In national athletes, there was a substantial increase in HR_peak_ (10; 90% CI 1–19 b·min^−1^), and peak $\dot{V}$O_2_ (15.6; 3.1–28.2 mL·kg^−1^·min^−1^), as well as compatibility with a trivial to substantial decrease in sRPE (− 1.2; − 2.5 to 0.2 au).

### Moderating Effects of Fitness Level on the Acute Demands of Short-HIIT

The moderating effects of fitness level (i.e. $\dot{V}$O_2max_) on the acute physiological and perceptual demands of short-HIIT are presented in Table S9 of the ESM. All effects were evaluated as the change in each outcome measure when compared to an athlete $\dot{V}$O_2max_ category of 50–55 mL·kg^−1^·min^−1^. In the athlete $\dot{V}$O_2max_ category of 55–60 mL·kg^−1^·min^−1^, there was a substantial decrease in sRPE (estimate: − 1.7; 90% CI − 2.9 to − 0.4 au), but a substantial increase in *T* > 90% $\dot{V}$O_2max_ (125; 9–240 s), as well as compatibility with trivial to substantial increases in *T* > 95% $\dot{V}$O_2max_ (83; − 8 to 174 s) and peak $\dot{V}$O_2_ (5.0; − 0.3 to 10.2 mL·kg^−1^·min^−1^). In the athlete $\dot{V}$O_2max_ category of > 60 mL·kg^−1^·min^−1^, there was a substantial decrease in sRPE (− 1.6; − 2.7 to − 0.6 au) and a substantial increase in peak $\dot{V}$O_2_ (8.5; 2.2–14.8 mL·kg^−1^·min^−1^). All other effects were inconclusive.

### Sensitivity Analysis: Removal of Protocols Performed to Exhaustion

The sensitivity analysis restricted to protocols with a fixed number of repetitions per set is presented in Table S10 of the ESM. Compared to the primary meta-analysis (Table 2), restricting analyses to fixed-repetition protocols resulted in substantial decreases in average $\dot{V}$O₂ (estimate: − 4.7% $\dot{V}$O_2max_), peak $\dot{V}$O₂ (− 2.0 mL·kg⁻^1^·min⁻^1^) and *T* > 90% $\dot{V}$O_2max_ (− 67 s), but a substantial increase in *T* > 95% $\dot{V}$O_2max_ (58 s). Additionally, effects were compatible with trivial to substantial decreases in B[La] (− 0.8 mmol·L⁻^1^) and the *T* > 90% $\dot{V}$O_2max_/exercise time ratio (− 0.04), as well as compatibility with a trivial to substantial increase in the *T* > 95% $\dot{V}$O_2max_/exercise time ratio (0.05). In contrast, HR_avg_, HR_peak_, average $\dot{V}$O_2_ (mL·kg⁻^1^·min⁻^1^), peak $\dot{V}$O_2,_ (%$\dot{V}$O_2max_) and sRPE demonstrated trivial changes, indicating the robustness of these outcomes.

## Discussion

Based on data from 681 athletes and 117 training protocols within 46 studies, this investigation provides a comprehensive synthesis of the acute physiological and perceptual demands of short-HIIT. Pooled effect estimates demonstrate that short-HIIT elicits a substantial aerobic and anaerobic stimulus, though some outcomes vary considerably between studies (Table 2). Using multi-level mixed meta-regression, we identified key factors influencing this variation, including programming variables, athlete fitness level and athlete competitive level. Trained, national-level, and fitter athletes ($\dot{V}$O_2max_ of > 55 mL·kg^−1^·min^−1^) perceived sessions as less demanding, while athletes with a $\dot{V}$O_2max_ of 55–60 mL·kg^−1^·min^−1^ experienced meaningful increases in *T* > 90% $\dot{V}$O_2max_ and *T* > 95% $\dot{V}$O_2max_ compared with athletes with a *V*O_2max_ of 50–55 mL·kg^−1^·min^−1^. Compared to the reference protocol, an active rest period, extending repetition duration by 15 s (i.e. 30 s effort with 30 s rest) or increasing repetition intensity by 5% of MAS enhanced *T* > 90% $\dot{V}$O_2max_ and improved training efficiency (*T* > 90% $\dot{V}$O_2max_/exercise time ratio) without causing a meaningful increase in sRPE. Shuttle runs were associated with the greatest increase in B[La], though they also resulted in a 1.7 ± 0.4 au increase in sRPE. Prescribing 6–12 repetitions per set provides a substantial physiological stimulus, but the effects of performing two more repetitions per set (i.e. 14 repetitions) on all outcomes were compatible with trivial effects. These findings offer practitioners valuable insights into the expected physiological and perceptual demands of short-HIIT across different athlete characteristics, enabling more precise training prescription through targeted manipulation of programming variables.

Time spent near $\dot{V}$O_2max_ is important for maximising cardiovascular and peripheral adaptations during HIIT [3, 25, 26, 79, 80]. Based on 30 samples from 16 studies, our meta-analysis shows that athletes accumulated 259 ± 57 s above 90% $\dot{V}$O_2max_. This meets recommended guidelines that athletes should sustain at least several minutes per session above this threshold during HIIT [3, 25, 26, 81]. As the majority of studies (26 out of 30 samples) implemented a single set of intervals, prescribing multiple sets could allow athletes to accumulate around 10 min above 90% $\dot{V}$O_2max_, aligning with recommendations [4]. This duration exceeds that of repeated-sprint training and sprint interval training, which typically incur less than 60 s above 90% of $\dot{V}$O_2max_ [16, 18, 19, 82], potentially explaining the greater aerobic fitness improvements that are often attained from short-HIIT compared with these other formats [11, 14]. Compared with long-bout HIIT, cardiopulmonary demands can vary considerably within [41] and across studies [4, 31, 35, 45, 83–85], suggesting a need for futher research to form more conclusive opinions. Heart rate demands of short-HIIT were also substantial, with our meta-analysis demonstrating a HR_avg_ of 88 ± 2% HR_max_ and a HR_peak_ of 94 ± 2% HR_max_. Given that heart rate monitoring is widely used in real-world training environments by both practitioners and athletes [86], these findings provide guidance for ensuring that short-HIIT sessions meet their intended physiological targets.

In team sports, conditioning coaches often have only 10–20 min per session to improve players’ fitness, making it crucial to maximise training efficiency. Our review is the first to meta-analyse the proportion of time athletes spend near $\dot{V}$O_2max_ relative to total HIIT session duration, referred to as the time at $\dot{V}$O_2max_/exercise time ratio [3]. When expressed as a percentage, we found that 34 ± 9% of short-HIIT is performed above 90% $\dot{V}$O_2max_, while 15 ± 7% is completed above 95% $\dot{V}$O_2max_. However, this analysis includes protocols where repetitions were performed to exhaustion, which are uncommon in real-world training enviroments. When restricting analysis to fixed-repetition protocols (Sec. 3.10), 30 ± 10% and 24 ± 12% of session time was spent above 90% and 95% of $\dot{V}$O_2max_, respectively. To optimise this ratio (i.e. enhance training efficiency), prescribing longer work intervals (e.g. 30 s of effort), incorporating active inter-repetition rest or an increase in repetition intensity are the most effective strategies. When active recovery is combined with 30-s repetitions, it is possible that more than 50% of a short-HIIT session can be spent above 90% $\dot{V}$O_2max_ [15, 37, 41].

Controlling the level of anaerobic glycolytic energy contribution during HIIT sessions is another important consideration for training design [10]. The pooled-effect estimate of 7.3–9.3 mmol·L^−1^ for B[La] is well above the second lactate threshold (~ 4 mmol·L^−1^) and can be considered a ‘moderate’ level of anaerobic glycolytic energy contribution [4], noting that measurements were taken at the end of sets. This demand is lower than that of sprint-interval training and repeated-sprint training, where B[La] often exceeds 10 mmol·L^−1^ [4, 18, 83]. Short-HIIT therefore serves as a “middle ground” in lactate production among HIIT methods, delivering a considerable anaerobic stimulus that could drive adaptations in glycolytic enzyme activity [87, 88]. Lower-lactate sessions may be beneficial during competition periods when recovery is a priority, whereas higher-lactate sessions could be utilised during preparation phases to maximise adaptation and condition athletes for the high glycolytic demands of competition [89]. Practitioners should be aware that sessions inducing higher lactate levels are typically associated with greater perceived exertion [90, 91]. The pooled-effect estimate for sRPE was 7.1 ± 0.5 au, classifying it as ‘very hard’ (90% prediction interval: range from ‘hard’ to ‘maximal’), which is similar to other HIIT methods [11, 16, 18, 32, 36, 82, 85, 92]. For example, a recent meta-analysis on repeated-sprint training reported a comparable sRPE estimate of 6.5 ± 0.5 au (CR10^®^ units) [18].

A practical approach to addressing heterogeneity in meta-analyses is through prediction intervals, which estimate the likely effect size of a future similar study based on the included studies. This provides practitioners with insight into the expected variability in training outcomes [93]. Prediction intervals for the meta-analysed effects of short-HIIT on acute physiological and perceptual demands are reported in Table 2. As expected, these intervals were wider than our CIs, indicating greater uncertainty in some outcomes. Notably, the prediction intervals for *T* > 90% $\dot{V}$O_2max_ and *T* > 95% $\dot{V}$O_2max_ revealed substantial variability, both between and within studies. For example, in Dupont et al. [34], *T* > 90% $\dot{V}$O_2max_ ranged from 77 to 383 s across four different short-HIIT protocols, with some athletes not reaching $\dot{V}$O_2max_. This highlights the importance of considering inter-individual differences in physiology (e.g. fitness levels, muscle fibre typology) and the impact of programming variables when designing short-HIIT programmes.

### Moderating Effects of Programming Variables

**Fig. 19:**
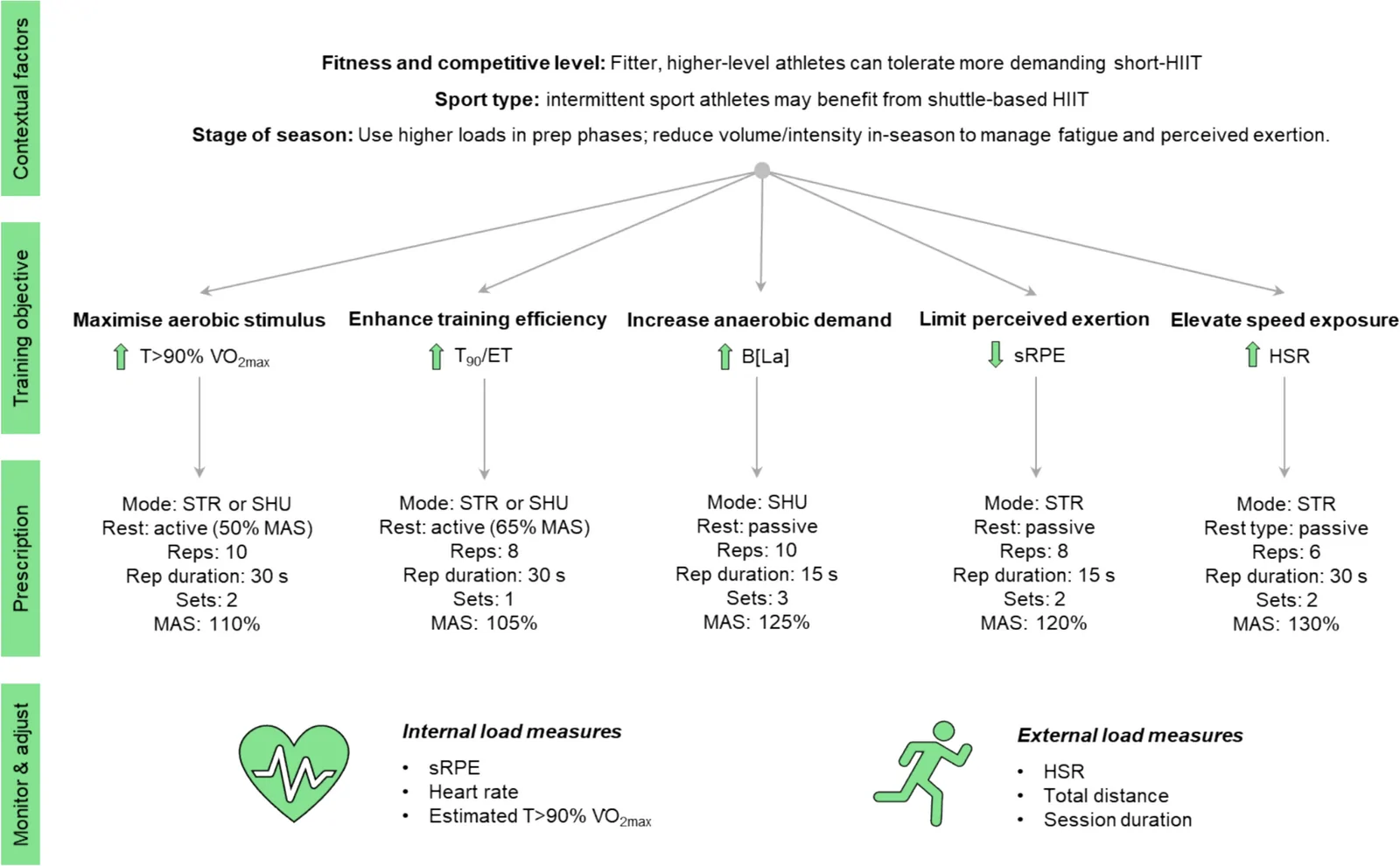
Decision-making framework for prescribing short-bout high-intensity interval training (HIIT). Note that the sessions provided are illustrative examples and should be adapted to reflect the full range of contextual factors and training objectives relevant to each specific scenario. *B[La]* blood lactate concentration, *HSR* high-speed running *prep* preparation, *MAS* maximal aerobic speed, *reps* repetitions, *SHU* shuttle running, *sRPE* session ratings of perceived exertion, *STR* straight-line running, *T* > *90% VO*_*2max*_ time above 90% of maximal oxygen consumption, *T*_90_*/ET* time above 90% of maximal oxygen consumption/exercise time ratio

The moderating effects of programming variables on the acute demands of short-HIIT are summarised in Table 3 and Sects. 4.1.1 to Sects. 4.1.6. Furthermore, a decision-making framework for prescribing short-HIIT sessions based on the consideration of contextual factors, training objectives and manipulation of programming variables is presented in Fig. 19.

**Table 3:** Summary of the effects of programming variables on the acute demands of short-bout high-intensity interval training

	B[La]	HR_avg_	HR_peak_	$\dot{V}$O_2avg_	$\dot{V}$O_2peak_	*T* > 90% $\dot{V}$O_2max_	*T* > 95% $\dot{V}$O_2max_	*T*_90_/ET	*T*_95_/ET	sRPE
Shuttle runs	**↑↑**	*	*	*	*	*	*	*	*	**↑↑**
Active rest	?	*	*	*	*	**?**	*	**↑↑**	*	=
2 more repetitions	=	=	=	=↓	?	=	=	=	=	=
+ 15 s interval	?	=↓	?	?	?	**↑↑**	?	**?**	?	=
1 more set	**↑↑**	*	=**↑**	*	*	*	*	*	*	*
+ 5% MAS	**↑↑**	**↑↑**	=**↑**	=**↑**	?	?	=↓	**↑↑**	?	=

Acute demands based on meta-analytic inferences and compared to the reference protocol of 1 set of 12 straight-line repetitions, performed at 120% MAS for a 15-s work duration with 15 s of passive rest

*B[La]* blood lactate, *HR*_*avg*_ average heart rate, *HR*_*peak*_ peak heart rate, *MAS* maximal aerobic speed, $\dot{V}$*O*_*2avg*_ average oxygen consumption, $\dot{V}$*O*_*2peak*_ peak oxygen consumption, *T* > *90%* $\dot{V}$O_2max_ time above 90% of maximal oxygen consumption, *T* > *95% *$\dot{V}$*O*_*2max*_ time above 95% of maximal oxygen consumption, *T*_90_*/ET* time above 90% of maximal oxygen consumption/exercise time ratio; *T*_95_*/ET* time above 95% of maximal oxygen consumption/exercise time ratio, *sRPE* session ratings of perceived exertion, = indicates a trivial effect; ↑↑ substantial increase; ↓↓ indicates a substantial decrease; = ↓ indicates compatibility with a trivial to substantial decrease; = ↑ indicates compatibility with a trivial to substantial increase; ? indicates inconclusive; * indicates that the effects were not evaluated because of an insufficient amount of samples

#### Running Modality

Studies more commonly prescribed straight-line runs (*n* = 88/117) than shuttle runs (*n* = 29/117). Among the 29 shuttle-run protocols, the number of changes of direction per repetition ranged from 1 to 3. Compared with straight-line runs, our meta-analysis demonstrated that shuttle-based HIIT resulted in a 1.9 ± 1.0 mmol·L⁻^1^ increase in B[La], which was a considerably greater effect than modifying other programming variables. However, shuttle runs also led to a substantial increase in sRPE. When compared with the reference protocol of 1 set of 12 straight-line repetitions, performed at 120% MAS, with a 15-s work duration and 15 s of passive rest, shuttle-based HIIT would likely elicit a B[La] of ~ 10 mmol·L⁻^1^ and an sRPE of 7–10 au (CR10^®^ units). This anaerobic demand would be classified as high [4], with perceived exertion very hard to maximal. Because of an insufficient number of samples, acute cardiopulmonary outcomes were not analysed, highlighting the need for further research on the physiological demands of shuttle-based HIIT.

Shuttle-based HIIT is particularly relevant for intermittent sports (e.g. soccer, basketball, tennis), where frequent changes of direction occur under accumulating fatigue [94]. Practitioners should incorporate shuttle runs when a greater anaerobic glycolytic stimulus is desired, along with the increased neuromuscular load from repeated accelerations and decelerations [95]. However, they should also consider that shuttle-based HIIT may impose near-maximal perceptual demands. Given these factors, shuttle runs are better suited for preparation phases rather than in-season training. Conversely, straight-line runs, which induce a lower internal load, are recommended when the goal is to maintain fitness while minimising fatigue (e.g. glycogen depletion, perceived stress) during the competitive season [96, 97].

#### Rest Modality

A greater number of short-HIIT protocols implemented passive inter-repetition rest (*n* = 78/117) compared to active rest (*n* = 39/117). Among all programming variables analysed, prescribing an active recovery period was the most effective strategy for improving training efficiency (i.e. increasing the *T* > 90% $\dot{V}$O_2max_/exercise time ratio). Our meta-analytic estimate suggests that incorporating active rest allows athletes to spend a further 21 ± 11% of the reference session above 90% of $\dot{V}$O_2max_ and to accumulate an additional 85 ± 114 s per set within this extreme intensity domain. This is likely due to active recovery accelerating the time taken to reach 90% $\dot{V}$O_2max_ and limiting the drop in cardiorespiratory demand between repetitions [10, 37]. One might expect active rest periods to increase perceived exertion. However, despite a significant proportion of the sample consisting of recreational-level athletes, who typically perceive HIIT as more challenging, the findings suggest that active rest periods had trivial effects on sRPE. There has been conjecture on the effects of rest modality on B[La] [4], but as active rest was associated with a − 0.5 ± 1.2 mmol·L^−1^ change, the uncertainty in this outcome suggests that its effects remain inconclusive.

Our analysis does not account for variations in recovery intensity, which ranged from 50 to 84% MAS across studies [9, 15, 21, 30, 31, 35, 37, 39–41, 43, 98–103]. Only one study has directly examined the effects of different active recovery intensities during short-HIIT [37], demonstrating substantial impacts on exercise capacity and time near $\dot{V}$O_2max_. During a 30/30 s set to exhaustion, lower recovery intensities (84% vs 67% vs 50% MAS) increased the number of completed repetitions per set from 7 to 12 to 18, and consequently enhanced the *T* > 90% $\dot{V}$O₂_max_ [37]. However, the *T* > 90% $\dot{V}$O_2max_/exercise time ratio increased substantially with higher recovery intensity [37]. For coaches aiming to maximise the aerobic stimulus within a limited conditioning window, prescribing higher active recovery intensities may be a viable strategy, although this approach will require a reduction in both repetitions and intensity. In this context, an effective prescription could involve 8 × 30 s repetitions at 105% MAS, interspersed with 30 s of active recovery at 65% MAS.

#### Number of Repetitions

Because of the inclusion of studies that examined short-HIIT to exhaustion, there was considerable variability in the number of repetitions completed per set, which ranged from 3 to 82 repetitions. However, the prescription of 20 repetitions (*n* = 13/117 protocols) and 12 repetitions (*n* = 11/117 protocols) was most common. Our meta-analysis indicated that performing two more repetitions per set (i.e. 14 repetitions) was compatible with a trivial to substantial decrease in average $\dot{V}$O_2_, showed inconclusive effects on peak $\dot{V}$O_2_ and had trivial effects on all other outcome measures (Table 3). These results align with findings from a meta-analysis on repeated-sprint training, which reported no meaningful changes in B[La], HR_peak_ and sRPE when two additional repetitions were performed per set [17]. As prescribing 6–12 repetitions per set already provides a substantial physiological stimulus, adjusting other programming variables will have a greater impact on the acute demands of short-HIIT.

The number of repetitions should align with the session goals (e.g. high-speed running load, *T* > 90% $\dot{V}$O_2max_, *T* > 90% $\dot{V}$O_2max_/exercise time ratio) and the athlete physiological profile (i.e. speed, endurance, hybrid [3, 104]). A prescription of 6–8 repetitions per set is suitable during competition periods or at the beginning of a short-HIIT programme before gradually increasing the training volume. To maximise the physiological stimulus during low-repetition sets, higher intensities, longer efforts (e.g. 30–45 s) and/or active recoveries should be incorporated. Athletes with higher MAS but a lower maximal sprinting speed (i.e. endurance profile) may benefit more from higher-repetition sets (e.g. 12 repetitions) at a lower work interval intensity [104]. Given their greater reliance on aerobic metabolism [32], endurance athletes can tolerate higher training volumes but may experience greater neuromuscular fatigue and muscle damage when short-HIIT is performed at high intensities [105]. Larger repetition sets may also be more effective during preparation periods, where increased fatigue can drive greater physiological adaptation.

#### Repetition Duration

Most protocols prescribed either 15-s repetitions with 15 s of rest or 30-s repetitions with 30 s of rest (*n* = 62 and 41 protocols, respectively). Our meta-analysis demonstrated that when compared with the reference session, prescribing 30-s repetitions with 30 s of rest resulted in a substantially greater *T* > 90% of $\dot{V}$O_2max_. Furthermore, the *T* > 90% $\dot{V}$O_2max_/exercise time ratio was increased by 7 ± 10%. Despite these differences in the physiological stimulus, extending repetition duration did not meaningfully increase sRPE (Figs. 17 and 18). This may be because longer repetitions were accompanied by proportionally longer inter-repetition recovery periods, as the 1:1 work-to-rest ratio was maintained. From an applied perspective, these results indicate that longer repetition durations can be used to maximise *T* > 90% $\dot{V}$O₂_max_, potentially improve training efficiency and increase high-speed running exposure during short-HIIT without a concomitant increase in sRPE. However, modest reductions in repetition number or intensity may be required to ensure task completion and preserve session quality.

#### Number of Sets

A single set of intervals was the most prescribed protocol (*n* = 80/117). Because of a limited number of studies examining multiple-set protocols, there were only sufficient data to evaluate their effects on B[La] and HR_peak_. While our reference estimate suggested that adding an additional set during short-HIIT could increase HR_peak_ by 3.9 b·min⁻^1^, the wide CI (− 1.2 to 9.0 b·min⁻^1^) indicates that this programming manipulation is also compatible with trivial effects. However, practitioners can expect a 1.2 ± 0.1 mmol·L⁻^1^ increase in B[La] per additional set, potentially leading to B[La] levels ≥ 10 mmol·L⁻^1^ when two sets of 12 straight-line repetitions at 120% MAS with 15 s of work and 15 s of passive rest are prescribed. As fatigue accumulates during high-intensity exercise, reliance on anaerobic glycolysis increases, leading to elevated lactate production and glycogen depletion [89]. Additionally, greater recruitment of motor units increases aerobic metabolism when sets are repeated [106]. Multiple sets of short-HIIT may be particularly useful during preparation periods when higher training volumes and greater physiological stress could drive enhanced adaptation [107–109]. Conversely, prescribing a single set during competition periods can provide an acute physiological stimulus while limiting fatigue and maintaining exposure to high-speed running.

#### Repetition Intensity

The training protocols in our studies used a variety of field- and laboratory-based fitness tests to prescribe intensity. After adjusting all intensities to represent the lowest velocity that elicits $\dot{V}$O_2max_, values ranged from 85 to 140% MAS, with 120% MAS (*n* = 23 protocols) and 110% MAS (*n* = 18 protocols) the most common. Repetition intensity had a strong influence on physiological outcomes. A 5% increase in MAS led to substantial increases in B[La], HR_avg_ and the *T* > 90% $\dot{V}$O_2max_/exercise time ratio, while also showing compatibility with trivial to substantial increases in HR_peak_ and average $\dot{V}$O_2_. Although a 5% increase in MAS had little effect on sRPE, a 10% increase could increase sRPE by one unit (i.e. from 7.6 to 8.6 au). These findings suggest that small changes in running speed can substantially increase the metabolic and cardiorespiratory response to HIIT, highlighting the need for accurate testing and understanding of an individual’s MAS is needed to ensure the desired physiological stimulus. Furthermore, because the ASR accounts for an athlete’s maximal sprinting speed in addition to MAS, it can provide a more precise basis for prescribing short-HIIT, reducing inter-individual variation in psychophysiological responses and physical adaptations [46, 70, 105, 110, 111].

### Moderating Effects of Fitness Level and Competitive Level

Our investigation revealed that athletes at the recreational level and with lower fitness experienced elevated sRPE. For instance, athletes with a $\dot{V}$O_2max_ of 50–55 mL·kg^−1^·min^−1^ rated short-HIIT as very hard (7.9 ± 1.0 au), while athletes with a $\dot{V}$O_2max_ of 55–60 mL·kg^−1^·min^−1^ and 60–65 mL·kg^−1^·min^−1^ perceived it as less demanding (6.2 ± 1.3 au and 6.3 ± 1.1 au, respectively). Although these findings do not account for programming variables and other contextual factors, a visual inspection of the data (Table S7 of the ESM) suggests that the training protocols undertaken by recreational athletes and athletes in the 50–55 mL·kg⁻^1^·min⁻^1^ category were no more demanding than those completed by athletes in the higher competitive level and fitness categories. Ratings of perceived exertion reflect the interplay of the psychophysiological stress experienced during exercise [112]. Whilst not consistently observed [113], previous studies have shown that at the same relative exercise intensity, those with a higher level of fitness report a lower sRPE [114]. When designing short-HIIT programmes for athletes with lower fitness levels, practitioners may consider prescribing straight-line runs and/or lower running intensities initially, as these adjustments can help reduce perceived exertion. Conversely, more physiologically and perceptually demanding sessions can be implemented for fitter athletes, who have a greater tolerance for short-HIIT.

Compared with the recreational competitive level, trained athletes experienced substantially greater HR_peak_, peak $\dot{V}$O_2_ and *T* > 95%$\dot{V}$O_2max_/exercise time ratio during short-HIIT. Although it is intuitive to attribute these differences to a higher $\dot{V}$O_2max_ in trained athletes, they may also reflect faster $\dot{V}$O_2_ kinetics observed in trained athletes at the onset of exercise [115]. As trained athletes can rapidly respond to the oxygen demands of the short-HIIT, a greater amount of time will be spent at a higher $\dot{V}$O_2_. Additionally, with increased levels of training, athletes have a reduced slow (or primary) component of $\dot{V}$O_2_, which has been associated with a greater ability to accumulate time at $\dot{V}$O_2max_ during short-HIIT [101, 116]. Moreover, with an enhanced oxidative capacity, trained athletes are likely to have an accelerated recovery between intervals, enabling the continued utilisation of aerobic energy pathways to maintain the target running velocity [117–119]. Combined, the ability to rapidly respond to the increased oxygen demands of HIIT supports the greater $\dot{V}$O_2_ response observed in trained athletes. These findings suggest that when prescribing short-HIIT, it is essential to consider both the athlete’s fitness level and competitive level to optimise training outcomes.

### Limitations

Several limitations should be considered when interpreting our findings. First, some variation in the acute demands of short-HIIT may be influenced by factors beyond those analysed, including age, training status, sex and ethnicity. Additionally, differences in data collection methods, environmental conditions and reporting practices may have contributed to variability, although we endeavoured to minimise these influences through our inclusion and exclusion criteria. Second, while we examined the combined effects of multiple programming variables, there were insufficient data to analyse the effects of shuttle runs, active rest and additional sets on some outcomes, highlighting the need for further research in these areas. Third, the conversion of field-based fitness test velocities to MAS, while informed by validated studies, introduces a layer of estimation that may affect the precision of the intensity moderator. Fourth, because of the lack of benchmarks for practically significant changes in several of our acute outcomes (e.g. $\dot{V}$O_2_), we relied on standardised effect sizes to assess the magnitude of change. Practitioners may need to determine their own thresholds for what constitutes a meaningful change in these metrics. Finally, 40 protocols included in our meta-analysis assessed short-HIIT using runs performed to exhaustion, which may overestimate certain physiological demands. To address this potential bias, we conducted a sensitivity analysis comparing the primary meta-analysis with an analysis restricted to protocols prescribing a fixed number of repetitions (Sect. 3.10; Table S10 of the ESM).

## Conclusions

Our systematic review and meta-analysis summarises the acute physiological and perceptual demands of short-HIIT in athletes, providing a quantitative synthesis of the moderating effects of programming variables, athlete fitness level and performance calibre. Short-HIIT elicits a substantial physiological stimulus, with trained athletes experiencing greater demands than recreational athletes, while fitter individuals perceive the sessions as less strenuous. To maximise cardiopulmonary demand and training efficiency, increasing the number of repetitions per set has minimal impact. Instead, the most effective programming strategies include implementing active inter-repetition rest, extending repetition duration and increasing repetition intensity. Ultimately, HIIT program design should be tailored to individual and contextual factors such as training status, athlete physiology and specific goals. Further research is needed to clarify the impact of fitness level on the acute demands of short-HIIT, as many outcomes remain inconclusive. Our findings provide practitioners with expected physiological and perceptual demands across different athlete characteristics, enabling more precise and effective training prescriptions through strategic manipulation of programming variables.

## Supplementary Information

Below is the link to the electronic supplementary material.

Supplementary file1 (DOCX 689 KB)

## Declarations

### Availability of Data and Material

All data and material reported in this review are from peer-reviewed publications. All extracted data are available in Tables S6 and S7 of the ESM.
