# Supplementary material for: The Acute Effects of Short-Bout High-Intensity Interval Training on Physiological and Perceptual Outcomes: A Systematic Review and Meta-analysis
Source: Sports Med. 2026 May 22;56(10):2497–526. doi: 10.1007/s40279-026-02456-x (PMC13615151; doi:10.1007/s40279-026-02456-x)
Supplement: Supplementary file 1 [file 40279_2026_2456_MOESM1_ESM.docx]

**Full title:**	The Acute Effects of Short-Bout High-Intensity Interval Training on Physiological and Perceptual Outcomes: A Systematic Review and Meta-Analysis

**Running heading:**	Acute Effects of Short-HIIT

**Authors:**	Fraser Thurlow^1^, Nicholas Cowley^2,3^, Katie Slattery^1^, Jonathon Weakley^2,3,4^

**Affiliations:**

^1^ School of Sport, Exercise and Rehabilitation, University of Technology Sydney, Sydney, New South Wales, Australia

^2^ School of Behavioural and Health Sciences, Australian Catholic University, Brisbane, Queensland, Australia

^3^ Sports Performance, Recovery, Injury and New Technologies (SPRINT) Research Centre, Australian Catholic University, Brisbane, QLD, Australia

^4^ Carnegie Applied Rugby Research (CARR) Centre, Carnegie School of Sport, Leeds, United Kingdom

**Corresponding Author:**

Fraser Thurlow

School of sport, Exercise and Rehabilitation

University of Technology Sydney

UTS-Rugby Australia Building, Moore Park Rd, 2021

New South Wales

AUSTRALIA

E: fraser.thurlow@uts.edu.au

**Supplementary Table S1.** Search strategy for Pubmed.

| Search number | Search phrase | Items found |
| --- | --- | --- |
| 1 | ("High-Intensity Interval Training/classification"[Mesh] OR "High-Intensity Interval Training/instrumentation"[Mesh] OR "High-Intensity Interval Training/methods"[Mesh] OR "supramaximal interval" OR "supramaximal training" OR "supramaximal running" OR "supramaximal exercise" OR "intermittent training" OR "intermittent running" OR "intermittent exercise") | 3227 |
| 2 | ("High-Intensity Interval Training/classification"[Mesh] OR "High-Intensity Interval Training/instrumentation"[Mesh] OR "High-Intensity Interval Training/methods"[Mesh] OR "supramaximal interval" OR "supramaximal training" OR "supramaximal running" OR "supramaximal exercise" OR "intermittent training" OR "intermittent running" OR "intermittent exercise") AND ("Physiological" OR "perceptual" OR "metabolic" OR "cardiorespiratory" OR "blood lactate" OR "heart rate" OR "perceived exertion" OR "V̇O2" OR "oxygen consumption" OR "oxygen uptake") | 2158 |
| 3 | ("High-Intensity Interval Training/classification"[Mesh] OR "High-Intensity Interval Training/instrumentation"[Mesh] OR "High-Intensity Interval Training/methods"[Mesh] OR "supramaximal interval" OR "supramaximal training" OR "supramaximal running" OR "supramaximal exercise" OR "intermittent training" OR "intermittent running" OR "intermittent exercise") AND ("Physiological" OR "perceptual" OR "metabolic" OR "cardiorespiratory" OR "blood lactate" OR "heart rate" OR "perceived exertion" OR "V̇O2" OR "oxygen consumption" OR "oxygen uptake") NOT ("cycling" OR "swimming" OR "ergometer" OR "ski" OR "untrained" OR "sedentary" OR "children" OR "patient*" OR "obese*") | 1189 |

**Supplementary Table S2.** Search strategy for CINAHL

| Search  number | Search phrase | Items found |
| --- | --- | --- |
| 1 | (MH "High-intensity interval training/UT/PH" OR "supramaximal interval" OR "supramaximal training" OR "supramaximal running" OR "supramaximal exercise" OR "intermittent training" OR "intermittent running" OR "intermittent exercise") | 829 |
| 2 | (MH "High-intensity interval training/UT/PH" OR "supramaximal interval" OR "supramaximal training" OR "supramaximal running" OR "supramaximal exercise" OR "intermittent training" OR "intermittent running" OR "intermittent exercise") AND (“Physiological” OR “perceptual” OR “metabolic” OR “cardiorespiratory” OR “blood lactate” OR “heart rate” OR “perceived exertion” OR “V̇O_2_” OR “oxygen consumption” OR “oxygen uptake”) | 523 |
| 3 | (MH "High-intensity interval training/UT/PH" OR "supramaximal interval" OR "supramaximal training" OR "supramaximal running" OR "supramaximal exercise" OR "intermittent training" OR "intermittent running" OR "intermittent exercise")AND (“Physiological” OR “perceptual” OR “metabolic” OR “cardiorespiratory” OR “blood lactate” OR “heart rate” OR “perceived exertion” OR “V̇O_2_” OR “oxygen consumption” OR “oxygen uptake”) NOT (“cycling” OR “swimming” OR “ergometer” OR “ski” OR “untrained” OR “sedentary” OR “children” OR “patient*” OR “obese*”) | 303 |
| 4 | Filter: academic journals | 293 |

**Supplementary Table S3.** Search strategy for SPORTDiscus.

| Search number | Search phrase | Items found |
| --- | --- | --- |
| 1 | (DE "HIGH-intensity interval training" OR DE "INTERVAL training" OR "supramaximal interval" OR "supramaximal training" OR "supramaximal running" OR "supramaximal exercise" OR "intermittent training" OR "intermittent running" OR "intermittent exercise") | 5341 |
| 2 | (DE "HIGH-intensity interval training" OR DE "INTERVAL training" OR "supramaximal interval" OR "supramaximal training" OR "supramaximal running" OR "supramaximal exercise" OR "intermittent training" OR "intermittent running" OR "intermittent exercise") AND (“Physiological” OR “perceptual” OR “metabolic” OR “cardiorespiratory” OR “blood lactate” OR “heart rate” OR “perceived exertion” OR “V̇O_2_” OR “oxygen consumption” OR “oxygen uptake”) | 3009 |
| 3 | (DE "HIGH-intensity interval training" OR DE "INTERVAL training" OR "supramaximal interval" OR "supramaximal training" OR "supramaximal running" OR "supramaximal exercise" OR "intermittent training" OR "intermittent running" OR "intermittent exercise") AND (“Physiological” OR “perceptual” OR “metabolic” OR “cardiorespiratory” OR “blood lactate” OR “heart rate” OR “perceived exertion” OR “V̇O_2_” OR “oxygen consumption” OR “oxygen uptake”) NOT (“cycling” OR “swimming” OR “ergometer” OR “ski” OR “untrained” OR “sedentary” OR “children” OR “patient*” OR “obese*”) | 1759 |
| 4 | Filter: academic journals | 1659 |

**Supplementary Table S4.** Modified Downs and Black scale outcomes for the assessment of reporting quality and risk of bias.

| **Study** | **Item number** | | | | | | | | | | | | | | **Total score**  **(out of 14)** |
| --- | --- | --- | --- | --- | --- | --- | --- | --- | --- | --- | --- | --- | --- | --- | --- |
|  | **1** | **2** | **3** | **6** | **7** | **10** | **12** | **15** | **16** | **18** | **20** | **22** | **23** | **25** |  |
| Abderrahman et al. [15] | 1 | 1 | 1 | 1 | 1 | 1 | 0 | 0 | 1 | 1 | 1 | 1 | 1 | 0 | 11 |
| Al Haddad et al. [98] | 1 | 1 | 1 | 1 | 1 | 1 | 0 | 0 | 1 | 1 | 1 | 1 | 0 | 1 | 11 |
| Alves et al. [120] | 1 | 1 | 0 | 1 | 1 | 1 | 0 | 0 | 1 | 1 | 1 | 1 | 0 | 1 | 10 |
| Arantes et al. [8] | 1 | 1 | 1 | 1 | 1 | 1 | 0 | 0 | 1 | 1 | 1 | 1 | 0 | 1 | 11 |
| Billat et al. [30] | 1 | 1 | 1 | 1 | 1 | 1 | 0 | 0 | 1 | 1 | 1 | 1 | 0 | 1 | 11 |
| Bok et al. [20] | 1 | 1 | 1 | 1 | 1 | 1 | 0 | 0 | 1 | 1 | 1 | 1 | 0 | 1 | 11 |
| Bok et al. [46] | 1 | 1 | 1 | 1 | 1 | 1 | 0 | 0 | 1 | 1 | 1 | 1 | 0 | 1 | 11 |
| Buchheit et al. [121] | 1 | 1 | 1 | 1 | 1 | 1 | 0 | 0 | 1 | 1 | 1 | 1 | 0 | 1 | 11 |
| Buchheit et al. [45] | 1 | 1 | 1 | 1 | 1 | 1 | 0 | 0 | 1 | 1 | 1 | 1 | 0 | 1 | 11 |
| Chambion-Diaz et al. [122] | 1 | 1 | 0 | 1 | 1 | 1 | 0 | 0 | 1 | 1 | 1 | 1 | 0 | 1 | 10 |
| Cipryan et al. [31] | 1 | 1 | 1 | 1 | 1 | 1 | 0 | 0 | 1 | 1 | 1 | 1 | 0 | 1 | 11 |
| Cipryan et al. [32] | 1 | 1 | 1 | 1 | 1 | 1 | 0 | 0 | 1 | 1 | 1 | 1 | 0 | 1 | 11 |
| Da Silva et al. [123] | 1 | 1 | 1 | 1 | 1 | 0 | 0 | 0 | 1 | 1 | 1 | 0 | 1 | 1 | 10 |
| Delextrat et al. [103] | 1 | 1 | 1 | 1 | 1 | 1 | 0 | 0 | 1 | 1 | 1 | 0 | 1 | 1 | 11 |
| Dellal et al. [21] | 1 | 1 | 1 | 0 | 0 | 0 | 0 | 0 | 1 | 1 | 1 | 1 | 0 | 0 | 7 |
| Dellal et al. [22] | 1 | 1 | 1 | 1 | 1 | 0 | 0 | 0 | 1 | 1 | 1 | 1 | 0 | 1 | 10 |
| Dellal et al. [124] | 1 | 1 | 1 | 1 | 1 | 1 | 0 | 0 | 1 | 1 | 1 | 1 | 1 | 1 | 12 |
| Ducrocq et al. [33] | 1 | 1 | 1 | 1 | 1 | 1 | 0 | 0 | 1 | 1 | 1 | 1 | 0 | 0 | 10 |
| Dupont & Berthoin [39] | 1 | 1 | 1 | 1 | 1 | 0 | 0 | 0 | 1 | 1 | 1 | 1 | 0 | 1 | 10 |
| Dupont et al. [34] | 1 | 1 | 0 | 1 | 1 | 0 | 0 | 0 | 1 | 1 | 1 | 1 | 0 | 0 | 8 |
| Fleckenstein et al. [102] | 1 | 1 | 0 | 1 | 1 | 1 | 0 | 0 | 1 | 1 | 1 | 1 | 0 | 1 | 10 |
| Julio et al. [70] | 1 | 1 | 1 | 1 | 1 | 1 | 0 | 0 | 1 | 1 | 1 | 1 | 0 | 0 | 10 |
| McEwan et al. [12] | 1 | 1 | 1 | 1 | 1 | 1 | 0 | 0 | 1 | 1 | 1 | 1 | 0 | 1 | 11 |
| McLean et al. [5] | 1 | 1 | 1 | 1 | 1 | 1 | 0 | 1 | 1 | 1 | 1 | 1 | 1 | 1 | 13 |
| Midgley et al. [41] | 1 | 1 | 1 | 1 | 1 | 1 | 0 | 0 | 1 | 1 | 1 | 1 | 0 | 1 | 11 |
| Midgley et al. [40] | 1 | 1 | 1 | 1 | 1 | 1 | 0 | 0 | 1 | 1 | 1 | 1 | 0 | 1 | 11 |
| Millet et al. [35] | 1 | 1 | 1 | 1 | 1 | 0 | 0 | 0 | 1 | 1 | 1 | 1 | 0 | 1 | 10 |
| Millet et al. [101] | 1 | 1 | 1 | 1 | 1 | 0 | 0 | 0 | 1 | 1 | 1 | 1 | 0 | 1 | 10 |
| Naves et al. [36] | 1 | 1 | 1 | 1 | 1 | 0 | 0 | 0 | 1 | 1 | 1 | 1 | 0 | 1 | 10 |
| Ortiz et al. [6] | 1 | 1 | 1 | 1 | 1 | 1 | 0 | 0 | 1 | 1 | 1 | 1 | 1 | 1 | 10 |
| Panascì et al. [13] | 1 | 1 | 1 | 1 | 1 | 1 | 0 | 0 | 1 | 1 | 1 | 1 | 0 | 1 | 11 |
| Psarras et al. [23] | 1 | 1 | 1 | 1 | 1 | 1 | 0 | 0 | 1 | 1 | 1 | 1 | 0 | 1 | 11 |
| Rozenek et al. [42] | 1 | 1 | 1 | 1 | 1 | 0 | 0 | 0 | 1 | 1 | 1 | 1 | 0 | 1 | 10 |
| Seipp et al. [125] | 1 | 1 | 1 | 1 | 1 | 0 | 0 | 0 | 1 | 1 | 1 | 1 | 0 | 1 | 10 |
| Selmi et al. [126] | 1 | 1 | 1 | 0 | 0 | 0 | 0 | 0 | 1 | 1 | 1 | 1 | 0 | 1 | 8 |
| Selmi et al. [127] | 1 | 1 | 1 | 1 | 1 | 1 | 0 | 0 | 1 | 1 | 1 | 1 | 0 | 1 | 11 |
| Selmi et al. [128] | 1 | 1 | 1 | 1 | 1 | 1 | 0 | 0 | 1 | 1 | 1 | 1 | 0 | 1 | 11 |
| Tardieu-Berger et al. [43] | 1 | 1 | 1 | 1 | 1 | 0 | 0 | 0 | 1 | 1 | 1 | 1 | 0 | 0 | 9 |
| Thevenet et al. [37] | 1 | 1 | 1 | 1 | 1 | 0 | 0 | 0 | 1 | 1 | 1 | 1 | 0 | 1 | 10 |
| Thevenet et al. [9] | 1 | 1 | 1 | 1 | 1 | 0 | 0 | 0 | 1 | 1 | 1 | 1 | 0 | 1 | 10 |
| Thurlow et al. [11] | 1 | 1 | 1 | 1 | 1 | 0 | 0 | 0 | 1 | 1 | 1 | 1 | 0 | 1 | 10 |
| Wakefield & Glaister [44] | 1 | 1 | 1 | 1 | 1 | 1 | 0 | 0 | 1 | 1 | 1 | 1 | 0 | 1 | 11 |
| Zafeiridis et al. [100] | 1 | 1 | 1 | 1 | 1 | 0 | 0 | 0 | 1 | 1 | 1 | 1 | 0 | 1 | 10 |
| Zeng et al. [24] | 1 | 1 | 1 | 1 | 1 | 1 | 0 | 0 | 1 | 1 | 1 | 1 | 1 | 0 | 11 |
| Zeng et al. [7] | 1 | 1 | 1 | 1 | 1 | 1 | 0 | 0 | 1 | 1 | 1 | 1 | 1 | 0 | 11 |
| Zouhal et al. [38] | 1 | 1 | 1 | 1 | 1 | 0 | 0 | 0 | 1 | 1 | 1 | 1 | 0 | 1 | 10 |
| Notes: 0 = no; 1 = yes; U = unable to determine. Item 1: clear aim/hypothesis; Item 2: outcome measures clearly described; Item 3: patient characteristics clearly described; Item 6: main findings clearly described; Item 7: measures of random variability provided; Item 10: actual probability values reported; Item 12: participants prepared to participate representative of the entire population; Item 15: blinding of outcome measures; Item 16: analysis completed was planned; Item 18: appropriate statistics; Item 20: valid and reliable outcome measures; Item 22: participants recruited over the same period; Item 23: randomised; Item 25: adjustment made for confounding variables. | | | | | | | | | | | | | | | |

**Supplementary Table S5.** Recommendation, Assessment, Development and Evaluation tool for the assessment of certainty of evidence.

| **Outcome** | **# Studies** | **Criteria** | | | | | | |
| --- | --- | --- | --- | --- | --- | --- | --- | --- |
|  |  | Inconsistency | Risk of bias | Imprecision | Indirectness | Publication bias | Certainty of evidence |  |
| B[La] (mmol∙L^-1^) | 27 | Not serious | Not serious | Not serious | Not serious | Not serious | High |  |
| HR_avg_ (b∙min^-1^) | 14 | Not serious | Not serious | Not serious | Not serious | Not serious | High |  |
| HR_avg_ (%HR_max_) | 14 | Not serious | Not serious | Not serious | Not serious | Not serious | High |  |
| HR_peak_ (b∙min^-1^) | 17 | Not serious | Not serious | Serious | Not serious | Not serious | High |  |
| HR_peak_ (%HR_max_) | 7 | Not serious | Not serious | Not serious | Not serious | Not serious | High |  |
| V̇O_2avg_ (ml∙kg^-1^∙min^-1^) | 8 | Not serious | Not serious | Not serious | Not serious | Not serious | High |  |
| V̇O_2avg_ (%V̇O_2max_) | 7 | Not serious | Not serious | Serious | Not serious | Not serious | High |  |
| V̇O_2peak_ (ml∙kg^-1^∙min^-1^) | 12 | Not serious | Not serious | Not serious | Not serious | Not serious | High |  |
| V̇O_2peak_ (%V̇O_2max_) | 6 | Serious | Not serious | Serious | Not serious | Not serious | Moderate |  |
| T>90%V̇O_2max_ (s) | 15 | Serious | Not serious | Serious | Not serious | Not serious | Moderate |  |
| T>95%V̇O_2max_ (s) | 9 | Serious | Not serious | Serious | Not serious | Not serious | Moderate |  |
| sRPE (au) | 27 | Not serious | Serious | Not serious | Not serious | Not serious | Moderate |  |

Abbreviations: # = number; B[La] = end-set blood lactate concentration; HR_avg_ = average heart rate; %HR_max_ = percentage of maximal heart rate; HR_peak_ = peak heart rate; V̇O_2avg_ = average oxygen consumption; V̇O_2peak_ = peak oxygen consumption; T>90%V̇O_2max_ = time above 90% of maximal oxygen consumption; T>95%V̇O_2max_ = time above 95% of maximal oxygen consumption; sRPE = session ratings of perceived exertion

**Supplementary Table S6.** Summary of athlete and study characteristics.

| **Study** | **Participants** | | | | | | |  | **Experimental Approach** | | |
| --- | --- | --- | --- | --- | --- | --- | --- | --- | --- | --- | --- |
|  | **N** | **Sport** | **Level** | **Age**  **(yrs)** | **Stature**  **(cm)** | **Body mass (kg)** | **V̇O_2max_ (**ml⋅min^-1^⋅kg^-1^) |  | **Design** | **Type** | **Details** |
| Abderrahman et al. [15] | 24 | NR | TRA | AR: 21 ±1 PR: 20 ±1 | AR: 179 ±3 PR: 181 ±4 | AR: 77 ±8 PR: 74 ±9 | AR: 59 ±8 PR: 59 ±4 |  | C | PAG | HIIT outdoors on a tartan track until exhaustion |
| Al Haddad et al. [98] | 11 | NR | REC | 22 ±3 | 178 ±6 | 74 ±8 | 52 ±3 |  | NC | CRO | HIIT indoors on a motorised treadmill until exhaustion |
| Alves et al. [120] | 27 | NR | REC | 22 ±3 | NR | NR | NR |  | NC | CRO | HIIT indoors on a motorised treadmill |
| Arantes et al. [8] | 11 | END | TRA | 25 ±4 | 174 ±9 | 65 ±8 | 56 ±1 |  | NC | CRO | HIIT indoors on motorised treadmill until exhaustion |
| Billat et al. [30] | 8 | END | TRA | 34 ±6 | 175 ±5 | 69 ±4 | 56 ±5 |  | NC | CRO | HIIT outdoors on a synthetic track until exhaustion |
| Bok et al. [20] | 20 | MIX | REC | 22 ±1 | 176 ±9 | 74 ±13 | 53 ±6 |  | NC | CRO | HIIT indoors – surface NR. |
| Bok et al. [46] | 17 | MIX | REC | 24 ±1 | 180 ±6 | 78 ±8 | 54 ±3 |  | NC | CRO | HIIT indoors on a motorised treadmill |
| Buchheit et al. [121] | 13 | MIX | TRA | 24 ±4 | 179 ±16 | 75 ±7 | 54 ±3 |  | NC | CRO | HIIT outdoors on a synthetic track until exhaustion |
| Buchheit et al. [45] | 9 | HB | NAT | 21 ±3 | 181 ±5 | 78 ±7 | 57 ±6 |  | NC | CRO | NR |
| Chambion-Diaz et al. [122] | 26 | NR | TRA | M:26  F: 26 | M:177  F: 162 | M:75  F:59 | M: 68  F: 52 |  | NC | CRO | HIIT indoors on a motorised treadmill (normoxia at 100% body weight condition) |
| Cipryan et al. [31] | 12 | NR | TRA | 23 ±2 | 184 ±8 | 77 ±8 | 57 ±6 |  | NC | CRO | HIIT indoor on a motorised treadmill |
| Cipryan et al. [32] | 16 | END/SPR | NAT | END: 22 ±3 SPR: 23 ±3 | END: 179 ±6 SPR: 182 ±6 | END: 70 ±7 SPR: 78 ±9 | END: 66 ±5  SPR: 57 ±5 |  | NC | CRO | HIIT outdoors on a motorised treadmill. Results separated by athlete sport. |
| Da Silva et al. [123] | 17 | SOC | NAT | 18 ±1 | 179 ±5 | 74 ±7 | 54 ±6 |  | NC | PAG | Data from 5 HIIT sessions during the preseason |
| Fleckenstein et al. [103] | 12 | END | NAT | M: 24 ±3  F: 20 ±2 | NR | NR | 59 ±5 |  | NC | CRO | HIIT indoors on a motorised treadmill |
| Julio et al. [70] | 21 | RUG/END | TRA | RUG: 22±3  END: 25 ±8 | RUG: 174 ±9  END: 177 ±7 | RUG: 78 ±7  END: 70 ±7 | RUG: 52 ±4  END: 70 ±7 |  | NC | CRO | HIIT on a track until exhaustion. Results separated by athlete sport. |
| Dellal et al. [21] | 22 | SOC | INT | 24 ±4 | 178 ±6 | 80 ±6 | NR |  | NC | CRO | HIIT outdoors on natural grass |
| Dellal et al. [22] | 10 | SOC | NAT | 24 ±3 | 180 ±7 | 76 ±6 | NR |  | NC | CRO | HIIT outdoors on artificial grass |
| Dellal et al. [129] | 22 | SOC | TRA | 26 ±5 | 180 ±4 | 79 ±6 | NR |  | NC | PAG | HIIT outdoor on artificial grass |
| Ducrocq et al. [33] | 13 | MIX | TRA | 21 ±2 | 173 ±9 | 67 ±9 | 55 ±7 |  | NC | CRO | HIIT indoors on a motorised treadmill |
| Dupont & Berthoin [39] | 12 | NR | TRA | 24 ±4 | 177 ±5 | 72 ±8 | 57 ±7 |  | NC | CRO | HIIT indoors on a tartan track until exhaustion |
| Dupont et al. [34] | 9 | NR | TRA | 25 ±3 | 179 ±6 | 74 ±6 | 54 ±7 |  | NC | CRO | HIIT indoors on a tartan track until exhaustion |
| McEwan et al. [12] | 14 | NR | REC | 30 ±7 | 180 ±5 | 79 ±9 | 54 ±8 |  | NC | CRO | HIIT indoors on a non-motorised treadmill. |
| McLean et al. [5] | 21 | ARF | TRA | 23 ±3 | 184 ±8 | 81 ±9 | NR |  | C | PAG | HIIT indoors on a motorised treadmill under normoxia |
| Midgley et al. [41] | 22 | END | TRA | 38 ±7 | 174 ±7 | 70 ±8 | NR |  | NC | CRO | HIIT indoors on motorised treadmill until exhaustion |
| Midgley et al. [40] | 13 | END | TRA | 39 ±9 | 174 ±6 | 71 ±7 | 60 ±6 |  | NC | CRO | HIIT indoors on motorised treadmill until exhaustion |
| Millet et al. [35] | 8 | END | NAT | 23 ±5 | 175 ±4 | 66 ±6 | 71 ±4 |  | NC | CRO | HIIT indoors on motorised treadmill until exhaustion |
| Millet et al. [101] | 7 | END | NAT | 21 ±3 | 174 ±4 | 65 ±6 | 71 ±4 |  | NC | CRO | HIIT indoors on motorised treadmill until exhaustion |
| Naves et al. [36] | 10 | NR | REC | 28 ±6 | 180 ±10 | 77 ±9 | 44 ±12 |  | NC | CRO | HIIT indoors on a motorised treadmill |
| Ortiz et al. [6] | 23 | SOC | NAT | 18 ±1 | 176 ±7 | 72 ±9 | NR |  | C | PAG | HIIT outdoor on natural grass during the preseason |
| Panascì et al. [13] | 15 | NR | REC | 22 ±1 | 176 ±6 | 66 ±7 | 56 ±5 |  | NC | CRO | HIIT session on an outdoor track and a second HIIT session on a motorised treadmill |
| Psarras et al. [23] | 16 | NR | REC | 20 ±2 | 180 ±7 | 76 ±6 | NR |  | NC | CRO | HIIT outdoors |
| Rozenek et al. [42] | 12 | WRE | TRA | 20 ±1 | 169 ±5 | 71 ±5 | 57 ±4 |  | NC | CRO | HIIT indoors on motorised treadmill |
| Seipp et al. [125] | 14 | HB | NAT | 26 ±2 | 191 ±8 | 95 ±12 | NR |  | NC | SG | HIIT on a HB court until exhaustion |
| Selmi et al. [126] | 20 | SOC | NAT | 24 ±1 | 179 ±8 | 74 ±7 | NR |  | NC | SG | HIIT outdoors on artificial grass |
| Selmi et al. [127] | 16 | SOC | NAT | 24 ±1 | 180 ±6 | 73 ±9 | NR |  | NC | CRO | HIIT outdoors on natural grass |
| Selmi et al. [128] | 16 | SOC | NAT | 18 ±1 | 178 ±6 | 70 ±5 | NR |  | NC | CRO | HIIT outdoors on natural grass |
| Tardieu-Berger et al. [43] | 11 | END | TRA | 17 ±1 | 177 ±7 | 70 ±7 | 63 ±4 |  | NC | CRO | HIIT outdoors on a tartan track until exhaustion |
| Thevenet et al. [37] | 8 | END | TRA | 16 ±1 | 171 ±4 | 58 ±5 | 57 ±6 |  | NC | CRO | HIIT outdoors on a tartan track until exhaustion |
| Thevenet et al. [9] | 9 | END | TRA | 17 ±1 | 158 ±7 | 63 ±8 | 65 ±4 |  | NC | CRO | HIIT outdoors on a tartan track until exhaustion |
| Thurlow et al. [11] | 24 | RL | TRA | 21 ±1 | 182 ±5 | 90 ±13 | NR |  | NC | PAG | HIIT on an outdoor natural grass field. Data from 6 preseason sessions |
| Wakefield & Glaister [44] | 7 | NR | REC | 22 ±5 | 182 ±6 | 86 ±11 | 51 ±5 |  | NC | CRO | HIIT indoors on a motorised treadmill until exhaustion |
| Zafeiridis et al. [100] | 10 | SOC | TRA | 21 ±2 | 175 ±4 | 71 ±5 | 58 ±4 |  | NC | CRO | HIIT indoors on a motorised treadmill |
| Zeng et al. [24] | 10 | BB/  HB | TRA | 20 ±1 | 165 ±6 | 57 ±11 | NR |  | NC | PAG | HIIT on a BB court during pre-season |
| Zeng et al. [7] | 19 | BB | TRA | 20 ±1 | 166 ±6 | 60 ±12 | NR |  | NC | PAG | HIIT on a BB court during pre-season |
| Zouhal et al. [38] | 7 | SOC | NAT | 20 ±5 | 175 ±4 | 68 ±9 | 58 ±6 |  | NC | CRO | HIIT outdoors on artifical grass |

Data are presented as mean ± standard deviation. Abbreviations: AR = active recovery group; ARF = Australian Rules football; BB = basketball; C = controlled study; CRO = crossover design; END = endurance running; HIIT = high-intensity interval training; HB = handball; INT = international; MIX = mixture of different sports; N = number of participants; NAT = national; NC = non-controlled study; NR = not reported; PAG = parallel groups design; PR = passive recovery group; SG = single group design; REC = recreational; RUG = rugby; SOC = soccer; SPR = sprint runners; TRA = trained; WRE = wrestling.

**Supplementary Table S7.** Summary of exercise protocol information and outcomes.

|  | **MAS Test** | | **HIIT protocol** | | | | | | **Outcomes** | |
| --- | --- | --- | --- | --- | --- | --- | --- | --- | --- | --- |
| **Study** | **Type** | **Prescribed Intensity (%)** | **Adjusted**  **Intensity (%)** | **Mode** | **Sets** × **Reps** | **Work:**  **Rest (s)** | **Rest Mode** | **I-set**  **Rest** |  |  |
| Abderrahman et al. [15] | GXT | 105 | 105 | STR | 1 × 22 | 30:30 | AR | - | Avg V̇O_2_:  T>95%V̇O_2max_:  T>90%V̇O_2max_:  T95/ET:  T90/ET: | 51.5 ±3.0 ml·min^-1^·kg^-1^  335 ±313 s  543 ±293 s  0.37 ±0.35  0.60 ±0.33 |
|  | GXT | 105 | 105 | STR | 1 × 22 | 30:30 | PR | - | Avg V̇O_2_:  T>95%V̇O_2max_:  T>90%V̇O_2max_:  T95/ET:  T90/ET: | 42.3 ±3.0 ml·min^-1^·kg^-1^  161 ±158 s  338 ±317 s  0.09 ±0.08  0.18 ±0.17 |
| Al Haddad et al. [98] | 30–15 | 95 | 120 | STR | 1 × 14* | 15:15 | AR | - | B[La]: | 13.9 ±2.4 mmol·L_­_^-1^ |
| Alves et al. [120] | GXT | 100 | 100 | STR | 1 × 20 | 30:30 | PR | - | sRPE: | 5.8 ±0.8 au |
| Arantes et al. [8] | GXT | 110 | 110 | STR | 1 × 24* | 30:30 | PR | - | Peak V̇O_2_:  T>90%V̇O_2max_:  Peak HR:  B[La]:  T90/ET: | 56.6 ±7.9 ml·min^-1^·kg^-1^  272 ±50 s  189 ±7 b·min^-1^  11.4 ±0.8 mmol·L_­_^-1^  0.19 ±0.03 |
|  | GXT | 110 | 110 | STR | 1 × 13* | 40:40 | PR | - | Peak V̇O_2_:  T>90%V̇O_2max_:  Peak HR:  B[La]:  T90/ET: | 57.6 ±7.6 ml·min^-1^·kg^-1^  284 ±72 s  189 ±10 b·min^-1^  7.4 ±1.8 mmol·L_­_^-1^  0.27 ±0.07 |
| Billat et al. [30] | GXT | 100 | 100 | STR | 1 × 19* | 30:30 | AR | **-** | Peak V̇O_2_:  T>95%V̇O_2max_:  T>90%V̇O_2max_:  T95/ET: | 61.1 ±6.5 ml·min^-1^·kg^-1^ & 102 ±4 %V̇O_2max_  471 ±398 s  338 ±317 s  0.41 ±0.35 |
| Bok et al. [20] | 20mSRT | 100 | 100 | SHU | 1 × 20 | 15:15 | PR | - | Avg V̇O_2_:  Peak V̇O_2_:  T>90%V̇O_2max_:  Avg HR:  Peak HR:  B[La]:  sRPE:  T90/ET: | 41.6 ±5.2 ml·min^-1^·kg^-1^ & 78 ±7 %V̇O_2max_  50.5 ±6.4 ml·min^-1^·kg^-1^ & 95 ±7 %V̇O_2max_  111 ±108 s  171 ±11 b·min^-1^ & 88 ±5 %HR_max_  183 ±10 b·min^-1^ & 94 ±4 %HR_max_  5.4 ±2.6 mmol·L_­_^-1^  6.7 ±1.0 au  0.19 ±0.18 |
|  | MSFT | 110 | 110 | SHU | 1 × 20 | 15:15 | PR | **-** | Avg V̇O_2_:  Peak V̇O_2_:  T>90%V̇O_2max_:  Avg HR:  Peak HR:  B[La]:  sRPE:  T90/ET: | 45.2 ±5.9 ml·min^-1^·kg^-1^ & 84 ±6 %V̇O_2max_  53.8 ±6.8 ml·min^-1^·kg^-1^ & 101 ±5 %V̇O_2max_  202 ±141 s  182 ±9 b·min^-1^ & 94 ±3 %HR_max_  192 ±10 b·min^-1^ & 99 ±2 %HR_max_  12.4 ±2.1 mmol·L_­_^-1^  9.5 ±0.5 au  0.34 ±0.24 |
| Bok et al. [46] | GXT | 110 | 110 | STR | 1 × 20 | 15:15 | PR | **-** | Avg V̇O_2_:  Peak V̇O_2_:  T>90%V̇O_2max_:  Avg HR:  Peak HR:  B[La]:  sRPE:  T90/ET: | 45.2 ±3.0 ml·min^-1^·kg^-1^  55.1 ±5.1 ml·min^-1^·kg^-1^  155 ±76 s  177 ±8 b·min^-1^  187 ±7 b·min^-1^  7.3 ±2.3 mmol·L_­_^-1^  8.2 ±1.4 au  0.26 ±0.13 |
|  | ASR | 15 | NA | STR | 1 × 20 | 15:15 | PR | **-** | Avg V̇O_2_:  Peak V̇O_2_:  T>90%V̇O_2max_:  Avg HR:  Peak HR:  B[La]:  sRPE:  T90/ET: | 42.7 ±2.9 ml·min^-1^·kg^-1^  55.8 ±4.4 ml·min^-1^·kg^-1^  178 ±85 s  178 ±7 b·min^-1^  188 ±8 b·min^-1^  8.2 ±1.0 mmol·L_­_^-1^  8.3 ±1.3 au  0.30 ±0.14 |
|  | ASR | 25 | NA | STR | 1 × 20 | 15:15 | PR | **-** | Avg V̇O_2_:  Peak V̇O_2_:  T>90%V̇O_2max_:  Avg HR:  Peak HR:  B[La]:  sRPE:  T90/ET: | 44.5 ±3.7 ml·min^-1^·kg^-1^  56.0 ±4.4 ml·min^-1^·kg^-1^  171 ±81 s  180 ±7 b·min^-1^  191 ±9 b·min^-1^  12.0 ±1.0 mmol·L_­_^-1^  9.8 ±0.4 au  0.29 ±0.14 |
| Buchheit et al. [121] | 30:15 | 90 | 115 | SHU | 1 × 40* | 15:15 | PR | **-** | Peak HR:  B[La]: | 90 ±4 %HR_max_  6.1 ±3.6 mmol·L_­_^-1^ |
|  | 30:15 | 95 | 120 | SHU | 1 × 17* | 15:15 | PR | **-** | Peak HR:  B[La]: | 92 ±4 %HR_max_  9.5 ±2.6 mmol·L_­_^-1^ |
|  | 30:15 | 100 | 125 | SHU | 1 × 7* | 15:15 | PR | **-** | Peak HR:  B[La]: | 92 ±3 %HR_max_  10.3 ±1.8 mmol·L_­_^-1^ |
|  | 30:15 | 90 | 115 | SHU | 1 × 13* | 30:30 | PR | **-** | Peak HR:  B[La]: | 90 ±4 %HR_max_  6.1 ±3.6 mmol·L_­_^-1^ |
|  | 30:15 | 95 | 120 | SHU | 1 × 5* | 30:30 | PR | **-** | Peak HR:  B[La]: | 93 ±4 %HR_max_  9.7 ±4.8 mmol·L_­_^-1^ |
|  | 30:15 | 100 | 125 | SHU | 1 × 3* | 30:30 | PR | **-** | Peak HR:  B[La]: | 93 ±3 %HR_max_  12.2 ±3.7 mmol·L_­_^-1^ |
| Buchheit et al. [45] | 30:15 | 95 | 120 | SHU | 1 × 16 | 15:15 | PR | **-** | Avg V̇O_2_:  Peak V̇O_2_:  Avg HR:  Peak HR:  B[La]  sRPE: | 50.1 ±6.1 ml·min^-1^·kg^-1^  56.4 ±6.4 ml·min^-1^·kg^-1^  179 ±7 b·min^-1^  189 ±7 6·min^-1^  11.6 ±3.3 mmol·L_­_^-1^  6.6 ±1.5 au |
| Chambion-Diaz et al. [122] | GXT | 100 | 100 | STR | 3 × 8 | 30:30 | PR | 3 min, PR | Males  Avg HR:  B[La]:  sRPE:  Females  Avg HR:  B[La]:  sRPE: | 156 ±14 b·min^-1^ & 81 ±5  2.7 ±0.8 mmol·L_­_^-1^  4.5 ±0.8 au  159 ±13 b·min^-1^ & 84 ±6  3.2 ±1.7 mmol·L_­_^-1^  3.4 ±0.9 au |
| Cipryan et al. [31] | GXT | 100 | 100 | STR | 1 × 24 | 15:15 | AR | **-** | Avg V̇O_2_:  Avg HR:  Peak HR:  sRPE: | 50.2 ±4.2 ml·min^-1^·kg^-1^  183 ±7 b·min^-1^  191 ±8 b·min^-1^  7.3 ±0.6 au |
|  | GXT | 100 | 100 | STR | 1 × 12 | 30:30 | AR | **-** | Avg V̇O_2_:  Avg HR:  Peak HR:  sRPE: | 49.8 ±3.7 ml·min^-1^·kg^-1^  180 ±6 b·min^-1^  188 ±6 b·min^-1^  6.3 ±0.7 au |
| Cipryan et al. [32] | GXT | 100 | 100 | STR | 1 × 21 | 30:30 | PR | - | END group  Avg V̇O_2_:  Avg HR:  sRPE:  SPR group  Avg V̇O_2_:  Avg HR:  sRPE: | 39.4 ±2.3 ml·min^-1^·kg^-1^ & 60 ±4 %V̇O_2max_  158 ±14 b·min^-1^ & 80 ±5 %HR_max_  3.1 ±0.4 au  35.1 ±2.5 ml·min^-1^·kg^-1^ & 62 ±6 %V̇O_2max_  158 ±18 b·min^-1^ & 80 ±7 %HR_max_  3.1 ±0.3 au |
| Da Silva et al. [123] | T-Car | 100 | 100 | SHU | 4 × 10 | 12:12 | PR | 3 min PR | Avg HR: | 92 ±2 %HR_max_ |
|  | T-Car | 100 | 100 | SHU | 4 × 10 | 12:12 | PR | 3 min PR | Avg HR: | 92 ±3 %HR_max_ |
|  | T-Car | 100 | 100 | SHU | 4 × 10 | 12:12 | PR | 3 min PR | Avg HR: | 90 ±4 %HR_max_ |
|  | T-Car | 100 | 100 | SHU | 4 × 10 | 12:12 | PR | 3 min PR | Avg HR: | 91 ±2 %HR_max_ |
|  | T-Car | 100 | 100 | SHU | 4 × 10 | 12:12 | PR | 3 min PR | Avg HR: | 90 ±5 %HR_max_ |
| Delextrat et al. [103] | 30–15 | 95 | 120 | STR | 2 × 16 | 15:15 | AR | NR | sRPE: | 8.0 ±0.5 au |
|  | 30–15 | 95 | 120 | STR | 2 × 18 | 15:15 | AR | NR | sRPE: | 7.4 ±0.8 au |
|  | 30–15 | 95 | 120 | STR | 2 × 20 | 15:15 | AR | NR | sRPE: | 7.5 ±0.4 au |
|  | 30–15 | 95 | 120 | STR | 2 × 22 | 15:15 | AR | NR | sRPE: | 8.2 ±0.7 au |
|  | 30–15 | 95 | 120 | STR | 2 × 26 | 15:15 | AR | NR | sRPE: | 8.1 ±1.0 au |
|  | 30–15 | 95 | 120 | STR | 2 × 18 | 15:15 | AR | NR | sRPE: | 7.5 ±0.5 au |
|  | 30–15 | 95 | 120 | STR | 2 × 20 | 15:15 | AR | NR | Avg HR: | 91 ±2 %HR_max_ |
| Dellal et al. [21] | VAM | 100 | 89 | SHU | 2 × 10 | 30:30 | AR | 4 min  PR | Peak HR: | 206 ±5 & 99 ±2 %HR_max_ |
|  | VAM | 100 | 89 | STR | 2 × 10 | 30:30 | AR | 4 min  PR | Peak HR: | 185 ±5 & 89 ±3 %HR_max_ |
|  | VAM | 105 | 94 | STR | 2 × 10 | 30:30 | AR | 4 min  PR | Peak HR: | 190 ±5 & 92 ±3 %HR_max_ |
|  | VAM | 110 | 99 | STR | 2 × 10 | 30:30 | AR | 4 min  PR | Peak HR: | 198 ±6 & 95 ±2 %HR_max_ |
|  | VAM | 110 | 99 | STR | 2 × 8 | 15:15 | PR | 5 min  PR | Peak HR: | 187 ±7 & 90 ±2 %HR_max_ |
|  | VAM | 115 | 106 | STR | 2 × 8 | 15:15 | PR | 5 min  PR | Peak HR: | 195 ±6 & 94 ±3 %HR_max_ |
|  | VAM | 115 | 106 | SHU | 2 × 8 | 15:15 | PR | 5 min  PR | Peak HR: | 205 ±6 & 99 ±1 %HR_max_ |
|  | VAM | 120 | 109 | STR | 2 × 6 | 10:10 | PR | 5 min  PR | Peak HR: | 194 ±9 & 94 ±2 %HR_max_ |
| Dellal et al. [22] | UMTT | 100 | 100 | SHU | 1 × 12 | 30:30 | AR | - | B[La]:  sRPE: | 7.4 ±1.3 mmol·L_­_^-1^  7.2 ±0.6 au |
|  | UMTT | 105 | 105 | SHU | 1 × 12 | 30:30 | AR | - | B[La]:  sRPE: | 9.0 ±2.1 mmol·L_­_^-1^  9.1 ±0.6 au |
|  | UMTT | 110 | 110 | SHU | 1 × 12 | 30:30 | AR | - | B[La]:  sRPE: | 10.9 ±1.5 mmol·L_­_^-1^  10.0 ±0.0 au |
|  | UMTT | 105 | 105 | SHU | 1 × 20 | 15:15 | PR | - | B[La]:  sRPE: | 9.8 ±2.5 mmol·L_­_^-1^  8.2 ±0.7 au |
|  | UMTT | 110 | 110 | SHU | 1 × 20 | 15:15 | PR | - | B[La]:  sRPE: | 10.9 ±3.1 mmol·L_­_^-1^  9.3 ±0.5 au |
|  | UMTT | 115 | 115 | SHU | 1 × 20 | 15:15 | PR | - | B[La]:  sRPE: | 12.5 ±2.0 mmol·L_­_^-1^  9.9 ±0.3 au |
|  | UMTT | 110 | 110 | SHU | 1 × 21 | 10:10 | PR | - | B[La]:  sRPE: | 11.4 ±2.3 mmol·L_­_^-1^  9.2 ±0.5 au |
|  | UMTT | 115 | 115 | SHU | 1 × 21 | 10:10 | PR | - | B[La]:  sRPE: | 12.1 ±2.6 mmol·L_­_^-1^  9.7 ±0.3 au |
|  | UMTT | 130 | 130 | SHU | 1 × 21 | 10:10 | PR | - | B[La]:  sRPE: | 13.1 ±2.3 mmol·L_­_^-1^  10.0 ±0.0 au |
|  | UMTT | 100 | 100 | STR | 1 × 12 | 30:30 | AR | - | B[La]:  sRPE: | 6.0 ±1.6 mmol·L_­_^-1^  4.8 ±1.5 au |
|  | UMTT | 105 | 105 | STR | 1 × 12 | 30:30 | AR | - | B[La]:  sRPE: | 6.5 ±1.5 mmol·L_­_^-1^  6.1 ±1.4 au |
|  | UMTT | 110 | 110 | STR | 1 × 12 | 30:30 | AR | - | B[La]:  sRPE: | 10.1 ±2.4 mmol·L_­_^-1^  7.8 ±1.2 au |
|  | UMTT | 105 | 105 | STR | 1 × 20 | 15:15 | PR | - | B[La]:  sRPE: | 8.0 ±1.8 mmol·L_­_^-1^  6.8 ±1.0 au |
|  | UMTT | 110 | 110 | STR | 1 × 20 | 15:15 | PR | - | B[La]:  sRPE: | 8.3 ±2.3 mmol·L_­_^-1^  7.0 ±1.0 au |
|  | UMTT | 115 | 115 | STR | 1 × 20 | 15:15 | PR | - | B[La]:  sRPE: | 10.0 ±2.7 mmol·L_­_^-1^  8.1 ±10.7 au |
|  | UMTT | 110 | 110 | STR | 1 × 21 | 10:10 | PR | - | B[La]:  sRPE: | 8.7 ±203 mmol·L_­_^-1^  7.8 ±1.1 au |
|  | UMTT | 115 | 115 | STR | 1 × 21 | 10:10 | PR | - | B[La]:  sRPE: | 10.0 ±1.7 mmol·L_­_^-1^  8.3 ±0.6 au |
|  | UMTT | 130 | 130 | STR | 1 × 21 | 10:10 | PR | - | B[La]:  sRPE: | 11.2 ±2.4 mmol·L_­_^-1^  9.2 ±0.6 au |
| Dellal et al. [124] | 30–15 | 95 | 120 | SHU | 2 × 10 | 30:30 | PR | 6 min  PR | sRPE: | 8.0 ±1.1 au |
|  | 30–15 | 95 | 120 | SHU | 2 × 10 | 30:30 | PR | 6 min  PR | sRPE: | 8.0 ±0.9 au |
|  | 30–15 | 95 | 120 | SHU | 2 × 10 | 30:30 | PR | 6 min  PR | sRPE: | 7.4 ±0.5 au |
|  | 30–15 | 100 | 125 | SHU | 2 × 8 | 15:15 | PR | 5 min  PR | sRPE: | 8.0 ±0.5 au |
|  | 30–15 | 100 | 125 | SHU | 2 × 8 | 15:15 | PR | 5 min  PR | sRPE: | 8.1 ±0.4 au |
|  | 30–15 | 100 | 125 | SHU | 2 × 8 | 15:15 | PR | 5 min  PR | sRPE: | 7.6 ±0.5 au |
|  | 30–15 | 95 | 120 | SHU | 2 × 7 | 10:10 | PR | 5 min  PR | sRPE: | 7.3 ±0.5 au |
|  | 30–15 | 95 | 120 | SHU | 2 × 7 | 10:10 | PR | 5 min  PR | sRPE: | 7.6 ±0.5 au |
|  | 30–15 | 95 | 120 | SHU | 2 × 7 | 10:10 | PR | 5 min  PR | sRPE: | 7.6 ±0.5 au |
| Ducroq et al. [33] | GXT | 120 | 120 | STR | 1 × 22 | 15:15 | PR | - | Peak V̇O_2_:  T>90%V̇O_2max_:  Peak HR:  B[La]:  sRPE:  T90/ET: | 54.3 ±6.7 ml·min^-1^·kg^-1^  145 ±76 s  182 ±11 b·min^-1^  7.4 ±2.2 mmol·L_­_^-1^  6.4 ±2.2 au  0.22 ±0.12 |
| Dupont & Berthoin [39] | GXT | 120 | 120 | STR | 1 × 15^*^ | 15:15 | AR | - | Avg V̇O_2_:  Peak V̇O_2_:  T>90%V̇O_2max_:  T>95%V̇O_2max_:  Avg HR:  Peak HR:  B[La]:  T90/ET:  T95/ET: | 51.6 ±6.6 ml·min^-1^·kg^-1^  61.6 ±8.9 ml·min^-1^·kg^-1^  282 ±117 s  180 ±127 s  174 ±9 b·min^-1^  185 ±9 b·min^-1^  10.7 ±2.0 mmol·L_­_^-1^  0.63 ±0.26  0.40 ±0.28 |
|  | GXT | 120 | 120 | STR | 1 × 25^*^ | 15:15 | PR | - | Avg V̇O_2_:  Peak V̇O_2_:  T>90%V̇O_2max_:  T>95%V̇O_2max_:  Avg HR:  Peak HR:  B[La]:  T90/ET:  T95/ET: | 49.4 ±6.8 ml·min^-1^·kg^-1^  60.8 ±6.9 ml·min^-1^·kg^-1^  317 ±132 s  191 ±135 s  176 ±8 b·min^-1^  185 ±8 b·min^-1^  11.7 ±2.1 mmol·L_­_^-1^  0.42 ±0.18  0.25 ±0.18 |
| Dupont et al. [34] | GXT | 110 | 110 | STR | 1 × 47* | 15:15 | PR | - | Peak V̇O_2_:  T>90%V̇O_2max_:  T>95%V̇O_2max_:  Peak HR:  B[La]:  T90/ET:  T95/ET: | 54.7 ±6.0 ml·min^-1^·kg^-1^  383 ±180 s  120 ±42 s  187 ±11 b·min^-1^  11.1 ±2.5 mmol·L_­_^-1^  0.27 ±0.13  0.09 ±0.03 |
|  | GXT | 120 | 120 | STR | 1 × 23* | 15:15 | PR | - | Peak V̇O_2_:  T>90%V̇O_2max_:  T>95%V̇O_2max_:  Peak HR:  B[La]:  T90/ET:  T95/ET: | 56.0 ±5.6 ml·min^-1^·kg^-1^  323 ±272 s  202 ±66 s  193 ±13 b·min^-1^  13.1 ±3.7 mmol·L_­_^-1^  0.47 ±0.39  0.29 ±0.10 |
|  | GXT | 130 | 130 | STR | 1 × 11* | 15:15 | PR | - | Peak V̇O_2_:  T>90%V̇O_2max_:  T>95%V̇O_2max_:  Peak HR:  B[La]:  T90/ET:  T95/ET | 55.0 ±6.5 ml·min^-1^·kg^-1^  135 ±133 s  50 ±47 s  193 ±9 b·min^-1^  14.7 ±4.9 mmol·L_­_^-1^  0.41 ±0.40  0.15 ±0.14 |
|  | GXT | 140 | 140 | STR | 1 × 6* | 15:15 | PR | - | Peak V̇O_2_:  T>90%V̇O_2max_:  T>95%V̇O_2max_:  Peak HR:  B[La]:  T90/ET:  T95/ET: | 52.2 ±3.6 ml·min^-1^·kg^-1^  77 ±96 s  48 ±59 s  189 ±7 b·min^-1^  13.5 ±2.9 mmol·L_­_^-1^  0.44 ±0.53  0.27 ±0.33 |
| Fleckenstein et al. [102] | GXT | 100 | 100 | STR | 1 × 24 | 30:30 | AR | - | Avg V̇O_2_:  Peak V̇O_2_:  T>90%V̇O_2max_:  T>95%V̇O_2max_:  Avg HR:  Peak HR:  B[La]:  sRPE:  T90/ET:  T95/ET: | 78 ±4%  91 ±4%  201 ±268 s  58 ±139 s  90 ±2%  96 ±2%  7.6 ±2.0 mmol·L_­_^-1^  6.5 ±1.0 au  0.14 ±0.19  0.04 ±0.10 |
| Julio et al. [70] | GXT | 110 | 110 | STR | 1 × 12* | 15:15 | PR | - | END group  B[La]:  sRPE  RUG group  B[La]:  sRPE | 9.0 ±3.2 mmol·L_­_^-1^  7.2 ±1.0 au  6.2 ±2.8 mmol·L_­_^-1^  8.4 ±1.3 au |
|  | ASR | 25% | NA | STR | 1 × 6* | 15:15 | PR | - | END group  B[La]:  sRPE  RUG group  B[La]:  sRPE | 9.7 ±1.8 mmol·L_­_^-1^  7.7 ±1.0 au  10.2 ±2.6 mmol·L_­_^-1^  8.6 ±1.0 au |
|  | ASR | 50% | NA | STR | 1 × 2* | 15:15 | PR |  | END group  B[La]:  sRPE  RUG group  B[La]:  sRPE | 11.1 ±3.1 mmol·L_­_^-1^  8.2 ±0.9 au  10.1 ±2.1 mmol·L_­_^-1^  8.5 ±0.7 au |
| McEwan et al. [12] | GXT | 105 | 105 | STR | 1 × 12* | 30:30 | PR | - | Avg HR:  Peak HR:  B[La]:  sRPE: | 166 ±9 b·min^-1^ & 90 ±3 %HR_max_  178 ±8 b·min^-1^ & 97 ±2 %HR_max_  9.4 ±3.2 mmol·L_­_^-1^  8.7 ±11 au |
| McLean et al. [5] | GXT | 104 | 104 | STR | 1 × 8 | 15:15 | PR | - | sRPE: | 5.7 ±1.8 au |
|  | GXT | 110 | 110 | STR | 1 × 10 | 15:15 | PR | - | sRPE: | 5.9 ±1.3 au |
| Midgley et al. [41] | GXT | 105 | 105 | STR | 1 × 14* | 30:30 | AR | - | T>90%V̇O_2max_:  T>95%V̇O_2max_:  T90/ET:  T95/ET: | 480 ±220 s  232 ±174 s  0.56 ±0.26  0.27 ±0.20 |
|  | GXT | 105 | 105 | STR | 1 × 15* | 30:30 | AR | - | T>90%V̇O_2max_:  T>95%V̇O_2max_:  T90/ET:  T95/ET: | 488 ±252 s  244 ±195 s  0.53 ±0.27  0.26 ±0.21 |
| Midgley et al. [40] | GXT | 100 | 100 | STR | 1 × 21* | 30:30 | AR | - | Peak V̇O_2_:  Peak HR:  B[La]: | 55.8 ±6.3 ml·min^-1^·kg^-1^  174 ±10 b·min^-1^  4.9 ±1.7 mmol·L_­_^-1^ |
| Millet et al. [35] | GXT | 100 | 100 | STR | 3 × 8* | 30:30 | AR | 5 min  PR | T>90%V̇O_2max_:  T>95%V̇O_2max_:  Peak HR:  sRPE:  T90/ET:  T95/ET: | 149 ±133 s  54 ±50 s  183 ±9 b·min^-1^  3.2 ±0.1 au  0.10 ±0.09  0.04 ±0.03 |
| Millet et al. [101] | GXT | 100 | 100 | STR | 3 × 8* | 30:30 | AR | 5 min  PR | Peak V̇O_2_:  T>90%V̇O_2max_:  Peak HR:  sRPE:  T90/ET: | 66.2 ±4.6 ml·min^-1^·kg^-1^  168 ±131 s  186 ±4 b·min^-1^  4.6 ±0.4 au  0.12 ±0.09 |
|  | GXT | 100 | 105 | STR | 3 × 8* | 30:30 | AR | 5 min  PR | Peak V̇O_2_:  T>90%V̇O_2max_:  Peak HR:  sRPE:  T90/ET: | 71.1 ±4.9 ml·min^-1^·kg^-1^  338 ±149 s  192 ±7 b·min^-1^  4.6 ±0.4 au  0.23 ±0.10 |
| Naves et al. [36] | GXT | 100 | 100 | STR | 1 × 29 | 30:30 | PR | - | Peak V̇O_2_:  Peak HR:  sRPE: | 32.0 ±7.6 ml·min^-1^·kg^-1^  168 ±8 b·min^-1^  4.1 ±1.1 au |
| Ortiz et al. [6] | T-Car | 115 | 115 | SHU | 1 × 5 | 12:12 | PR | - | Avg HR:  B[La]: | 91 ±2 %HR_max_  5.8 ±1.0 mmol·L_­_^-1^ |
|  | T-Car | 115 | 115 | SHU | 2 × 5 | 12:12 | PR | 3 min  PR | Avg HR:  B[La]: | 95 ±5 %HR_max_  9.2 ±2.1 mmol·L_­_^-1^ |
|  | T-Car | 115 | 115 | SHU | 3 × 5 | 12:12 | PR | 3 min  PR | Avg HR:  B[La]: | 92 ±4 %HR_max_  10.7 ±2.3 mmol·L_­_^-1^ |
|  | T-Car | 115 | 115 | SHU | 4 × 5 | 12:12 | PR | 3 min  PR | Avg HR:  B[La]: | 96 ±4 %HR_max_  11.5 ±2.4 mmol·L_­_^-1^ |
|  | T-Car | 115 | 115 | SHU | 5 × 5 | 12:12 | PR | 3 min  PR | Avg HR:  B[La]: | 91 ±5 %HR_max_  11.4 ±2.3 mmol·L_­_^-1^ |
|  | T-Car | 115 | 115 | SHU | 6 × 5 | 12:12 | PR | 3 min  PR | Avg HR:  B[La]: | 94 ±3 %HR_max_  12.3 ±2.0 mmol·L_­_^-1^ |
| Panasci et al. [13] | 30–15 | 100 | 125 | STR | 1 × 15 | 30:30 | PR | - | Avg V̇O_2_:  Peak V̇O_2_:  T>90%V̇O_2max_:  T90/ET: | 45.6 ±5.4 ml·min^-1^·kg^-1^ & 82 ±9%V̇O_2max_  51.7 ±1.6 ml·min^-1^·kg^-1^ & 92 ±3%V̇O_2max_  348 ±286 s  0.39 ±0.31 |
|  | 30–15 | 100 | 125 | STR | 1 × 15 | 30:30 | PR | - | Avg V̇O_2_:  Peak V̇O_2_:  T>90%V̇O_2max_:  T90/ET: | 43.2 ±6.2 ml·min^-1^·kg^-1^ & 77 ±8%V̇O_2max_  48.0 ±1.1 ml·min^-1^·kg^-1^ & 86 ±2%V̇O_2max_  348 ±283 s  0.39 ±0.31 |
|  | 30–15 | 115 | 140 | STR | 1 × 15 | 30:30 | PR | - | Avg V̇O_2_:  Peak V̇O_2_:  T>90%V̇O_2max_:  T90/ET: | 47.6 ±5.6 ml·min^-1^·kg^-1^ & 85 ±11%V̇O_2max_  51.0 ±0.1 ml·min^-1^·kg^-1^ & 91 ±2%V̇O_2max_  326 ±89 s  0.36 ±0.10 |
| Psarras et al. [23] | 20mSRT | 100 | 86 | STR | 2 × 12 | 15:15 | PR | - | Avg HR:  Peak HR:  B[La]: | 151 ±14 b·min^-1^  163 ±15 b·min^-1^  4.5 ±2.5 mmol·L_­_^-1^ |
|  | 20mSRT | 100 | 85 | STR | 2 × 12 | 15:15 | PR | - | Avg HR:  Peak HR:  B[La]: | 160 ±10 b·min^-1^  169 ±14 b·min^-1^  4.6 ±2.7 mmol·L_­_^-1^ |
| Rozenek et al. [42] | GXT | 100 | 100 | STR | 1 × 35* | 15:15 | AR | - | Avg V̇O_2_:  Peak V̇O_2_:  T>90%V̇O_2max_:  Avg HR:  Peak HR:  B[La]:  sRPE: | 72 ±4%V̇O_2max_  78 ±4%V̇O_2max_  0 ±0 s  85 ±4%HR_max_  90 ±5%HR_max_  7.3 ±2.4 mmol·L_­_^-1^  6.4 ±1.2 au |
| Seipp et al. [125] | 30–15 | 90 | 115 | SHU | 1 × 13* | 15:15 | PR | - | B[La]:  sRPE: | 8.8 ±1.0 mmol·L_­_^-1^  8.4 ±0.8 au |
| Selmi et al. [126] | YYIR1 | 120 | 120 | STR | 4 × 12 | 10:10 | PR | 3 min  PR | sRPE: | 7.0 ±1.0 au |
| Selmi et al. [127] | VAM | 110 | 99 | STR | 4 × 8 | 15:15 | PR | 3 min  PR | sRPE: | 7.1 ±1.0 au |
| Selmi et al. [128] | VAM | 110 | 99 | STR | 4 × 8 | 15:15 | PR | 3 min  PR | sRPE: | 7.1 ±1.0 au |
| Tardieu-Berger et al. [43] | UMTT | 110 | 110 | STR | 1 × 10* | 30:30 | AR | - | Avg V̇O_2_:  Peak V̇O_2_:  T>90%V̇O_2max_:  B[La]:  T90/ET: | 90 ±5%V̇O_2max_  97 ±5%V̇O_2max_  345 ±204 s  10.7 ±0.0 mmol·L_­_^-1^  0.58 ±0.34 |
|  | UMTT | 110 | 110 | STR | 3 × 6* | 30:30 | AR | 4 min  AR | Avg V̇O_2_:  Peak V̇O_2_:  T>90%V̇O_2max_:  B[La]:  T90/ET: | 85 ±9%V̇O_2max_  94 ±10%V̇O_2max_  290 ±280 s  11.5 ±3.3 mmol·L_­_^-1^  0.30 ±0.29 |
| Thevenet et al. [37] | GXT | 105 | 105 | STR | 1 × 18* | 30:30 | AR | - | T>90%V̇O_2max_:  T90/ET | 746 ±417 s  0.69 ±0.39 |
|  | GXT | 105 | 105 | STR | 1 × 7* | 30:30 | AR | - | T>90%V̇O_2max_:  T90/ET | 306 ±85 s  0.73 ±0.20 |
|  | GXT | 105 | 105 | STR | 1 × 12* | 30:30 | AR | - | T>90%V̇O_2max_:  T90/ET | 587 ±259 s  0.82 ±0.36 |
| Thevenet et al. [9] | GXT | 100 | 100 | STR | 1 × 24* | 30:30 | AR | - | T>90%V̇O_2max_:  T>95%V̇O_2max:_  B[La]:  T90/ET:  T95/ET: | 300 ±150 s  118 ±66 s  8.9 ±1.1  0.21 ±0.10  0.08 ±0.05 |
|  | GXT | 110 | 110 | STR | 1 × 11* | 30:30 | AR | - | T>90%V̇O_2max_:  T>95%V̇O_2max:_  B[La]:  T90/ET:  T95/ET: | 198 ±148 s  62 ±75 s  11.5 ±3.8  0.30 ±0.22  0.09 ±0.11 |
| Thurlow et al. | ASR | 25% | NA | STR | 2 × 6 | 15 | 15 | PR  2 min | sRPE: | 4.8 ±0.4 |
|  | ASR | 30% | NA | STR | 2 × 6 | 15 | 15 | PR  2 min | sRPE: | 5.1 ±0.4 |
|  | ASR | 25% | NA | STR | 2 × 8 | 15 | 15 | PR  2 min | sRPE: | 6.3 ±0.5 |
|  | ASR | 27.5% | NA | STR | 2 × 8 | 15 | 15 | PR  2 min | sRPE: | 6.0 ±0.4 |
|  | ASR | 27.5% | NA | STR | 2 × 6 | 15 | 15 | PR  2 min | sRPE: | 5.6 ±0.3 |
|  | ASR | 30% | NA | STR | 2 × 6 | 15 | 15 | PR  2 min | sRPE: | 7.3 ±0.5 |
| Wakefield & Glaister [44] | GXT | 105 | 105 | STR | 1 × 82* | 20:20 | PR | - | Avg HR:  B[La]:  T>95%V̇O_2max_:  T95/ET: | 163 ±7 b·min^-1^  2.6 ±1.6 mmol·L_­_^-1^  42 ±40 s  0.01 ±0.01 |
|  | GXT | 115 | 115 | STR | 1 × 59* | 20:20 | PR | - | Avg HR:  B[La]:  T>90%V̇O_2max_:  T95/ET | 167 ±8 b·min^-1^  4.3 ±2.0 mmol·L_­_^-1^  26 ±34 s  0.01 ±0.01 |
| Zafeiridis et al. [100] | GXT | 110 | 110 | STR | 1 × 9* | 30:30 | AR | - | Avg V̇O_2_:  Peak V̇O_2_:  T>90%V̇O_2max_:  T>95%V̇O_2max_:  Avg HR:  Peak HR:  B[La]:  T90/ET:  T95/ET: | 86 ±4 %V̇O_2max_  96 ±4 %V̇O_2max_  326 ±207 s  84 ±104 s  91 ±1 %HR_max_  97 ±1 %HR_max_  8.0 ±1.5 mmol·L_­_^-1^  0.59 ±0.38  0.15 ±0.19 |
| Zeng et al. [24] | 30–15 | 90 | 115 | STR | 1 × 12 | 15:15 | PR | - | Avg HR:  sRPE: | 173 ±8 b·min^-1^ & 87 ±4%HR_max_  7.1 ±0.5 au |
|  | 30–15 | 90 | 115 | STR | 1 × 14 | 15:15 | PR | - | Avg HR:  sRPE: | 179 ±9 b·min^-1^ & 90 ±3%HR_max_  7.5 ±0.3 au |
|  | 30–15 | 95 | 120 | STR | 1 × 16 | 15:15 | PR | - | Avg HR:  sRPE: | 180 ±7 b·min^-1^ & 91 ±2%HR_max_  7.9 ±0.5 au |
|  | 30–15 | 95 | 120 | STR | 1 × 18 | 15:15 | PR | - | Avg HR:  sRPE: | 182 ±9 b·min^-1^ & 92 ±2%HR_max_  7.9 ±0.4 au |
| Zeng et al. [7] | 30–15 | 90 | 115 | SHU | 1 × 12 | 15:15 | PR | - | Avg HR:  sRPE: | 173 ±8 b·min^-1^ & 87 ±4%HR_max_  7.1 ±0.5 au |
|  | 30–15 | 90 | 115 | SHU | 1 × 14 | 15:15 | PR | - | Avg HR:  sRPE: | 179 ±9 b·min^-1^ & 90 ±3%HR_max_  7.5 ±0.3 au |
|  | 30–15 | 95 | 120 | SHU | 1 × 16 | 15:15 | PR | - | Avg HR:  sRPE: | 180 ±7 b·min^-1^ & 91 ±2%HR_max_  7.9 ±0.5 au |
|  | 30–15 | 95 | 120 | SHU | 1 × 18 | 15:15 | PR | - | Avg HR:  sRPE: | 182 ±9 b·min^-1^ & 92 ±2%HR_max_  7.9 ±0.4 au |
| Zouhal et al. [38] | GXT | 120 | 120 | STR | 4 × 8 | 15:15 | PR | 3 min  AR | Avg V̇O_2_:  Peak V̇O_2_:  Avg HR:  Peak HR:  B[La]:  sRPE: | 36.8 ±1.9 ml·min^-1^·kg^-1^  53.3 ±3.32 ml·min^-1^·kg^-1^  156 ±9 b·min^-1^ & 70 ±3%HR_max_  187 ±8 b·min^-1^  8.4 ±2.6 mmol·L_­_^-1^  3.5 ±0.5 au |

Abbreviations: 20mSRT = multistage 20m shuttle run test; 30–15 = 30–15 intermittent fitness test; AR = active recovery group; Avg = average; ASR = anaerobic speed reserve; B[La] = blood lactate; GXT = graded exercise test with gas analysis; HIIT = high-intensity interval training; HR = heart rate; HR_max_ = maximal heart rate; I-set = inter-set; MAS = maximal aerobic speed; NA = not applicable; NR = not reported; PR = passive recovery; SHU = shuttle runs; sRPE = session ratings of perceived exertion; STR = straight-line runs; T>90%V̇O_2max_ = time above 90% of V̇O_2max_; T>95%V̇O_2max_ = time above 95% of V̇O_2max_; T90/ET = time above 90% of V̇O_2max_/exercise time ratio; T95/ET = time above 95% of V̇O_2max_/exercise time ratio; T-Car = Carminatti's Test; UMTT = University of Montreal Track Test; YYIR1 = Yo-Yo Intermittent Recovery Test Level 1; VAM = Vam-Eval test; V̇O_2_ = oxygen consumption; V̇O_2max_ = maximal oxygen consumption; * = repetitions performed until exhaustion

**Supplementary Table S8.** The moderating effects of athlete competitive level on the acute physiological and perceptual demands of short-bout HIIT. All effects are compared against the reference intercept for the recreational competitive level.

| **Outcome** | **Reference intercept (Estimate ±90% CL)** | **Comparison** | **Change ±90% CL** | **Practical interpretation** |
| --- | --- | --- | --- | --- |
| HR_avg_ (b·min^-1^) | 166; 159 to 174 | Trained | 7; -5 to 19 | ? |
|  |  | National | 4; -10 to 17 | ? |
| HR_peak_ (b·min^-1^) | 178; 172 to 183 | Trained | 8; 0 to 16 | **↑↑** |
|  |  | National | 10; 1 to 19 | **↑↑** |
| B[La] (mmol·L_­_^-1^) | 7.7; 5.7 to 9.7 | Trained | 2.0; -1.3 to 3.7 | ? |
|  |  | National | 0.0; -3.0 to 3.0 | ? |
| Avg V̇O_2_ (ml·min^-1^·kg^-1^) | 44.0; 40.0 to 48.0 | Trained | 5.1; -0.8 to 10.9 | ? |
|  |  | National | -1.8; -8.7 to 5.1 | ? |
| Peak V̇O_2_ (ml·min^-1^·kg^-1^) | 47.0; 39.1 to 54.9 | Trained | 10.2; 0.5 to 19.9 | **↑↑** |
|  |  | National | 15.6; 3.1 to 28.2 | **↑↑** |
| sRPE (CR10^®^ units) | 7.7; 6.7 to 8.8 | Trained | -0.6; -1.9 to 0.6 | =↓ |
|  |  | National | -1.2; -2.5 to 0.2 | =↓ |
| T>90%V̇O_2max_ (s) | 162; 17 to 307 | Trained | 126; -33 to 286 | ? |
|  |  | National | 44; -175 to 264 | ? |
| T>95%V̇O_2max_ (s) | 34; -92 to 159 | Trained | 114; -21 to 250 | ? |
|  |  | National | 20; -171 to 211 | ? |
| T90/ET | 0.27; 0.06 to 0.47 | Trained | 0.12; -0.10 to 0.34 | ? |
|  |  | National | -0.13; -0.42 to 0.17 | ? |
| T95/ET | 0.01; -0.15 to 0.18 | Trained | 0.17; 0.00 to 0.35 | **↑↑** |
|  |  | National | 0.03; -0.23 to 0.28 | ? |
| Abbreviations: Avg = average; B[La] = blood lactate; HR_avg_ = average heart rate; HR_peak_ = peak heart rate; sRPE = session ratings of perceived exertion; T>90%V̇O_2max_ = time above 90% of V̇O_2max_; T>95%V̇O_2max_ = time above 95% of V̇O_2max_; T90/ET = time above 90% of V̇O_2max_/exercise time ratio; T95/ET = time above 95% of V̇O_2max_/exercise time ratio  Indicates ‘↑↑’ substantial increase; ‘=↓’ indicates compatibility with a trivial to substantial decrease; ‘?’ indicates inconclusive. | | | | |

**Supplementary Table S9.** The moderating effects of athlete fitness level on the acute physiological and perceptual demands of short-bout HIIT. All effects are compared against the reference intercept for the 50−55 ml∙kg^-1^∙min^-1^ category.

| **Outcome** | **Reference intercept (Estimate ±90% CL)** | **Comparison** | **Change ±90% CL** | **Practical interpretation** |
| --- | --- | --- | --- | --- |
| HR_avg_ (b·min^-1^) | 170; 163 to 176 | V̇O_2max:_ 55–60 | 5; -4 to 14 | ? |
|  |  | V̇O_2max:_ 60–65 | -5; -14 to 4 | ? |
| HR_peak_ (b·min^-1^) | 186; 182 to 190 | V̇O_2max:_ 55–60 | 2; -4 to 8 | ? |
|  |  | V̇O_2max:_ 60–65 | -3; -10 to 3 | ? |
| B[La] (mmol·L_­_^-1^) | 8.9; 7.4 to 10.5 | V̇O_2max:_ 55–60 | 0.0; -2.3 to 2.3 | ? |
|  |  | V̇O_2max:_ 60–65 | -0.1; -1.9 to 2.1 | ? |
| Avg V̇O_2_ (ml·min^-1^·kg^-1^) | 43.2; 36.5 to 49.9 | V̇O_2max:_ 55–60 | 3.2; -3.6 to 10.1 | ? |
|  |  | V̇O_2max:_ 60–65 | 3.9 -5.3 to 13.1 | ? |
| Peak V̇O_2_ (ml·min^-1^·kg^-1^) | 54.0; 50.4 to 57.6 | V̇O_2max:_ 55–60  V̇O_2max:_ 60–65 | 5.0; -0.3 to 10.2  8.5; 2.2 to 14.8 | =**↑**  **↑↑** |
| sRPE (CR10^®^ units) | 7.9; 6.9 to 8.8 | V̇O_2max:_ 55–60 | -1.7; -2.9 to -0.4 | ↓↓ |
|  |  | V̇O_2max:_ 60–65 | -1.6; -2.7 to -0.6 | ↓↓ |
| T>90%V̇O_2max_ (s) | 174; 89 to 259 | V̇O_2max:_ 55–60 | 125; 9 to 240 | **↑↑** |
|  |  | V̇O_2max:_ 60–65 | 75; -54 to 203 | ? |
| T>95%V̇O_2max_ (s) | 74; 9 to 139 | V̇O_2max:_ 55–60 | 83; -8 to 174 | =**↑** |
|  |  | V̇O_2max:_ 60–65 | 2; -101 to 104 | ? |
| T90/ET | 0.28; 0.13 to 0.43 | V̇O_2max:_ 55–60 | 0.14; -0.06 to 0.33 | ? |
|  |  | V̇O_2max:_ 60–65 | -0.04; -0.26 to 0.17 | ? |
| T95/ET | 0.10; -0.03 to 0.24 | V̇O_2max:_ 55–60 | 0.10; -0.08 to 0.27 | ? |
|  |  | V̇O_2max:_ 60–65 | -0.04; -0.24 to 0.17 | ? |
| Abbreviations: Avg = average; B[La] = blood lactate; HR_avg_ = average heart rate; HR_peak_ = peak heart rate; sRPE = session ratings of perceived exertion; T>90%V̇O_2max_ = time above 90% of V̇O_2max_; T>95%V̇O_2max_ = time above 95% of V̇O_2max_; T90/ET = time above 90% of V̇O_2max_/exercise time ratio; T95/ET = time above 95% of V̇O_2max_/exercise time ratio  Indicates ‘↑↑’ substantial increase; indicates ‘↓↓’ substantial decrease; ‘?’ indicates inconclusive; =↑ compatible with a trivial to substantial increase | | | | |

| **Outcome Measure** | | **Number of…** | |  | **Pooled Effect** | | | |  | **Variation (*σ*; 90% CI) between…** | |
| --- | --- | --- | --- | --- | --- | --- | --- | --- | --- | --- | --- |
|  |  | **Studies** | **Samples** |  | **Estimate** |  | **90% CI** | **90% PI** |  | **Studies (*σ*_1_)** | **Samples (*σ*_2_)** |
| **B[La]** | mmol∙L^-1^ | 16 | 38 |  | 7.5 |  | 6.3 to 8.7 | 2.3 to 12.7 |  | 2.0 (1.0 to 3.3) | 2.1 (1.6 to 2.7) |
| **HR_avg_** | b∙min^-1^ | 12 | 22 |  | 170 |  | 165 to 175 | 151 to 188 |  | 10 (7 to 15) | 2 (1 to 5) |
|  | % HR_max_ | 12 | 22 |  | 88 |  | 86 to 90 | 81 to 95 |  | 3 (2 to 5) | 2 (1 to 3) |
| **HR_peak_** | b∙min^-1^ | 12 | 24 |  | 183 |  | 178 to 188 | 164 to 202 |  | 8 (4 to 14) | 6 (4 to 9) |
|  | %HR_max_ | 5 | 13 |  | 94 |  | 92 to 96 | 87 to 102 |  | 0 (0 to 3) | 3 (2 to 5) |
| **V̇O_2avg_** | ml∙kg^-1^∙min^-1^ | 8 | 17 |  | 46 |  | 43 to 49 | 36 to 55 |  | 4 (2 to 7) | 3 (2 to 4) |
|  | % V̇O_2max_ | 4 | 6 |  | 73 |  | 62 to 84 | 48 to 97 |  | 9 (4 to 22) | 3 (0 to 9) |
| **V̇O_2peak_** | ml∙kg^-1^∙min^-1^ | 7 | 11 |  | 53 |  | 46 to 60 | 33 to 73 |  | 10 (6 to 17) | 0 (0 to 2) |
|  | %V̇O_2max_ | 4 | 5 |  | 92 |  | 80 to 104 | 65 to 119 |  | 9 (0 to 24) | 4 (0 to 17) |
| **T>90% V̇O_2max_** | s | 7 | 12 |  | 191 |  | 93 to 290 | -77 to 460 |  | 128 (79 to 236) | 0 (0 to 3) |
| **T>95%**  **V̇O_2max_** | s | 4 | 6 |  | 183 |  | 48 to 319 | -85 to 452 |  | 99 (0 to 329) | 0 (0 to 3) |
| **T90/ET** |  | 6 | 11 |  | 0.30 |  | 0.20 to 0.40 | 0.01 to 0.60 |  | 0.07 (0.00 to 0.21) | 0.11 (0.04 to 0.22) |
| **T95/ET** |  | 4 | 6 |  | 0.24 |  | 0.07 to 0.40 | -0.16 to 0.63 |  | 0.09 (0.00 to 0.32) | 0.15 (0.04 to 0.31) |
| **sRPE** | (CR10^®^ units) | 19 | 63 |  | 7.1 |  | 6.7 to 7.6 | 4.7 to 9.5 |  | 0.9 (0.6 to 1.4) | 1.0 (0.8 to 1.2) |
| Abbreviations: B[La] = end-set blood lactate concentration; CI = confidence interval; PI = prediction interval; HR_avg_ = average heart rate; %HR_max_ = percentage of maximal heart rate; HR_peak_ = peak heart rate; V̇O_2avg_ = average oxygen consumption; V̇O_2peak_ = peak oxygen consumption; %V̇O_2max_ = percentage of maximal oxygen consumption; T>90% V̇O_2max_ = time above 90% of maximal oxygen consumption; T>95% V̇O_2max_ = time above 95% of maximal oxygen consumption; T90/ET = time above 90% of maximal oxygen consumption/exercise time ratio; T95/ET = time above 95% of maximal oxygen consumption/exercise time ratio; s = seconds; sRPE = session ratings of perceived exertion | | | | | | | | | | | |

**Supplementary Table S10.** Sensitivity analysis: meta-analysed acute physiological and perceptual demands of short-bout high-intensity interval training restricted to protocols with a fixed number of repetitions

**Supplementary Figure S1.** The moderating effects of programming variables on the change in time above 95% of maximal oxygen consumption during short-hiit. abbreviations: * = 1 set of 12 straight-line repetitions, performed at 120% mas for a 15 s work duration with 15 s of passive rest; ci = confidence interval; mas = maximal aerobic speed. note: area outside the shaded zone represents the region of practical significance.

**Supplementary Figure S2.** The moderating effects of programming variables on time above 90% of maximal oxygen consumption during short-HIIT. Red = recreational athletes; orange = trained athletes; light green = national athletes; larger circle = larger study sample; grey zone = 90% confidence interval; MAS = maximal aerobic speed; V̇O_2max_ = maximal oxygen consumption.

**Supplementary Figure S3.** The moderating effects of programming variables on the change in time above 95% of maximal oxygen consumption / exercise time ratio during Short-HIIT. Abbreviations: * = 1 set of 12 straight-line repetitions, performed at 120% MAS for a 15 s work duration with 15 s of passive rest; CI = confidence interval; MAS = maximal aerobic speed. Note: Area outside the shaded zone represents the region of practical significance.

**Supplementary Figure S4.** The moderating effects of programming variables on the change in time above 90% of maximal oxygen consumption/exercise time ratio during short-HIIT. Abbreviations: * = 1 set of 12 straight-line repetitions, performed at 120% MAS for a 15 s work duration with 15 s of passive rest; CI = confidence interval; MAS = maximal aerobic speed. Note: Area outside the shaded zone represents the region of practical significance.
